# Stress-Induced Sphingolipid Signaling: Role of Type-2 Neutral Sphingomyelinase in Murine Cell Apoptosis and Proliferation

**DOI:** 10.1371/journal.pone.0009826

**Published:** 2010-03-23

**Authors:** Raphael Devillard, Sylvain Galvani, Jean-Claude Thiers, Jean-Louis Guenet, Yusuf Hannun, Jacek Bielawski, Anne Nègre-Salvayre, Robert Salvayre, Nathalie Augé

**Affiliations:** 1 INSERM U858, CHU Rangueil, Toulouse, France; 2 Université Paul Sabatier de Toulouse, Faculté de Médecine, Toulouse, France; 3 Université de Bordeaux 2, U.F.R d'Odontologie, Bordeaux, France; 4 Institut Pasteur, Unité de Génétique des Mammifères, Paris, France; 5 Department of Biochemistry and Molecular Biology, Medical University of South Carolina, Charleston, South Carolina, United States of America; Universidade Federal do Rio de Janeiro (UFRJ), Brazil

## Abstract

**Background:**

Sphingomyelin hydrolysis in response to stress-inducing agents, and subsequent ceramide generation, are implicated in various cellular responses, including apoptosis, inflammation and proliferation, depending on the nature of the different acidic or neutral sphingomyelinases. This study was carried out to investigate whether the neutral Mg^2+^-dependent neutral sphingomyelinase-2 (nSMase2) plays a role in the cellular signaling evoked by TNFalpha and oxidized LDLs, two stress-inducing agents, which are mitogenic at low concentrations and proapoptotic at higher concentrations.

**Methodology and Principal Findings:**

For this purpose, we used nSMase2-deficient cells from homozygous *fro/fro* (*fragilitas ossium*) mice and nSMase2-deficient cells reconstituted with a V5-tagged nSMase2. We report that the genetic defect of nSMase2 (in fibroblasts from *fro/fro* mice) does not alter the TNFalpha and oxidized LDLs-mediated apoptotic response. Likewise, the hepatic toxicity of TNFalpha is similar in wild type and *fro* mice, thus is independent of nSMase2 activation. In contrast, the mitogenic response elicited by low concentrations of TNFalpha and oxidized LDLs (but not fetal calf serum) requires nSMase2 activation.

**Conclusion and Significance:**

nSMase2 activation is not involved in apoptosis mediated by TNFalpha and oxidized LDLs in murine fibroblasts, and in the hepatotoxicity of TNFalpha in mice, but is required for the mitogenic response to stress-inducing agents.

## Introduction

Sphingomyelin (SM) is a ubiquitous component of eukaryotic membranes, distributed mainly in the plasma membrane, which contains more than 70–80% of total cellular SM. Sphingomyelinases (SMases) hydrolyze the phosphodiester bond of sphingomyelin to generate phosphorylcholine and ceramide. Ceramides and other metabolic derivatives (e.g. sphingosine and sphingosine-1-phosphate) are lipid “second messengers” molecules involved in the regulation of stress-induced cellular responses, including cell differentiation, proliferation, adhesion and cell death [1], [2].

Apoptosis is a key event in tissue development and in various pathophysiological processes. The role of ceramide in apoptosis [3] and the balance between ceramide and sphingosine-1-phosphate have been largely investigated in various cell types [4]. However, several waves of ceramide production are observed during apoptosis and the mechanisms and roles of the specific ceramide rise are still not well defined, as ceramide generation may result from *de novo* synthesis or/and SM degradation by acid or neutral SMases (aSMase or nSMase) (nSMase being a generic term for an indefinite neutral SMase) [2].

Neutral SMases (nSMases) are activated by a variety of stress-inducing agents, including cytokines, oxidative stress (H2O2, oxidized lipoproteins), UV radiation, chemotherapeutic drugs, β-amyloid-peptides and lipopolysaccharide [5]. The mechanism of nSMase regulation is only partly understood, although several activators and regulators have been identified. Cytokine receptors (e.g. TNF receptor, IL-1 receptor and Fas) and associated proteins (e.g. FAN, RACK-1 and caveolin-1) have been shown to trigger nSMase activation [5], [6]. Reactive oxygen and nitrogen species, GSH depletion, hydrogen peroxide and oxidative stress activate nSMase, while antioxidants, such as reduced glutathione GSH and coenzyme Q are inhibitory [5]. Various cellular mediators regulate nSMase activity including anionic phospholipids, protein kinases phospholipase A2, caveolin, Bcl-2 and Bcl-xL and proteases [5], [7], [8]. Considerable research on nSMase activation, regulation and physiological functions have been carried out, but only little information concerning the specific role of each nSMase is available, because of the relatively recent cloning of mammalian nSMases. Several biological responses are associated to nSMase activation, including inflammation, proliferation, differentiation, cell growth arrest and apoptosis [5], [6], [9], [10]. NSMase1, the first cloned mammalian Mg^2+^-dependent nSMase, is localized in ER and Golgi. However, nSMase1-knockout mice have no apparent phenotype, nor lipid storage [5], [11], [12], though this enzyme could activate a heat-induced and ceramide-mediated apoptotic signaling pathway in zebrafish embryonic cells [13]. NSMase3 is encoded by *SMPD4*. To date, its physiological role remains unknown [14].

The Mg^2+^-dependent nSMase2, encoded by *SMPD3*, is localized in the Golgi and the inner leaflet of the plasma membrane and is implicated in signaling triggered by cytokines, oxidative stress, amyloid β-peptide and endothelial nitric oxide synthase regulation [15], [16], [17], [18]. In MCF-7 cells, nSMase2 is upregulated during cell growth and is required for confluence-induced cell cycle arrest [9]. In smooth muscle cells and fibroblasts, nSMase2 is required for mitogenic signaling and DNA synthesis induced by TNFα and oxidative stress [7], [8]. NSMase2 has been implicated in apoptosis induced by TNFα associated with cycloheximide in MCF-7, by staurosporine or C2-ceramide in neurotumor cell lines, and by H2O2 in human aortic endothelial cells and in airways epithelial cells [5]. The deletion in *smpd3* gene is associated with an osteogenesis imperfecta phenotype in *fragilitas ossium* (*fro*) mice [19], while mice knock-out for nSMase2 exhibit neonatal growth retardation associated with chondrodysplasia [20]. Neither *fro/fro* nor nSMase2-KO mice exhibit any SM storage in brain and organs, and no defects of apoptosis are observed in *fro/fro* [19], [20].

Stress-inducing agents such as TNFα and oxidized LDLs (oxLDLs) trigger a huge variety of cellular responses, among them proliferation, inflammatory signaling and apoptosis [21], [22], [23]. OxLDLs exhibit a clear biphasic effect, since low concentration of oxLDLs is mitogenic, whereas higher concentration is toxic[24]. Both TNFα and oxLDLs trigger nSMase activation, SM hydrolysis, and ceramide generation, potentially involved in apoptotic signaling. Since the precise identity and roles of the nSMase are not clearly defined, this study was designed to evaluate the role of nSMase2 in apoptosis and growth responses induced by these two stress-inducing and cytotoxic agents in fibroblasts. The use of nSMase2-deficient cells from *fro/fro* mice, strongly suggests that this nSMase is not necessary for TNFα and oxLDLs induced apoptosis in these cells but is required in the mitogenic signaling triggered by both agents.

## Methods

### Chemicals

[^3^H]Thymidine (79 Ci/mmol), [*methyl*-^3^H]choline (70 Ci/mmol) and [*choline-methyl-^14^C*] sphingomyelin (52 mCi/mmol) were from Perkin Elmer (Wellesley, US). RPMI 1640 containing glutamax, DMEM, fetal calf serum (FCS) was from Invitrogen (France). Human recombinant TNFα was from Abcys (Annecy, France). Ac-Asp-Glu-Val-Asp-aminomethylcoumarin (Ac-DEVD-AMC) was from Bachem (Voisins-Le-Bretonneux, France). Anti Caspase-3 was from cell signaling (Ozyme, Saint-Quentin-en-Yvelines). Other antibodies and reagents were obtained from Sigma (Lisle-d'Abeau, France).

### Animals and treatments

The genetic background of *fro/fro* and wt mice was 129/SV. Homozygous *fro/fro* mice, harboring a truncating mutation in nSMase2 and *fragilitas ossium (fro)* phenotype [19], were genotyped by PCR, as previously described [19], using the following primers: 5′-GCCCGCAGCCATGTATAGTA-3′, 5′-CTCAATGGAGGGCACACAG-3′ and 5′-CAGGTTTAGGGACCCTGACG-3′.

Wt and *fro/fro* animals (10 wt and 10 *fro/fro*, 5 males and 5 females in the TNFα-treated group) were housed under specific pathogen-free conditions, maintained on a 12∶12 h light-dark cycle with lights on at 07.00, and free access to food and water (IFR-150, Toulouse, France). At the age of 7–10 weeks, wt and *fro/fro* mice (weight ∼25–30 g) were injected intraperitoneally with a single dose of D-galactosamine (800 mg/kg; Sigma) followed by intravenous injection of murine recombinant TNFα (Abcys®) (40 µg/kg of body weight) in a total volume of 0.1 ml PBS containing 1% bovine serum albumin. Mice were sacrificed under anesthesia at designated time points for histology and biochemical. The right lateral lobe of the liver was kept for histology, and the remainder of the tissue was immediately frozen in liquid nitrogen for biochemical analysis.

The experimental protocol (N° 06/858/10/06) was approved by the institutional ethical committee for animal experiments.

### LDL preparation and oxidation

Human LDLs (*d* 1.019–1.063) were isolated from pooled fresh sera by sequential ultracentrifugation, dialyzed, sterilized by filtration, and oxidized by UV-C irradiation. Mildly oxLDLs were obtained by UV oxidation as previously described [24].

### Cell culture

Primary cultures of fibroblasts were obtained by skin biopsies from newborn control and *fro/fro* mice. Briefly, skin samples were minced and put in Petri dishes, dermis facing down. After 15 min. of dry contact with the dishes, DMEM culture medium containing 20% FCS/penicillin/streptomycin/amphotericin A was added, and the skin preparation was cultured at 37°C/5%CO2. After 1 to 3 weeks, cells growing around the tissue pieces were expanded, and rapidly underwent spontaneous immortalization, as frequently reported for primary rodent cells [25]. NSMase2 activity of immortalized fibroblasts was similar to that of the respective (‘primary’ non immortalized) early passages of cultured fibroblasts and was stable over the successive passages (in particular, the severe deficiency of nSMase2 activity remained unchanged in *fro/fro* cells).

Control (wt) or *fro/fro* fibroblasts were routinely grown in DMEM supplemented with 10% heat-inactivated fetal calf serum (FCS). Before the addition of stress-inducing agents, cells were starved overnight in serum-free RPMI-1640 (because the toxicity of oxLDLs is higher in this medium).

### Cell transfection

Plasmid containing murine V5-nSMase2 (V5-tagged *smpd3*) [9] cDNA was transfected into *fro/fro* fibroblasts as previously described [8]. Briefly, V5-tagged nSMase2 cDNA cloned into the eukaryotic expression vector pEF6/V5-His was transfected into *fro/fro* fibroblasts by using Lipofectamin reagent (Invitrogen), as reported [8]. Stable transfectants were selected by adding 10 µg/ml blasticidin (Invitrogen) to the culture medium. 6 independent colonies were picked up and cultured in separate wells, and the nSMase activity was quantified. Three clones expressing nSMase activity at least 3 times higher than wt fibroblasts were selected: in the paper the experiments were performed with ‘fro-V5smpd3 clone 3’. Mock-transfected *fro/fro* cells were prepared with the empty pEF6/V5-His vector, and did not exhibit any increased nor stimulable nSMase activity, and were used as control. In order to maintain the selection pressure, the transduced cells were treated again by blasticidin, every 6 passages.

### Determination of nSMase activity and cellular sphingomyelin hydrolysis

The activity of nSMase was determined in cell extracts (100 µg protein) in the presence of radiolabeled [choline-methyl-^14^C]sphingomyelin and unlabeled sphingomyelin, as previously reported [26]. Briefly, cells were harvested and homogenized by sonication in 0.1% Triton X-100, 10 mM MgCl_2_, 5 mM dithiothreitol, 0.1 mM Na_3_VO_4_, 10 mM glycerophosphate, 750 µM ATP, 1 mM PMSF, 2 mM EDTA, 10 µM leupeptin, and 10 µM pepstatin. Then, 100 µl of substrate containing [choline-methyl-^14^C]sphingomyelin (120,000 dpm/assay) and 20 nmoles of unlabelled sphingomyelin/assay in 0.1% Triton X-100, 20 mM HEPES buffer, pH 7.4, containing 1 mM MgCl2 was added to 100 µl of cell homogenate. After 2 h of incubation at 37°C, the liberated [methyl-^14^C]choline was partitioned under the previously used conditions [26] and determined by liquid scintillation counting.

Cellular sphingomyelin was radiolabeled by preincubating cells with [*methyl*-^3^H]choline for 48 hours. After treatment with stress agents, the level of radiolabeled sphingomyelin was quantified, at the indicated time, under the previously used conditions [26].

### Sphingolipid content analysis

Sphingolipids were extracted with chloroform/methanol from control and TNFα or oxLDLs-stimulated wt and *fro/fro* fibroblasts, and from wt and *fro/fro* mice livers injected with TNFα. Aliquots of lipid extracts were used for determining total phospholipid and sphingomyelin contents by measuring inorganic phosphorus before and after mild alkaline methanolysis[26]. The ceramide and S1P contents were determined by HPLC, as reported [27].

### DNA synthesis, cell viability and apoptosis

DNA synthesis was evaluated by [^3^H]thymidine incorporation under previously described conditions [8].

The overall toxicity was evaluated by the MTT assay[24].

Apoptotic/necrotic cells were counted by fluorescence microscopy after staining by fluorescent DNA intercalating agents SYTO-13 and propidium iodide (PI) [28]. Briefly, cells grown in 6-multiwell plates were incubated with the permeant DNA intercalating green fluorescent probe SYTO-13 (0.6 µM) and the non permeant DNA intercalating red fluorescent probe PI (15 µM), using an inverted fluorescence microscope (Fluovert FU, Leitz). Intact, apoptotic and necrotic cells were characterized on the basis of their morphological features: Normal nuclei exhibit a loose green colored chromatin, nuclei of primary necrotic cells exhibit a loose red colored chromatin, nuclei of apoptotic cells exhibited fragmentation associated with condensed yellow/green-colored chromatin, while post-apoptotic necrotic cells exhibited the same morphological features, but were red-colored. 200 cells/well were counted, each experiment being performed at least in triplicate. Alternatively, flow cytometry experiments after annexin-V-FITC labeling were performed to evaluate phosphatidylserine externalization, an early event of apoptosis. Briefly, fibroblasts were gently trypsinized for 30 s and immediately added to 10% FCS-containing DMEM, collected and pooled with non-attached cells. The cells were harvested, washed, and stained with Annexin V-fluorescein isothiocyanate and propidium iodide for 10 min at 4°C in the dark (Apoptosis Detection Kit; R&D Systems) (Annexin V-FLUOS Kit; Roche, Mannheim,Germany) according to the manufacturer's instructions. After being stained, cells were immediately analyzed on a FACScan (BD Biosciences) cytofluorometer. At least 20 000 cells were analyzed *per* sample. All experiments were repeated at least three times. Data analysis was performed with Cell Quest software (Becton Dickinson).

### Fluorometric assay for caspase (DEVDase) activity

DEVDase (caspase) activity was determined using the fluorogenic substrate Ac-DEVD-AMC (N-acetyl-Asp-Glu-Val-Asp-7-amino-4-methylcoumarin). Cells were lyzed in ice-cold lysis buffer (10 mM HEPES (pH 7.4), 42 mM KCl, 5 mM MgCl_2_, 0.5% CHAPS, 1 mM dithiothreitol, 1 mM phenylmethylsulfonyl fluoride, and 2 µg/ml leupeptin). The assay mixture containing 100 µL of the cell lysate and 100 µL Ac-DEVD-AMC substrate (final concentration 20 µmol/L) was incubated for 30 min at 25°C, and the released fluorescent product AMC was determined by fluorometry (excitation and emission wavelengths, 355 and 440 nm, respectively).

### Immunocytochemistry

Cells grown on uncoated glass coverslips were fixed in 3% paraformaldehyde for 15 min and permeabilized with 0.1% Triton X-100, then incubated with the indicated antibodies and finally examined by fluorescence microscopy, as previously reported [26].

### Liver histology

Paraffin-embedded liver specimens from control and TNFα-treated wt and *fro/fro* mice were processed and stained with hematoxylin/eosin using standard laboratory methods, which allowed to score the extent of hepatocellular necrosis.

### Statistical analysis

Data are given as mean ± SEM. Estimates of statistical significance were performed by Anova (Tukey test - SigmaStat software), values of P<0.05 being considered significant.

## Results

### Apoptotic or mitogenic effects of TNFα and oxLDLs on murine fibroblasts

TNFα and oxLDLs are known to trigger the activation of the SM/Cer pathway and to induce both mitogenic and apoptotic responses in a dose-dependent manner and through different mechanisms [1], [3], [8], [29].

**Figure 1:**
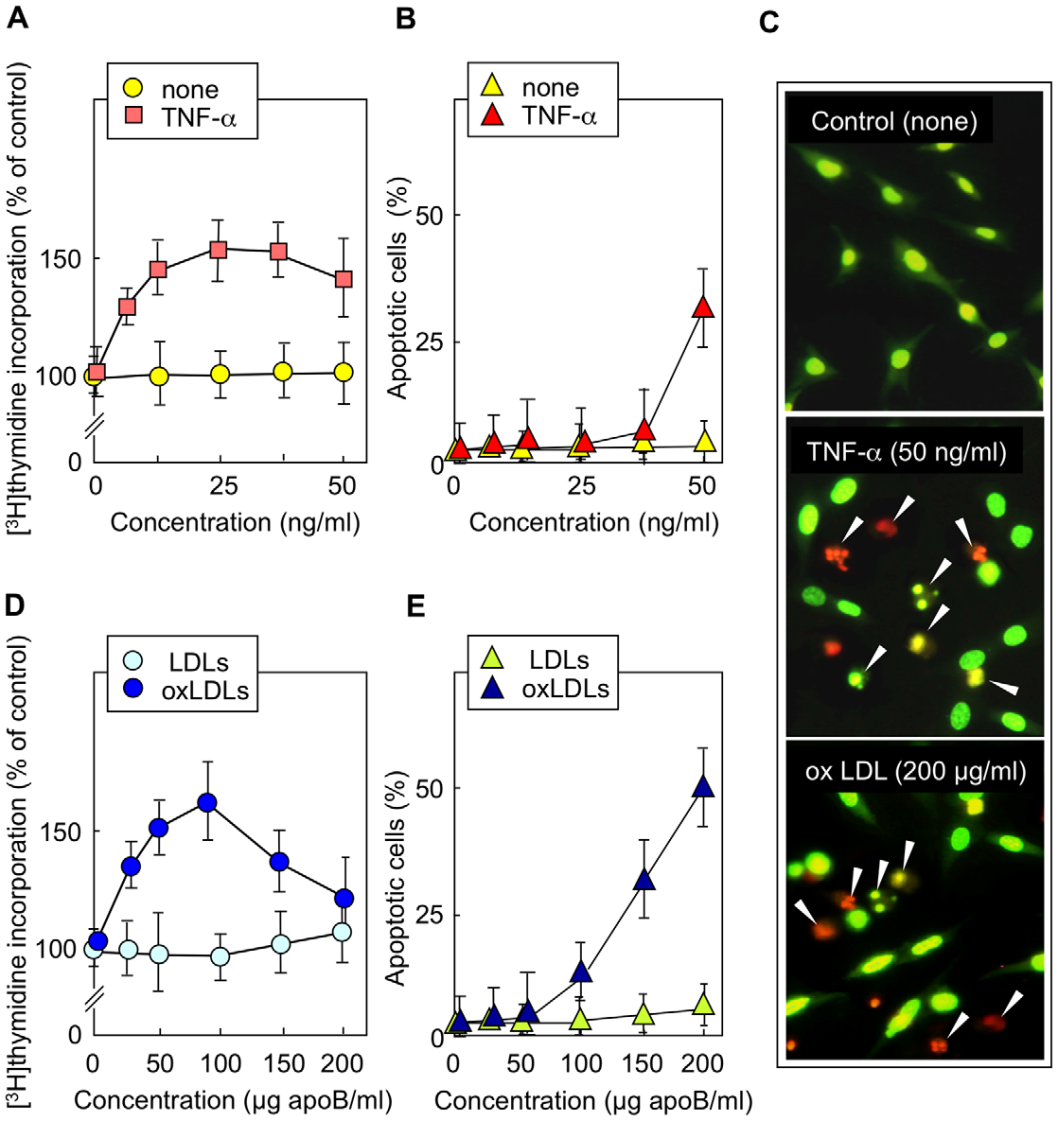
TNFα and oxLDLs induce dose-dependent apoptotic or mitogenic effects in wt murine fibroblasts. Wild-type (wt) murine fibroblasts were starved in serum-free medium for 24 h, then were treated for 24 h with the indicated concentration of TNFα (0–50 ng/ml) or of LDL non oxidized (LDLs) and oxidized (oxLDLs) (0–200 µg/ml). DNA synthesis was quantified by [^3^H]thymidine incorporation and was expressed as % of untreated control (A,D). Apoptosis and necrosis were visualized by fluorescence microscopy of cells stained by Syto13/PI (B,C,E). Primary apoptosis was characterized by condensed picnotic or fragmented nuclear stained green/yellow by Syto13, whereas post-apoptotic necrosis exhibited the same nuclear morphology but was stained red by PI (permeabilization of the plasma membrane in a late step of apoptosis). Only few cells exhibited the feature of primary necrosis, i.e. loose chromatin stained red. In, A,B,D,E, Mean ± SEM of 4 experiments. *: p<0.05.

Preliminary experiments have shown that low concentrations of TNFα are mitogenic for fibroblasts [8] while higher concentrations are cytotoxic (Figure 1). The toxic effect of TNFα is generally evaluated in the presence of cycloheximide. In our system, cycloheximide, under the conditions usually utilized in the literature, sensitized murine fibroblasts to the toxic effect of TNFα, but was also toxic *per se* (data not shown). This led us to use TNFα alone (in the absence of cycloheximide), in order to avoid confusing data in the study of the toxicity (the mechanisms of action of TNFα and cycloheximide being different, at least in part). It may be noted that, in our experimental system (mouse fibroblasts) human and mouse recombinant TNFα display similar effects (data not shown), but in order to reduce the complexity of pro-apoptotic signaling by TNFα [21], [22], [23], we used human recombinant TNFα (hrTNFα), which is known to activate nSMase through only TNF-R1, since murine TNF-R2 is insensitive to hrTNFα [30]. Moreover, it may be noted that the expression of TNF receptor 1 (TNFR1) was similar in wt and *fro/fro* fibroblasts (data not shown).

As shown in Figure 1, low concentrations of TNFα and oxLDLs triggered a mitogenic response, evaluated by [^3^H]thymidine incorporation, which was maximal at 20–30 ng/ml of TNFα and 50 µg apoB/ml of oxLDLs. In contrast, under the used conditions, the toxic effect required higher concentrations of the agonists, 50 ng/ml of TNFα and 100–200 µg apoB/ml of oxLDLs. Apoptosis was evaluated by fluorescence microscopy after cell staining by SYTO13/PI (Figure 1B,C,E). Primary apoptosis was characterized by condensed, pyknotic or fragmented nucleus stained green/yellow by SYTO13 whereas post-apoptotic necrosis exhibited the same nuclear morphology but was stained red by PI (permeabilization of the plasma membrane of apoptotic cells). Few cells exhibited the feature of primary necrosis, i.e. loose chromatin stained red. Flow cytometry experiments performed on fibroblasts incubated in the presence or absence of 200 µg apoB/ml oxLDLs, or 50 ng/ml TNFα, or 100 nmol/l staurosporine, were correlated with fluorescence microscopy counting, and indicated a comparable number of Annexin-V-positive cells for both wt and *fro/fro* cells (Figure S1). Thus the agents were similarly toxic for wt and *fro/fro* cells, whatever the technique (microscopy counting of dead cells or flow cytometry).

### SM/Cer pathway activation by TNFα and oxLDLs in murine fibroblasts requires nSMase2

**Figure 2:**
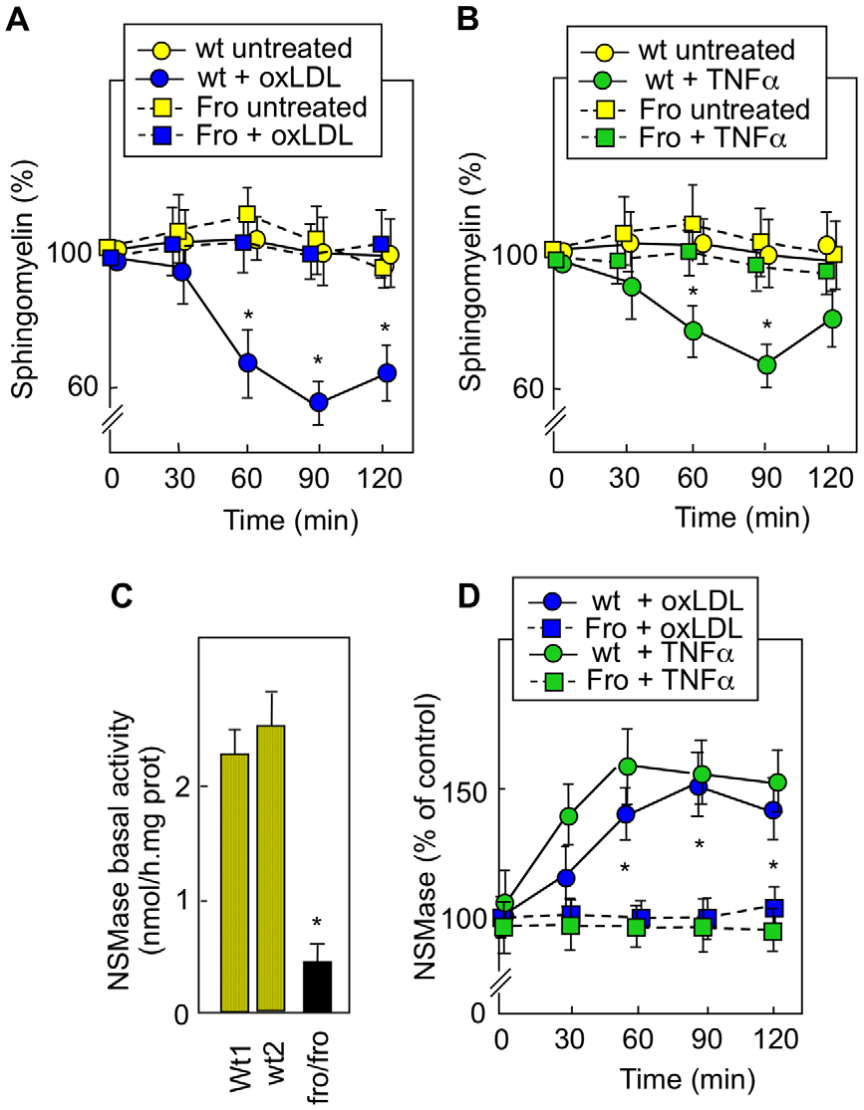
TNFα and oxLDLs induce nSMase activation in *wt* but not in *fro/fro* fibroblasts. A,B - Sphingomyelin hydrolysis was monitored after metabolic labeling using [^3^H]choline chloride (0.5 mCi/ml) of cells, as described in the Method section. Then, cells were stimulated with oxLDLs (200 µg/ml) (A) or TNFα (50 ng/ml) (B), and the level of cellular sphingomyelin was determined at the indicated time. Results are expressed as % of control (time 0 h of stimulation). C,D -Basal nSMase activity in untreated cells (C) and in cells treated with TNFα (50 ng/ml) or oxLDLs (200 µg/ml) for the indicated time (D). Mean ± SEM of 3 to 5 separated experiments. * p<0.05.

In order to define the specific contributions of nSMase2 to these stress responses, the activation of the SM/Cer pathway by TNFα and oxLDLs was investigated on fibroblasts from nSMase2-deficient *fro/fro* mice [19]. As expected, TNFα *and* oxLDLs triggered both SM hydrolysis (Figure 2A,B) and nSMase activation in wt fibroblasts (Figure 2D). In contrast, in *fro/fro* fibroblasts, SM hydrolysis (Figure 2A,B), basal nSMase activity (Figure 2C), and nSMase activation triggered by the two agonists (Figure 2D) were deficient. Likewise, the ratio S1P/ceramide measured in wt and *fro/fo* fibroblasts was increased two to three times in wt fibroblasts stimulated either by oxLDLs or TNFα, while no variation was observed in *fro/fro* cells (data not shown). Altogether, these data strongly suggest that nSMase2, which is mutated and deficient in *fro/fro* mice [19], is activated and required for the activation of the SM/Cer pathway elicited by TNFα and oxLDLs in fibroblasts (basal nSMase activity detected in fro/fro cells probably resulting from the expression of other nSMases [2] that are apparently not activated by TNFα and oxLDLs under the experimental conditions used here). In these cells, the early phase (30–120 min) of SM hydrolysis and ceramide formation appears to be mainly attributable to nSMase2 activation.

### TNFα and oxLDLs-induced apoptosis is independent of nSMase2 activity

**Figure 3:**
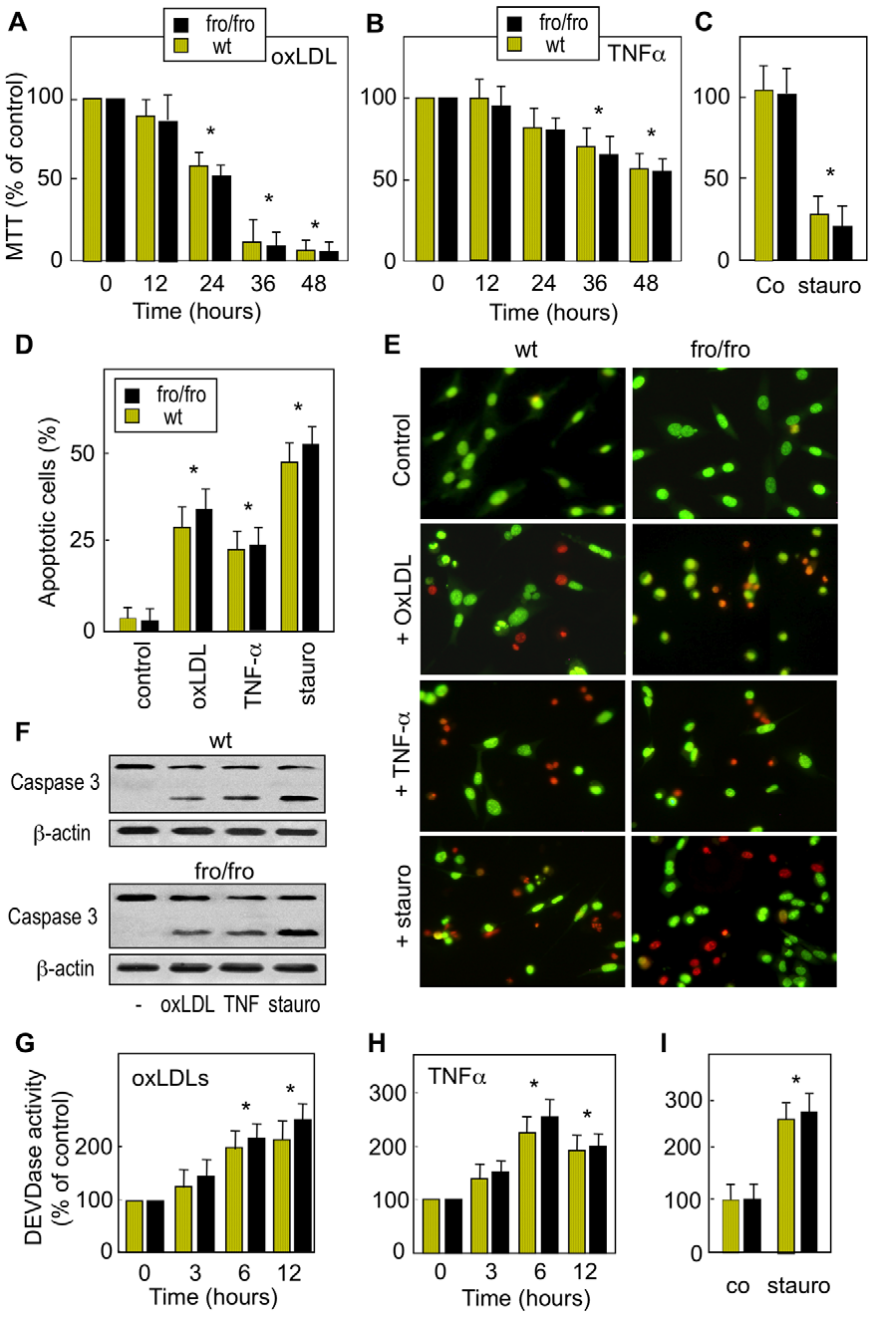
TNFα, oxLDLs and staurosporine induce cell apoptosis in wt and *fro/fro* fibroblasts. Fibroblasts were incubated with TNFα (50 ng/ml, 48 h), oxLDLs (200 µg/ml, 24 h) or staurosporine (100 nM, 6 h). Cell viability was evaluated by the MTT assay (A–C) and by counting apoptotic cells after syto13/PI labeling (D,E), as in Fig. 1. Caspase 3 activation was determined by western blot showing pro-caspase (32 kDa) and cleaved active caspase (17 kDa) (F). Time-course of DEVDase activity were measured using the fluorogenic substrate Ac-DEVD-AMC in cells treated by oxLDLs, TNFα and staurosporin, respectively (G–I). The results are mean ± SEM of 3 to 5 separated experiments. * p<0.05 for apoptotic cell counting and DEVDase activity measurement (comparison between cells treated with or without agonist).

We then investigated whether nSMase2 is required for apoptosis induced by TNFα, oxLDLs, or staurosporine, by comparing the apoptotic effect of these agents in wt and *fro/fro* fibroblasts (Figure 3). The time-course of oxLDLs- and TNFα-induced cell death (50% mortality observed after 24h with oxLDLs, and 48 h with TNFα) was assessed by the MTT assay to evaluate the overall toxicity (Figure 3A–D). Dying cells exhibited the morphological features of apoptotic cells, revealed by fluorescence microscopy after SYTO13/PI staining (Figure 3E). Under the used conditions (apoptotic stress triggered by toxic concentrations of TNFα, oxLDLs and staurosporine), *fro/fro* cells underwent apoptosis similar to wt cells (and were even found more sensitive) (Figure 3E). Consistent with the morphological data, the apoptotic agents induced DEVDase activation, (time-course was evaluated by the hydrolysis of the fluorogenic substrate Ac-DEVD-AMC) (Figure 3G), and caspase-3 cleavage, (evaluated by western blot) (Figure 3F). These effects occurred at a similar extent in wt and *fro/fro* fibroblasts (Figure 3). All these data, suggest that nSMase2 is not required for apoptosis induced by TNFα, oxLDLs and staurosporine in murine fibroblasts.

**Figure 4:**
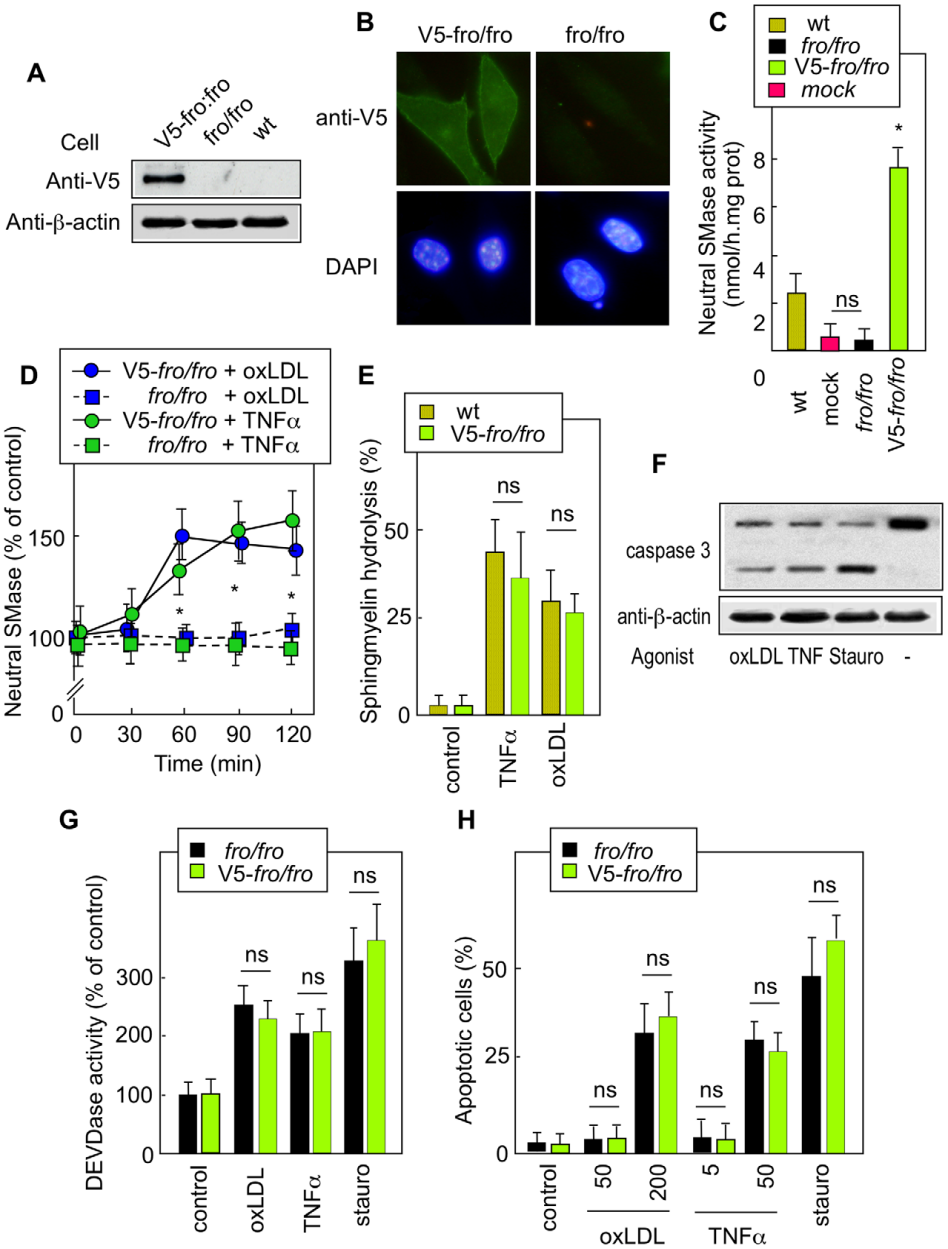
Effects of TNFα and oxLDLs on *fro/fro* fibroblasts transfected with active V5-nSMase2 vector. The *fro/fro* fibroblasts were stably transfected using pEF-6 plasmid containing V5-nSMase2 cDNA. After clone selection, the expression of V5-nSMase2 was evaluated by western blot using an anti-V5 antibody (A), immunocytochemistry (B) and by enzymatic determination of nSMase activity, under basal conditions (C) and after activation by TNFα (50 ng/ml) or oxLDLs (200 µg/ml) (D). Time course of sphingomyelin hydrolysis induced by TNFα (50 ng/ml) or oxLDLs (200 µg/ml) were determined in V5-transfected cells under the conditions indicated in the legend to Figure 2 (E). Caspase activity was evaluated by western blot of caspase-3 (32 KDa) (F), and by fluorometric determination of DEVDase activity (G), under the conditions of Fig. 3. Apoptosis triggered by TNFα (50 ng/ml, 48 h incubation) or oxLDLs (200 µg/ml, 24 h incubation) was evaluated by fluorescence microscopy counting of cells labeled by syto13/PI, under conditions of Fig. 1 and 3 (H). In Fig. 4C–E, and G,H, the data are expressed as mean ± SEM of 3 to 5 separated experiments * p<0.05; ns, not significant.

In *fro/fro* fibroblasts expressing a V5-tagged nSMase2 (*fro*-V5 clone 3 fibroblasts) (Figure 4A), the basal level of nSMase activity was dramatically increased (around 3 times the level of wt) (Figure 4C). NSMase activation and sphingomyelin hydrolysis by TNFα and oxLDLs were also rescued in V5-*fro* (Fig. 4D,E). Note that mock-transfected *fro/fro* cells did not exhibit any change in basal nor stimulable nSMase activity (Fig. 4C). In spite of the broad variation in nSMase activity, the activation of caspase 3 and the apoptotic effect of TNFα, oxLDLs and staurosporine were similar in *fro/fro* and V5-*fro* fibroblasts (Figure 4E–H). Taken together, all these data allow concluding that murine fibroblasts do not require nSMase2 for cell death induction in response to TNFα, oxLDLs, or staurosporine.

### The nSMase2 defect in fro/fro mice does not prevent TNFα toxicity

**Figure 5:**
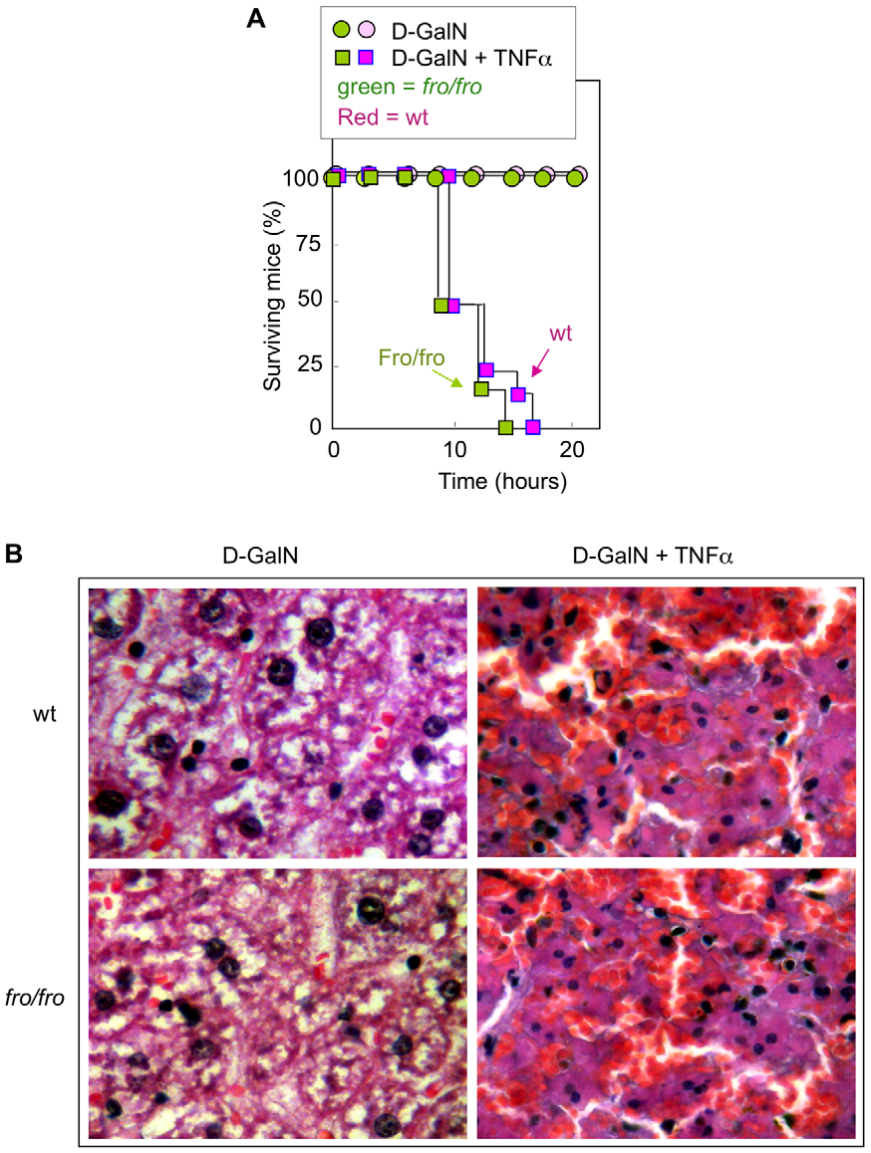
Time course of TNFα-induced toxicity in wt and *fro/fro* mice. Mice (10 wt or 10 *fro/fro*, 5 females and 5 males) were intraperitoneally injected with D-galactosamine (20 mg) and then injected intravenously with PBS or TNFα (40 µg/kg of body weight). A -Time course of survival of wt (red symbols) and *fro/fro* (green) mice treated or not by D-galactosamine and TNFα. B - Histological analysis of hematoxylin-eosin stained liver sections from wt or *fro/fro* mice injected with D-galactosamine/PBS (sacrificed 48 hours after the injection) or D-galactosamine/TNFα (immediately taken off after mice death). Representative microscopy pictures of liver sections from wt and *fro/fro* mice (magnification, ×400).

Because TNFα may trigger liver injury and lethal toxicity through a mechanism involving ceramide generation [22], we compared the susceptibility of *fro/fro* and wt mice to the toxic effects of TNFα (40 µg/kg) after pretreatment with the transcription inhibitor D-galactosamine (D-GalN). In this model, D-galactosamine was used to avoid the expression of anti-apoptotic genes and thus to sensitize hepatic cells to TNFα, but D-galactosamine exhibited no lethal effect *per se* (i.e. in the absence of TNFα). Both *fro/fro* and wt mice were similarly sensitive to TNFα-induced lethality (Figure 5A). The hepatic ceramide content, expressed as the ratio to liver sphingomyelin, was 1.5 to 2 fold increased in TNFα-treated wt and *fro/fro* mice, thereby indicating that sphingomyelin hydrolysis mediated by TNFα treatment, involves another sphingomyelinase, probably an acidic SMase as reported [24], but not nSMase2. Histological study showed denser and more eosinophilic cytoplasm and pyknotic nuclei, associated with diffuse hemorrhagic areas, and the results were similar for both wt and *fro/fro* TNFα-treated mice, (Figure 5B). It must be noted that lower TNFα doses (10 µg/kg) were not toxic for both wt and *fro/fro* mice over 48 h (data not shown).

### The activation of nSMase2 is required for TNFα and oxLDLs-induced cell proliferation

**Figure 6:**
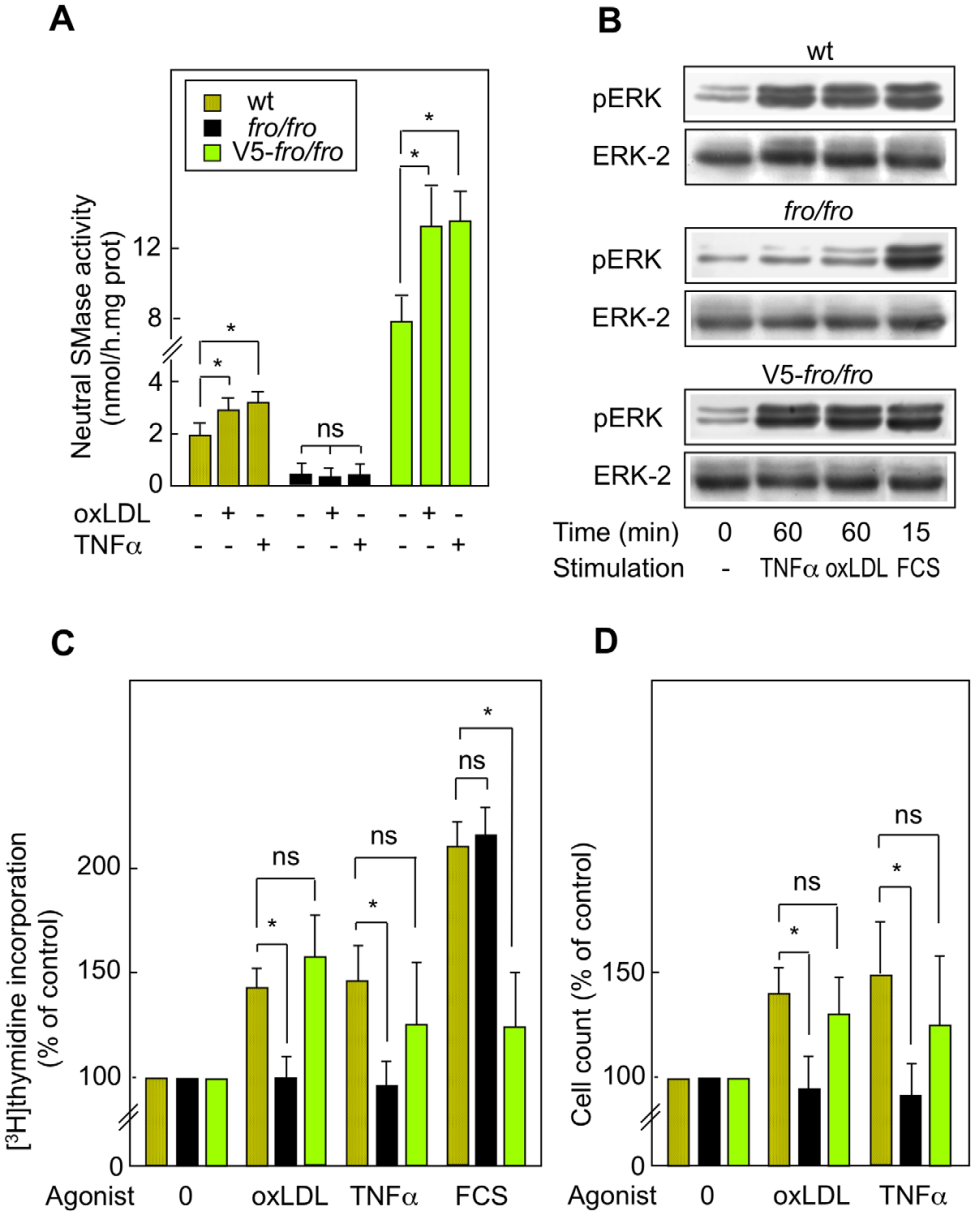
Proliferation mediated by TNFα or oxLDLs requires the activation of the nSMase2. A,B - TNFα (5 ng/ml) and oxLDLs (50 µg/ml) induced nSMase activation (A) and ERK1/2 phosphorylation (B) in wt and in V5-*fro*) but not in *fro/fro* fibroblasts. It may be noted that FCS (10%) induce ERK1/2 phosphorylation in the 3 cell types, independently of nSMase2 deficiency (B). C,D - Proliferation was evaluated by DNA synthesis ([^3^H]thymidine incorporation after 24 h incubation with the agonists) and cell count (performed after 48 h incubation with the agonists), in wt, *fro/fro* and V5-*fro* fibroblasts, after treatment by TNFα, oxLDLs and FCS, as in Fig. 6A,B. In A,C,D, mean ± SEM of 3 to 5 separated experiments. * p<0.05 (comparison between cells treated with or without TNFα, as indicated).

Since the above data suggest that nSMase2 is not involved in the apoptotic effect of oxLDLs and TNFα, and since nSMase activity may play a role in the mitogenic effect of oxLDLs [26] and TNFα [8], we investigated whether the genetic defect of nSMase2 (*fro/fro*) and the nSMase2 rescue (V5-*fro*) modulate the mitogenic effect mediated by these agonists. As shown in Figure 6, low non-toxic concentrations of oxLDLs and TNFα triggered nSMase activation, (and sphingomyelin hydrolysis, data not shown), mitogenic signaling, DNA synthesis, and cell proliferation in wt fibroblasts. In contrast, the mitogenic effect of oxLDLs and TNFα was abolished in *fro/fro* fibroblasts, as assessed by the lack of ERK1/2 activation and DNA synthesis (Figure 6B,C). In contrast, in V5-*fro* fibroblasts expressing V5-nSMase2, the activation of nSMase was rescued (Figure 6A), as well as the activation of ERK1/2, increased DNA synthesis, and cell proliferation (Figure 6B–D).

These data show that nSMase activation by oxLDLs and TNFα is dependent on the activity of nSMase2 (Figure 6A) and is linked to the mitogenic signaling triggered by the two agonists (Figure 6C,D). This strongly suggests that nSMase2 is required for the mitogenic response to the stress agonists oxLDLs and TNFα.

Interestingly, the FCS-induced mitogenic effect was similar in wt and *fro/fro* fibroblasts, thereby suggesting that nSMase2 activity is not required for the mitogenic effect of growth factors contained in the FCS (Figure 6B,C).

## Discussion

TNFα and oxLDLs trigger the activation of nSMase2 in murine wt fibroblasts, but the apoptotic effect of these agonists is apparently independent of nSMase2 and SM hydrolysis, since the genetic defect of nSMase2 (in cells from *fro/fro* mice) does not alter the apoptotic response. Moreover, the hepatic toxicity triggered *in vivo* by TNFα is similar in wt and *fro/fro* mice, thus suggesting that nSMase2 is not required for the TNFα-induced hepatic apoptosis. In contrast, our data show that nSMase2 is absolutely required for the mitogenic effect induced by low concentration of TNFα and oxLDLs.

A variety of data have been reported on signaling and biological responses associated with the stimulation of nSMases, but only few studies have addressed the specific nSMase that is implicated [5], [6]. In the last decade, three mammalian nSMases have been cloned [11], [14], [31], but their specific roles are only partly known. Precisely, the apoptotic role of nSMase2 needs to be re-evaluated [5], because of the unexpected phenotype of nSMase2-deficient mice, that exhibit neonatal growth retardation and bone disease, but no obvious defect of apoptosis [19], [20]. Thus, our study was designed to evaluate whether nSMase2 plays a role in apoptosis induced by TNFα and by oxLDLs.

For this study, we chose TNFα and oxLDLs, two agents known to activate nSMase, via different signaling mechanisms. TNFα is a prototypical trigger of cellular nSMase activation (and ceramide generation) associated with pleiotropic responses, including apoptosis, cell proliferation and inflammation [5]. As indicated above, TNFα was used without any protein synthesis inhibitor, in order to exclude interference due to the cytotoxicity of cycloheximide (or other protein inhibitor). The apoptotic effect of TNFα was apparently independent of nSMase2 and SM hydrolysis in murine fibroblasts, since similar apoptosis was observed in nSMase2-deficient cells, and since rescuing the activity of the nSMase2 did not alter the apoptosis induced by TNFα and oxLDLs. Apoptosis by TNFα is mediated by FADD and caspase 8 which in turn activates caspase-3 and/or Bid and the mitochondrial apoptotic pathway [32]. An alternative signaling pathway involving the generation of ceramide, either through the *de novo* synthetic pathway, or by sphingomyelin hydrolysis by SMases, has been hotly debated [10], [33]. The role of the acid SMase in ceramide generation and apoptosis triggered by TNFα is largely documented [34], [35], but nSMases have also been implicated in the regulation of apoptosis by TNFα [36]. Our data allow to conclude that nSMase2 is not required for TNFα-induced apoptosis and that its activation by TNFα is not necessary for triggering apoptosis in fibroblasts. However, it cannot be excluded that nSMase2 may play a pro-apoptotic role in other cell types, as suggested by its mutation in a murine osteosarcoma cell line and in human leukemias [37]. This discrepancy may result from factors regulating the level of ceramide, for instance, subcellular location, transport and metabolic conversion, from the balance of ceramide/sphingosine-1-phosphate, and from cell type-dependent expression of pro-/anti-apoptotic effectors (e.g. Bcl2 family members) [2], [4].

Apoptosis triggered by oxLDLs was also found to be independent of nSMase2 in *fro/fro* fibroblasts. This is consistent with the lack of involvement of early ceramide formation and the central role of calcium [38] in oxLDLs-induced apoptosis [39], [40], [41].

In contrast, nSMase2 was required for the mitogenic response triggered either by TNFα or oxLDLs in murine fibroblasts. Previous reports had shown that the mitogenic response mediated by both stress-inducing agents, depends on furin and metalloproteinases MT1-MMP and MMP-2 which activate nSMase2, sphingomyelin hydrolysis, as well as the generation of sphingosine-1-phosphate by sphingosine kinase [8], [26]. The lack of TNFα- or oxLDLs-induced mitogenic effect in *fro/fro* fibroblasts strongly suggests that nSMase2 activation and ceramide production are crucial for this stress-induced response. Note that the acid SMase, which is not deficient in *fro/fro* fibroblasts (data not shown), is unable to compensate for the defect in nSMase2-deficient cells. This is in agreement with the idea that the topology of ceramide generated by sphingomyelinases may play a crucial functional role. Ceramide generated by the acid SMase in the outer leaflet of the plasma membrane forms ceramide-enriched membrane platforms that promote death-domain receptor clustering, thereby amplifying apoptotic signalling [42]. In contrast, nSMase2 is located on the inner leaflet, where it generates ceramide, hydrolyzed by ceramidases into sphingosine, which may be phosphorylated in turn by sphingosine kinase-1 [2], [4]. This is consistent with the hypothesis that the sphingolipid pathway located in the inner leaflet may generate sphingosine-1-phosphate potentially involved in cell activation, leading to cell proliferation or inflammatory response [4].

Finally, *in vivo* experiments showed that nSMase2 plays apparently no major role in hepatic apoptosis triggered by TNFα (associated to galactosamine), since wt and *fro/fro* mice exhibited similar hepatotoxicity and death rate. This is in agreement with *in vitro* studies, and with previous reports indicating a major role for acid SMase and the FADD-caspase pathway in TNFα-induced hepatocellular apoptosis [22], [43], [44].

## Supporting Information

Figure S1 Flow cytometry. A Determination of cell death by flow cytometry of annexin V-FITC/PI staining of wild-type (wt) and fro/fro murine fibroblasts were seeded in 6-well plates for 48 hours and then exposed to TNFα (50 ng/ml) or oxLDLs (200 µg/ml) for 24 hours or staurosporine (100 nM) for 6 hours. Then, cells were harvested and stained as indicated in Material and Methods section, and immediately used for cytometry determination. Representative dot-plot graphs of 3 independent experiments. The percentages of dying cells is calculated from all annexin-V-positive cells, because microscopy examination shows that annexin-V/PI double positive cells exhibited the morphology of post-apoptotic necrosis. B Percentage of annexin V-positive wt and fro/fro murine fibroblasts treated by oxLDLs, TNFα or staurosporine. Data are expressed as mean ± SEM from 3 separate experiments.
(1.21 MB TIF)

## References

[1] Hannun YA (1996). Functions of ceramide in coordinating cellular responses to stress.. Science.

[2] Hannun YA, Obeid LM (2008). Principles of bioactive lipid signalling: lessons from sphingolipids.. Nat Rev Mol Cell Biol.

[3] Kolesnick RN, Kronke M (1998). Regulation of ceramide production and apoptosis.. Annu Rev Physiol.

[4] Spiegel S, Milstien S (2003). Sphingosine-1-phosphate: an enigmatic signalling lipid.. Nat Rev Mol Cell Biol.

[5] Clarke CJ, Hannun YA (2006). Neutral sphingomyelinases and nSMase2: bridging the gaps.. Biochim Biophys Acta.

[6] Auge N, Negre-Salvayre A, Salvayre R, Levade T (2000). Sphingomyelin metabolites in vascular cell signaling and atherogenesis.. Prog Lipid Res.

[7] Coatrieux C, Sanson M, Negre-Salvayre A, Parini A, Hannun Y (2007). MAO-A-induced mitogenic signaling is mediated by reactive oxygen species, MMP-2, and the sphingolipid pathway.. Free Radic Biol Med.

[8] Tellier E, Negre-Salvayre A, Bocquet B, Itohara S, Hannun YA (2007). Role for furin in tumor necrosis factor alpha-induced activation of the matrix metalloproteinase/sphingolipid mitogenic pathway.. Mol Cell Biol.

[9] Marchesini N, Osta W, Bielawski J, Luberto C, Obeid LM (2004). Role for mammalian neutral sphingomyelinase 2 in confluence-induced growth arrest of MCF7 cells.. J Biol Chem.

[10] Kolesnick R, Hannun YA (1999). Ceramide and apoptosis.. Trends Biochem Sci.

[11] Tomiuk S, Hofmann K, Nix M, Zumbansen M, Stoffel W (1998). Cloned mammalian neutral sphingomyelinase: functions in sphingolipid signaling?. Proc Natl Acad Sci U S A.

[12] Zumbansen M, Stoffel W (2002). Neutral sphingomyelinase 1 deficiency in the mouse causes no lipid storage disease.. Mol Cell Biol.

[13] Yabu T, Imamura S, Yamashita M, Okazaki T (2008). Identification of Mg2+ -dependent neutral sphingomyelinase 1 as a mediator of heat stress-induced ceramide generation and apoptosis.. J Biol Chem.

[14] Krut O, Wiegmann K, Kashkar H, Yazdanpanah B, Kronke M (2006). Novel tumor necrosis factor-responsive mammalian neutral sphingomyelinase-3 is a C-tail-anchored protein.. J Biol Chem.

[15] Tomiuk S, Zumbansen M, Stoffel W (2000). Characterization and subcellular localization of murine and human magnesium-dependent neutral sphingomyelinase.. J Biol Chem.

[16] De Palma C, Meacci E, Perrotta C, Bruni P, Clementi E (2006). Endothelial nitric oxide synthase activation by tumor necrosis factor alpha through neutral sphingomyelinase 2, sphingosine kinase 1, and sphingosine 1 phosphate receptors: a novel pathway relevant to the pathophysiology of endothelium.. Arterioscler Thromb Vasc Biol.

[17] Tani M, Hannun YA (2007). Neutral sphingomyelinase 2 is palmitoylated on multiple cysteine residues. Role of palmitoylation in subcellular localization.. J Biol Chem.

[18] Zeng C, Lee JT, Chen H, Chen S, Hsu CY (2005). Amyloid-beta peptide enhances tumor necrosis factor-alpha-induced iNOS through neutral sphingomyelinase/ceramide pathway in oligodendrocytes.. J Neurochem.

[19] Aubin I, Adams CP, Opsahl S, Septier D, Bishop CE (2005). A deletion in the gene encoding sphingomyelin phosphodiesterase 3 (Smpd3) results in osteogenesis and dentinogenesis imperfecta in the mouse.. Nat Genet.

[20] Stoffel W, Jenke B, Block B, Zumbansen M, Koebke J (2005). Neutral sphingomyelinase 2 (smpd3) in the control of postnatal growth and development.. Proc Natl Acad Sci U S A.

[21] Wajant H (2003). Death receptors.. Essays Biochem.

[22] Ding WX, Yin XM (2004). Dissection of the multiple mechanisms of TNF-alpha-induced apoptosis in liver injury.. J Cell Mol Med.

[23] Chen G, Goeddel DV (2002). TNF-R1 signaling: a beautiful pathway.. Science.

[24] Auge N, Pieraggi MT, Thiers JC, Negre-Salvayre A, Salvayre R (1995). Proliferative and cytotoxic effects of mildly oxidized low-density lipoproteins on vascular smooth-muscle cells.. Biochem J.

[25] Kittrell FS, Oborn CJ, Medina D (1992). Development of mammary preneoplasias in vivo from mouse mammary epithelial cell lines in vitro.. Cancer Res.

[26] Auge N, Maupas-Schwalm F, Elbaz M, Thiers JC, Waysbort A (2004). Role for matrix metalloproteinase-2 in oxidized low-density lipoprotein-induced activation of the sphingomyelin/ceramide pathway and smooth muscle cell proliferation.. Circulation.

[27] Bielawski J, Szulc ZM, Hannun YA, Bielawska A (2006). Simultaneous quantitative analysis of bioactive sphingolipids by high-performance liquid chromatography-tandem mass spectrometry.. Methods.

[28] Vieira O, Escargueil-Blanc I, Jurgens G, Borner C, Almeida L (2000). Oxidized LDLs alter the activity of the ubiquitin-proteasome pathway: potential role in oxidized LDL-induced apoptosis.. FASEB J.

[29] Auge N, Garcia V, Maupas-Schwalm F, Levade T, Salvayre R (2002). Oxidized LDL-induced smooth muscle cell proliferation involves the EGF receptor/PI-3 kinase/Akt and the sphingolipid signaling pathways.. Arterioscler Thromb Vasc Biol.

[30] Brouckaert P, Libert C, Everaerdt B, Takahashi N, Cauwels A (1993). Tumor necrosis factor, its receptors and the connection with interleukin 1 and interleukin 6.. Immunobiology.

[31] Hofmann K, Tomiuk S, Wolff G, Stoffel W (2000). Cloning and characterization of the mammalian brain-specific, Mg2+-dependent neutral sphingomyelinase.. Proc Natl Acad Sci U S A.

[32] Wilson NS, Dixit V, Ashkenazi A (2009). Death receptor signal transducers: nodes of coordination in immune signaling networks.. Nat Immunol.

[33] Hofmann K, Dixit VM (1998). Ceramide in apoptosis–does it really matter?. Trends Biochem Sci.

[34] Carpinteiro A, Dumitru C, Schenck M, Gulbins E (2008). Ceramide-induced cell death in malignant cells.. Cancer Lett.

[35] Smith EL, Schuchman EH (2008). The unexpected role of acid sphingomyelinase in cell death and the pathophysiology of common diseases.. FASEB J.

[36] Clarke CJ, Truong TG, Hannun YA (2007). Role for neutral sphingomyelinase-2 in tumor necrosis factor alpha-stimulated expression of vascular cell adhesion molecule-1 (VCAM) and intercellular adhesion molecule-1 (ICAM) in lung epithelial cells: p38 MAPK is an upstream regulator of nSMase2.. J Biol Chem.

[37] Kim WJ, Okimoto RA, Purton LE, Goodwin M, Haserlat SM (2008). Mutations in the neutral sphingomyelinase gene SMPD3 implicate the ceramide pathway in human leukemias.. Blood.

[38] Escargueil-Blanc I, Andrieu-Abadie N, Caspar-Bauguil S, Brossmer R, Levade T (1998). Apoptosis and activation of the sphingomyelin-ceramide pathway induced by oxidized low density lipoproteins are not causally related in ECV-304 endothelial cells.. J Biol Chem.

[39] Escargueil-Blanc I, Salvayre R, Negre-Salvayre A (1994). Necrosis and apoptosis induced by oxidized low density lipoproteins occur through two calcium-dependent pathways in lymphoblastoid cells.. FASEB J.

[40] Porn-Ares MI, Saido TC, Andersson T, Ares MP (2003). Oxidized low-density lipoprotein induces calpain-dependent cell death and ubiquitination of caspase 3 in HMEC-1 endothelial cells.. Biochem J.

[41] Vindis C, Elbaz M, Escargueil-Blanc I, Auge N, Heniquez A (2005). Two distinct calcium-dependent mitochondrial pathways are involved in oxidized LDL-induced apoptosis.. Arterioscler Thromb Vasc Biol.

[42] Grassme H, Riethmuller J, Gulbins E (2007). Biological aspects of ceramide-enriched membrane domains.. Prog Lipid Res.

[43] Garcia-Ruiz C, Colell A, Mari M, Morales A, Calvo M (2003). Defective TNF-alpha-mediated hepatocellular apoptosis and liver damage in acidic sphingomyelinase knockout mice.. J Clin Invest.

[44] Osawa M, Dace A, Tong KI, Valiveti A, Ikura M (2005). Mg2+ and Ca2+ differentially regulate DNA binding and dimerization of DREAM.. J Biol Chem.

