# Expression of Concern: Stress-Induced Sphingolipid Signaling: Role of Type-2 Neutral Sphingomyelinase in Murine Cell Apoptosis and Proliferation

**DOI:** 10.1371/journal.pone.0208866

**Published:** 2018-12-10

**Authors:** The PLOS ONE Editors

Concerns have been raised regarding partial duplication of some images in this article [1]. Specifically, some individual cells and cell clusters from the bottom left panel of Fig 3E (wt + stauro) appear to be present within a different context in the middle and bottom panels of Fig 1C (TNF-α and ox LDL).

**Fig 1:**
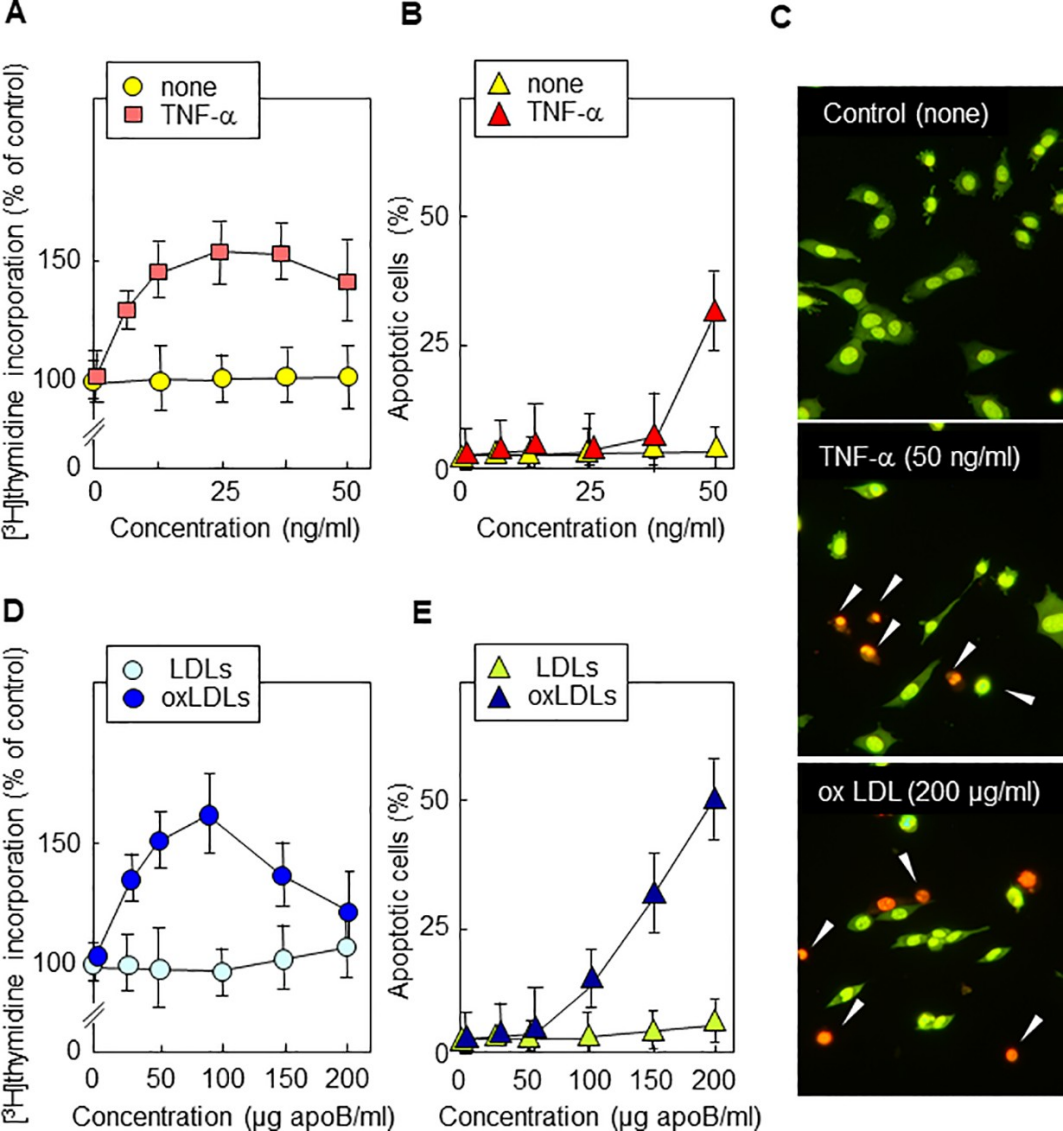
TNFα and oxLDLs induce dose-dependent apoptotic or mitogenic effects in wt murine fibroblasts. Wild-type (wt) murine fibroblasts were starved in serum-free medium for 24 h, then were treated for 24 h with the indicated concentration of TNFα (0–50 ng/ml) or of LDL non oxidized (LDLs) and oxidized (oxLDLs) (0–200 μg/ml). DNA synthesis was quantified by [^3^H]thymidine incorporation and was expressed as % of untreated control (A,D). Apoptosis and necrosis were visualized by fluorescence microscopy of cells stained by Syto13/PI (B,C,E). Primary apoptosis was characterized by condensed picnotic or fragmented nuclear stained green/yellow by Syto13, whereas post-apoptotic necrosis exhibited the same nuclear morphology but was stained red by PI (permeabilization of the plasma membrane in a late step of apoptosis). Only few cells exhibited the feature of primary necrosis, i.e. loose chromatin stained red. In, A,B,D,E, Mean ± SEM of 4 experiments. *: p<0.05.

**Fig 3:**
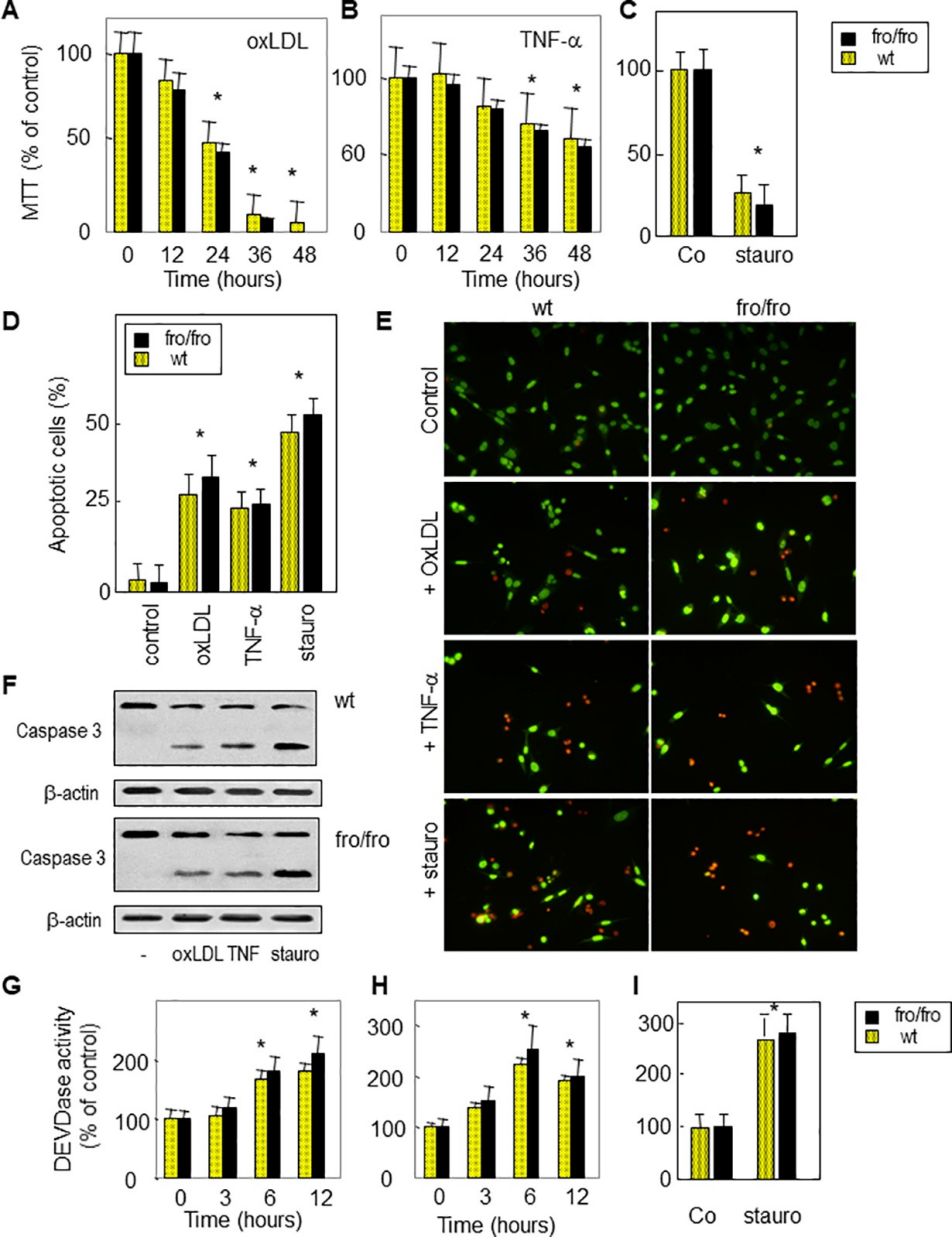
TNFα, oxLDLs and staurosporine induce cell apoptosis in wt and *fro/fro*fibroblasts. Fibroblasts were incubated with TNFα (50 ng/ml, 48 h), oxLDLs (200 μg/ml, 24 h) or staurosporine (100 nM, 6 h). Cell viability was evaluated by the MTT assay (A–C) and by counting apoptotic cells after syto13/PI labeling (D,E), as in Fig 1. Caspase 3 activation was determined by western blot showing pro-caspase (32 kDa) and cleaved active caspase (17 kDa) (F). Time-course of DEVDase activity were measured using the fluorogenic substrate Ac-DEVD-AMC in cells treated by oxLDLs, TNFα and staurosporin, respectively (G–I). The results are mean ± SEM of 3 to 5 separated experiments. * p<0.05 for apoptotic cell counting and DEVDase activity measurement (comparison between cells treated with or without agonist).

The corresponding author stands by the accuracy of the results presented in Fig 3E and has provided the original uncropped image file underlying the bottom left panel of Fig 3E. The corresponding author acknowledges that there appear to be abnormalities in Fig 1C with the appearance of a fusion or image overlay in the bottom panel, but states that the image alterations were not intentional, and it is not known how they occurred. The original image files for Fig 1C are unavailable. The corresponding author states that the conclusions are supported by more recent experimental replications (provided here as Supporting Information) and also by other methods (flow cytometry, as shown in supplemental S1 Fig of the original article).

Replacement Figs 1 and 3 are provided in which the panels containing abnormalities and those for which the original raw image files are no longer available (all panels of Fig 1C, Fig 3E wt control, *fro* +oxLDL, *fro* +TNF-α and *fro* +stauro) are replaced with replicates collected at the time of the original study. All original image files underlying these replacement figures are provided as Supporting Information.

An investigation conducted by the Research Integrity Office of INSERM confirmed that some original images from these experiments are no longer available, and the laboratory computer was unsecure at the time of the original study. An image in Fig 1C has been manipulated; however, the manipulated photo illustrates a control condition in an experiment which has been shown to be reproducible. A formal Correction was recommended to address the concerns.

The corresponding author has further clarified that the majority of the original data files underlying the rest of the article are also unavailable due to the fact that the study was completed more than 10 years ago; files have been lost or corrupted and some of the original investigators have left the institution. However, the corresponding author states that all the materials (cells, mice, agents) are available in their laboratory for investigators interested in replicating this work.

The *PLOS ONE* Editors have been unable to determine how the middle and bottom panels of Fig 1C were generated. Following consultation with a member of the editorial board, we understand that the replacement images support the conclusions. However, in light of the extent of the unavailability of data underlying this article and the image manipulation in Fig 1C, the *PLOS ONE* Editors issue this Expression of Concern.

## Supporting information

S1 File Corrected Fig 1 raw images. (ZIP)

S2 File Corrected Fig 3 raw images. (ZIP)

S3 File 2018 replication data (1 of 4). (ZIP)

S4 File 2018 replication data (2 of 4). (ZIP)

S5 File 2018 replication data (3 of 4). (ZIP)

S6 File 2018 replication data (4 of 4). (ZIP)

## References

[1] Devillard R, Galvani S, Thiers J-C, Guenet J-L, Hannun Y, Bielawski J, et al (2010) Stress-Induced Sphingolipid Signaling: Role of Type-2 Neutral Sphingomyelinase in Murine Cell Apoptosis and Proliferation. PLoS ONE 5(3): e9826 10.1371/journal.pone.0009826 20352118 PMC2843740

