# Supplementary figures and images for: Stress-Induced Sphingolipid Signaling: Role of Type-2 Neutral Sphingomyelinase in Murine Cell Apoptosis and Proliferation
Source: PLoS One. 2010 Mar 23;5(3):e9826. doi: 10.1371/journal.pone.0009826 (PMC2843740; doi:10.1371/journal.pone.0009826)

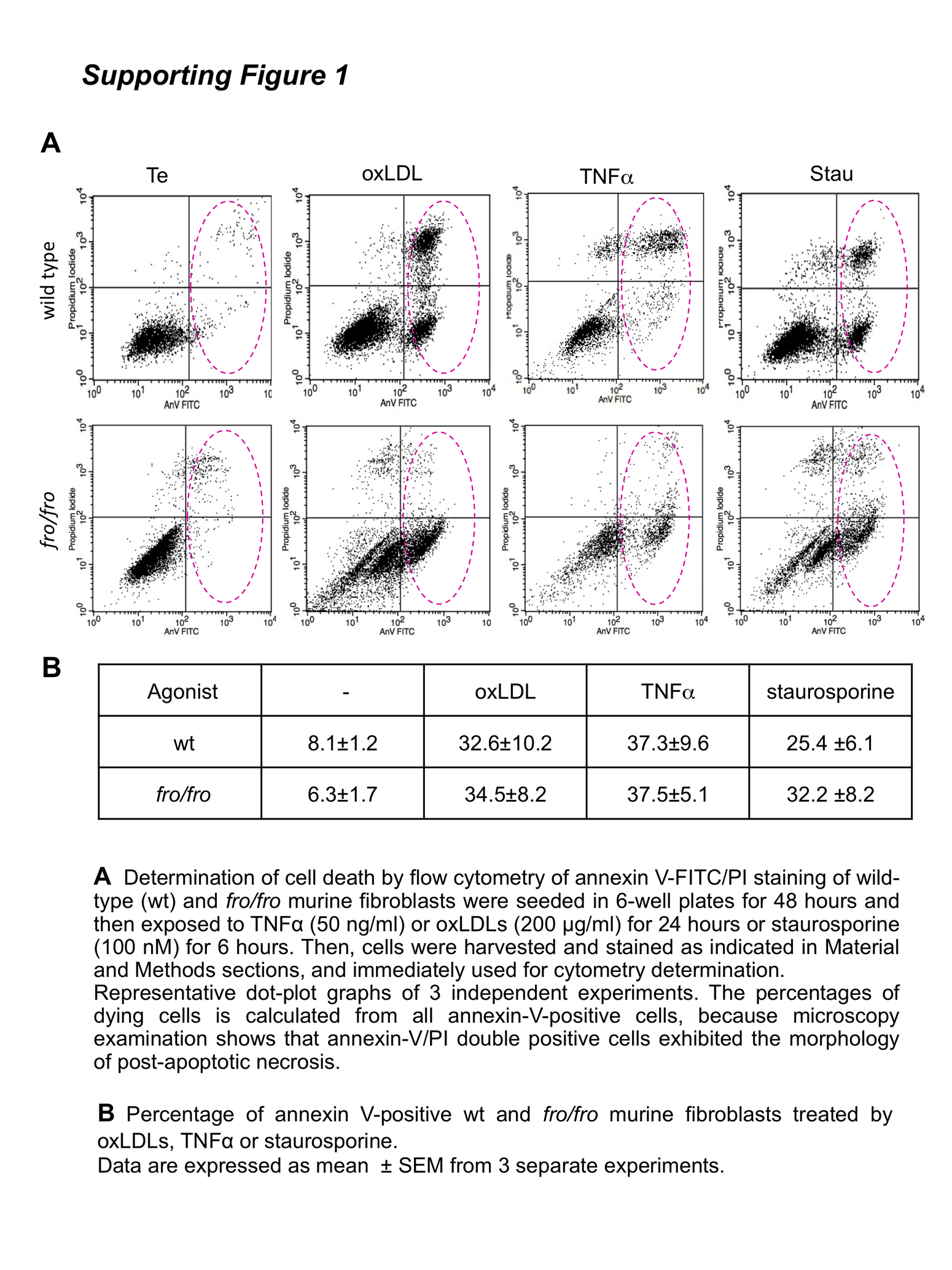

Supplement: Figure S1 — Flow cytometry. A Determination of cell death by flow cytometry of annexin V-FITC/PI staining of wild-type (wt) and fro/fro murine fibroblasts were seeded in 6-well plates for 48 hours and then exposed to TNFα (50 ng/ml) or oxLDLs (200 µg/ml) for 24 hours or staurosporine (100 nM) for 6 hours. Then, cells were harvested and stained as indicated in Material and Methods section, and immediately used for cytometry determination. Representative dot-plot graphs of 3 independent experiments. The percentages of dying cells is calculated from all annexin-V-positive cells, because microscopy examination shows that annexin-V/PI double positive cells exhibited the morphology of post-apoptotic necrosis. B Percentage of annexin V-positive wt and fro/fro murine fibroblasts treated by oxLDLs, TNFα or staurosporine. Data are expressed as mean ± SEM from 3 separate experiments. (1.21 MB TIF) [file pone.0009826.s001.tif]
