# Supplementary figures and images for: Expression of Concern: Stress-Induced Sphingolipid Signaling: Role of Type-2 Neutral Sphingomyelinase in Murine Cell Apoptosis and Proliferation
Source: PLoS One. 2018 Dec 10;13(12):e0208866. doi: 10.1371/journal.pone.0208866 (PMC6287835; doi:10.1371/journal.pone.0208866)

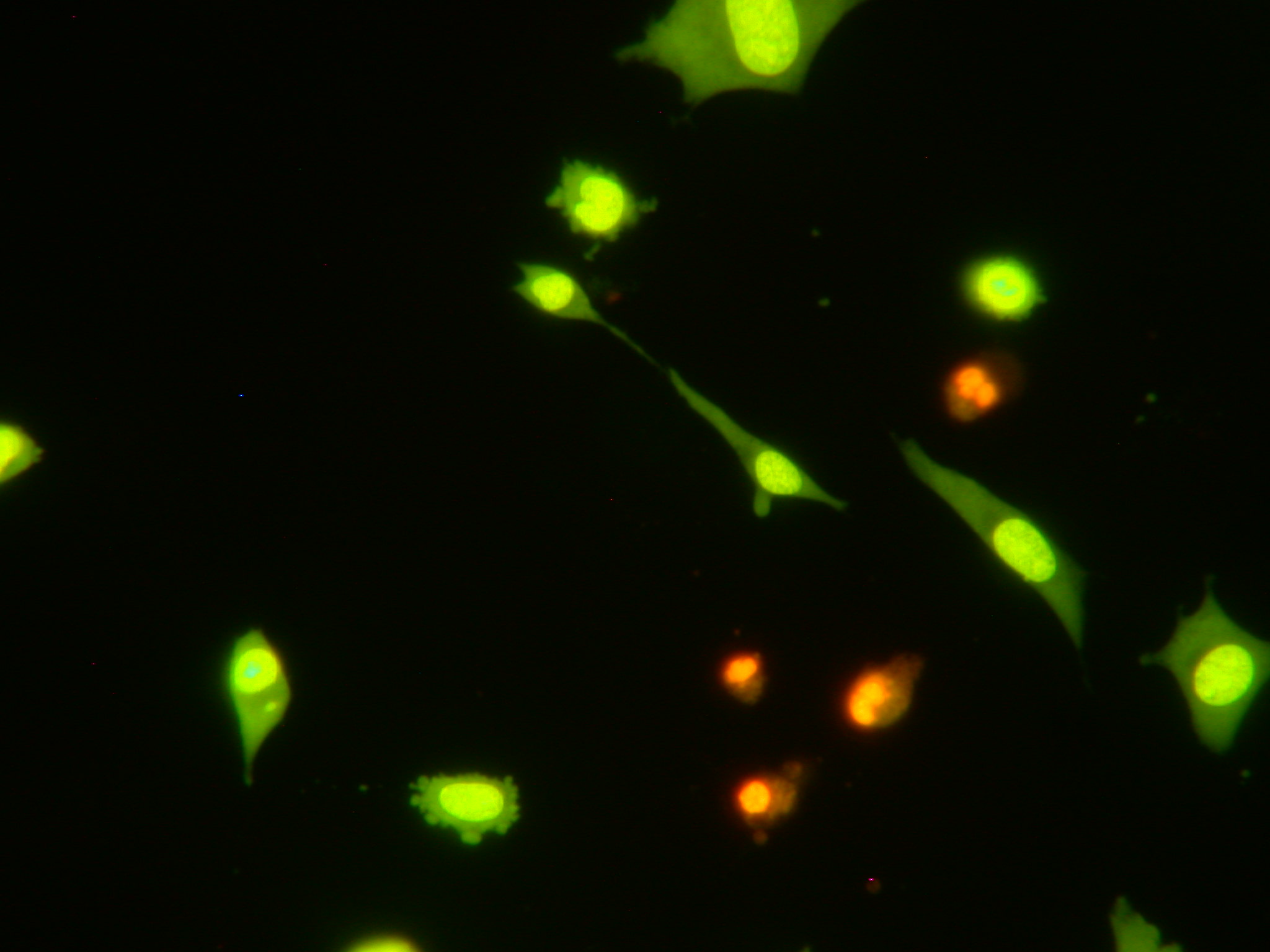

Supplement: S1 File — (ZIP) [file pone.0208866.s001.zip › Corrected Fig 1 raw images/DSCN1572.JPG]

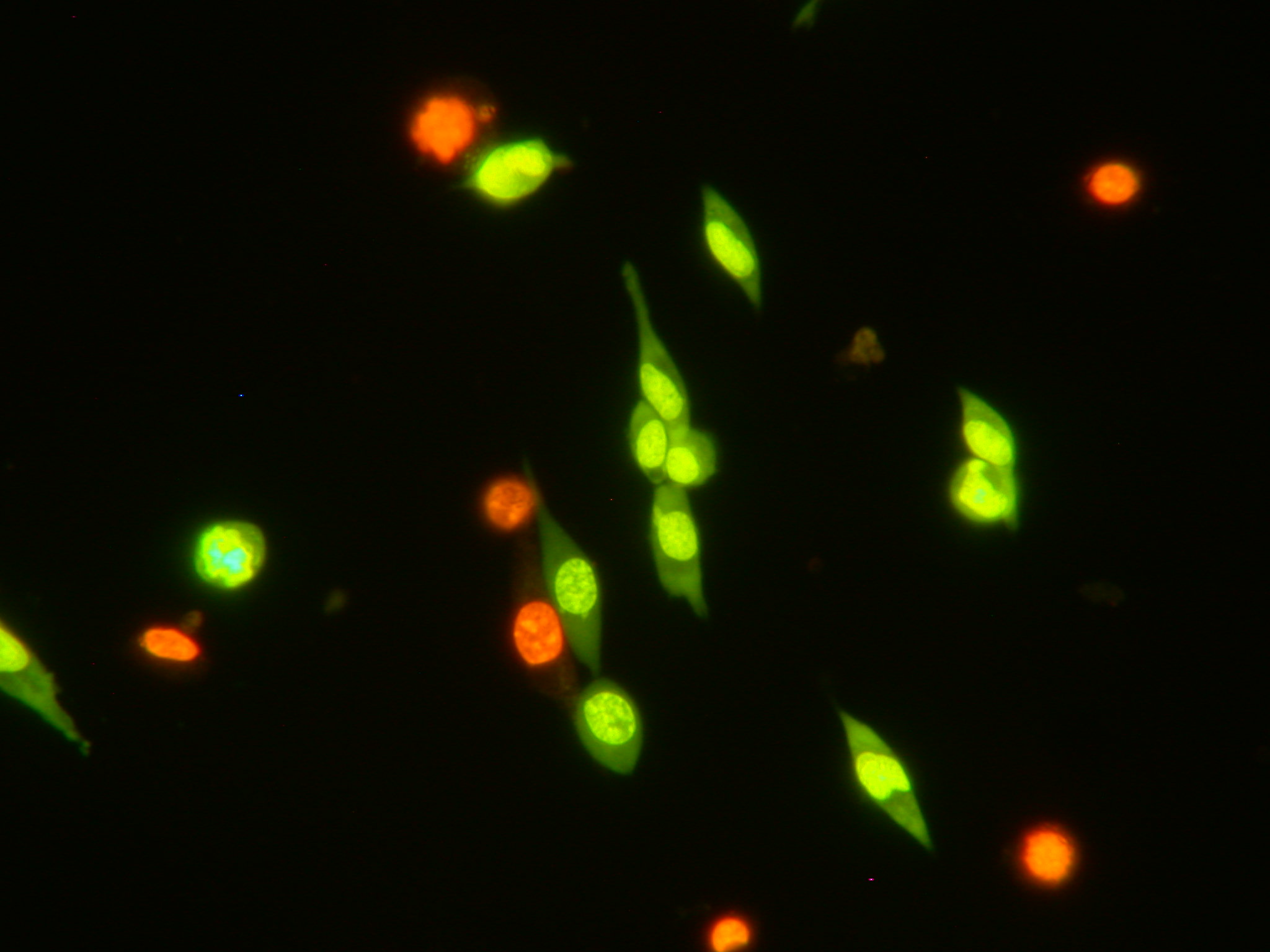

Supplement: S1 File — (ZIP) [file pone.0208866.s001.zip › Corrected Fig 1 raw images/DSCN1569.JPG]

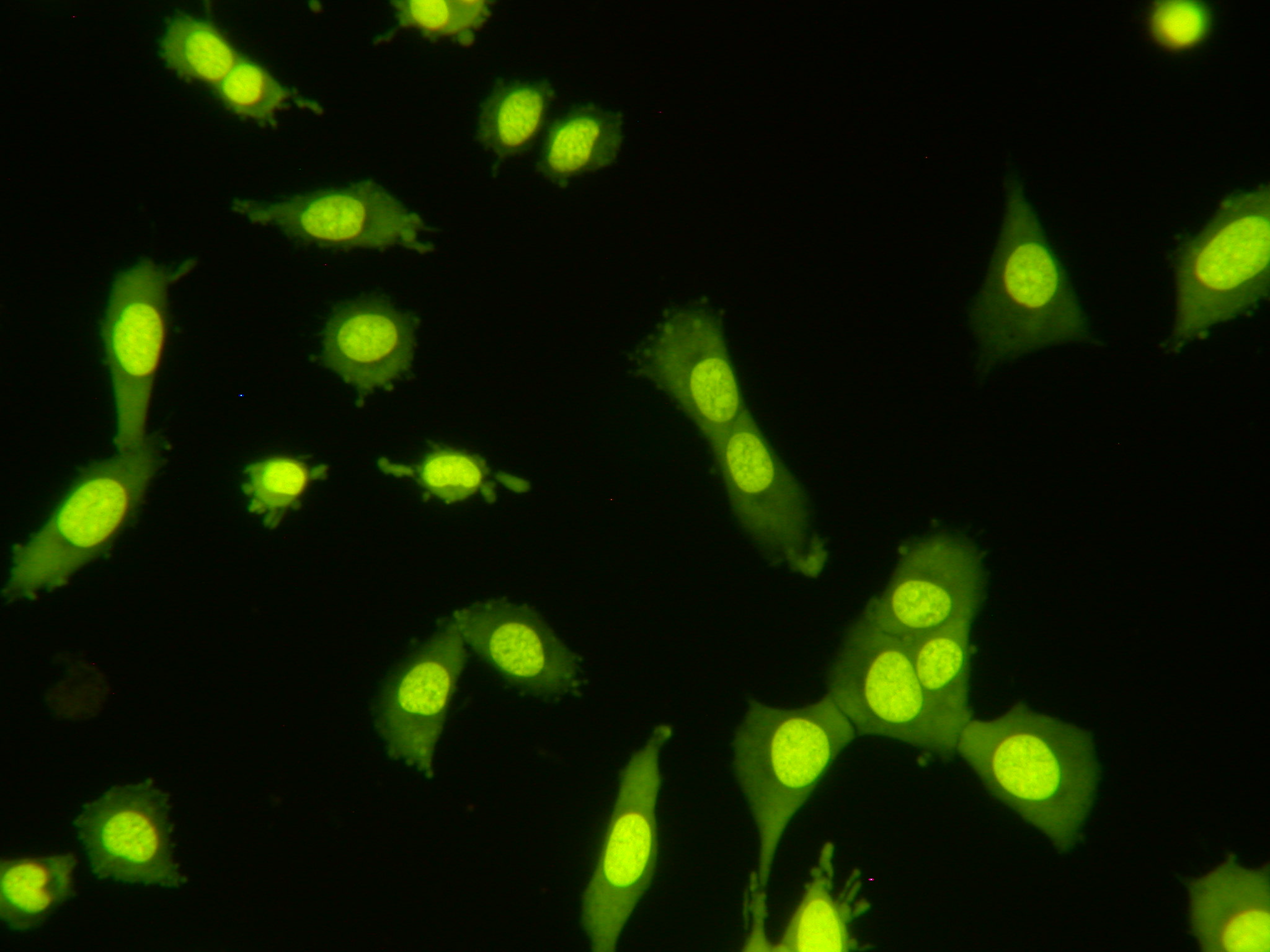

Supplement: S1 File — (ZIP) [file pone.0208866.s001.zip › Corrected Fig 1 raw images/DSCN1566.JPG]

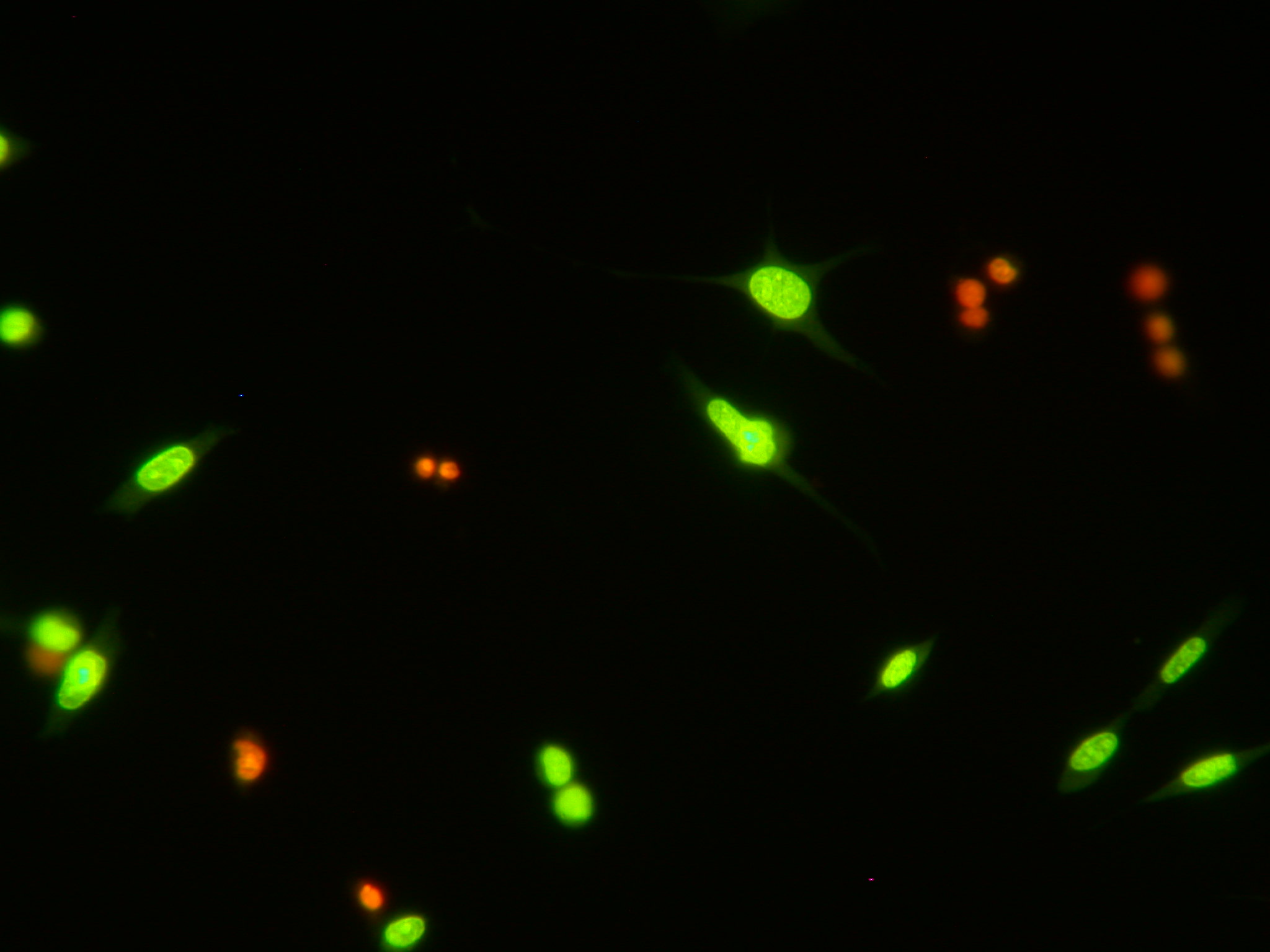

Supplement: S2 File — (ZIP) [file pone.0208866.s002.zip › Fig 3 raw images/replacement images for missing image files Fig 3/DSCN9698 fro tnf.JPG]

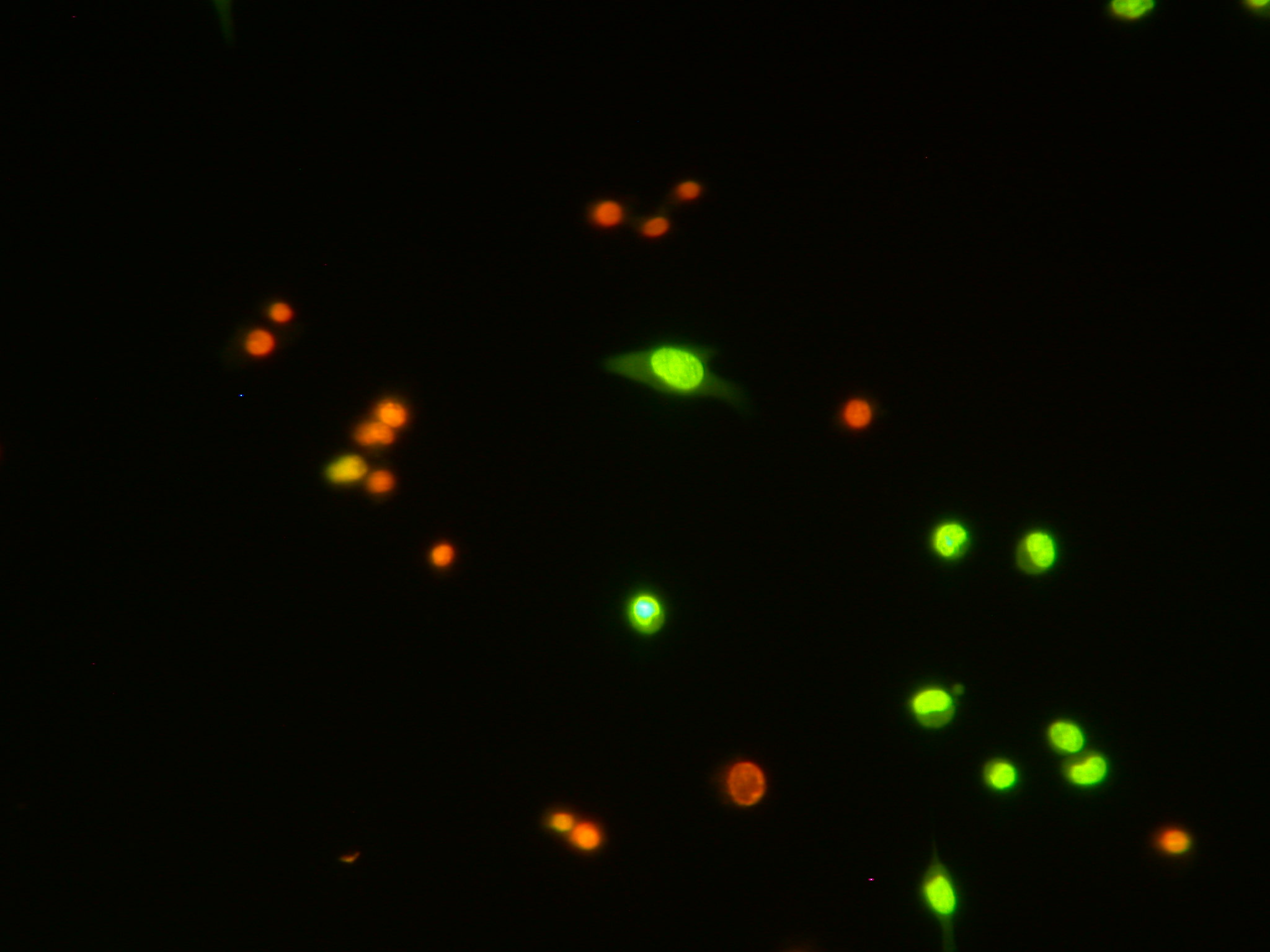

Supplement: S2 File — (ZIP) [file pone.0208866.s002.zip › Fig 3 raw images/replacement images for missing image files Fig 3/DSCN9692 fro stau.JPG]

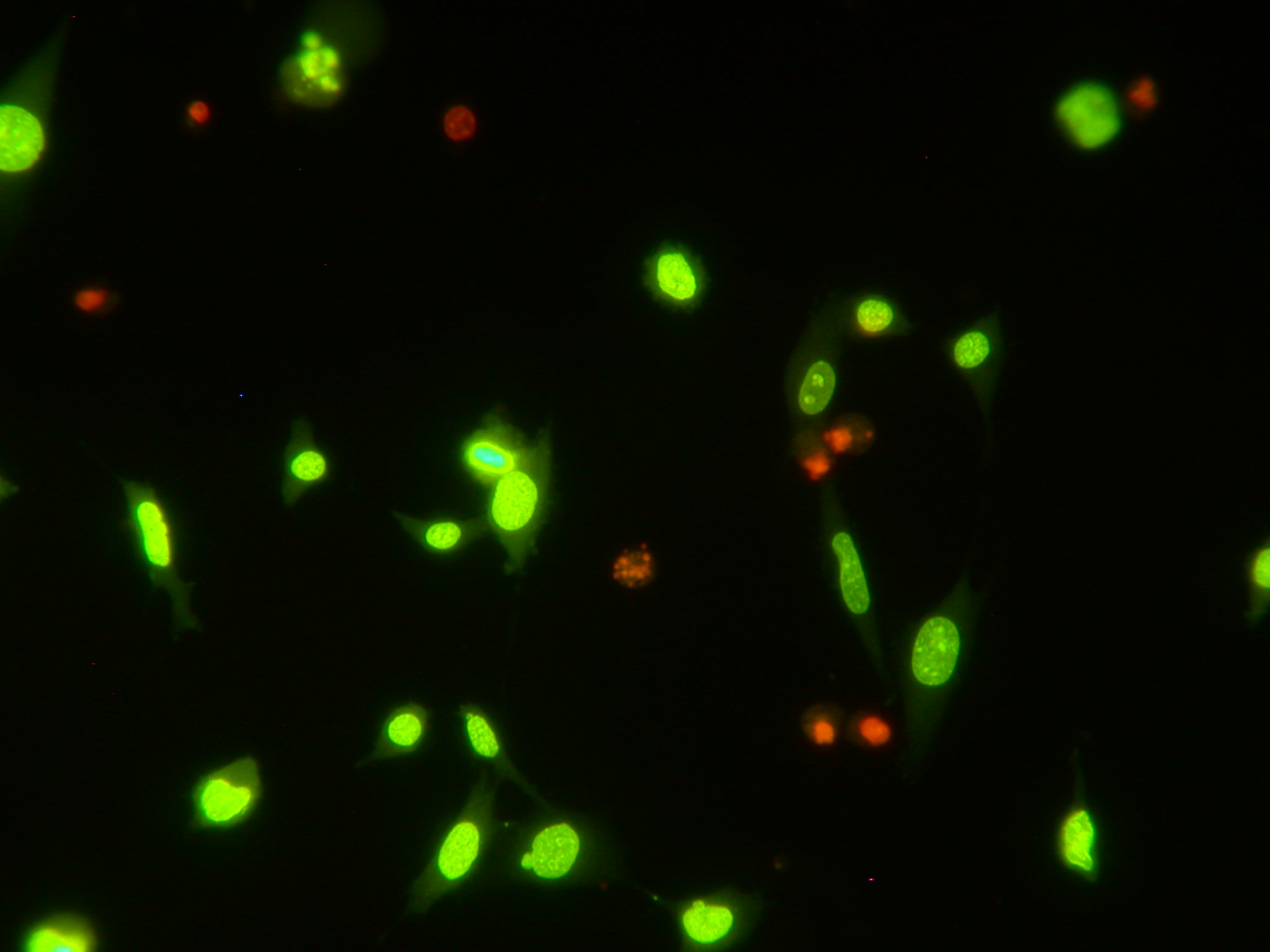

Supplement: S2 File — (ZIP) [file pone.0208866.s002.zip › Fig 3 raw images/replacement images for missing image files Fig 3/DSCN9688fro oxLDL.JPG]

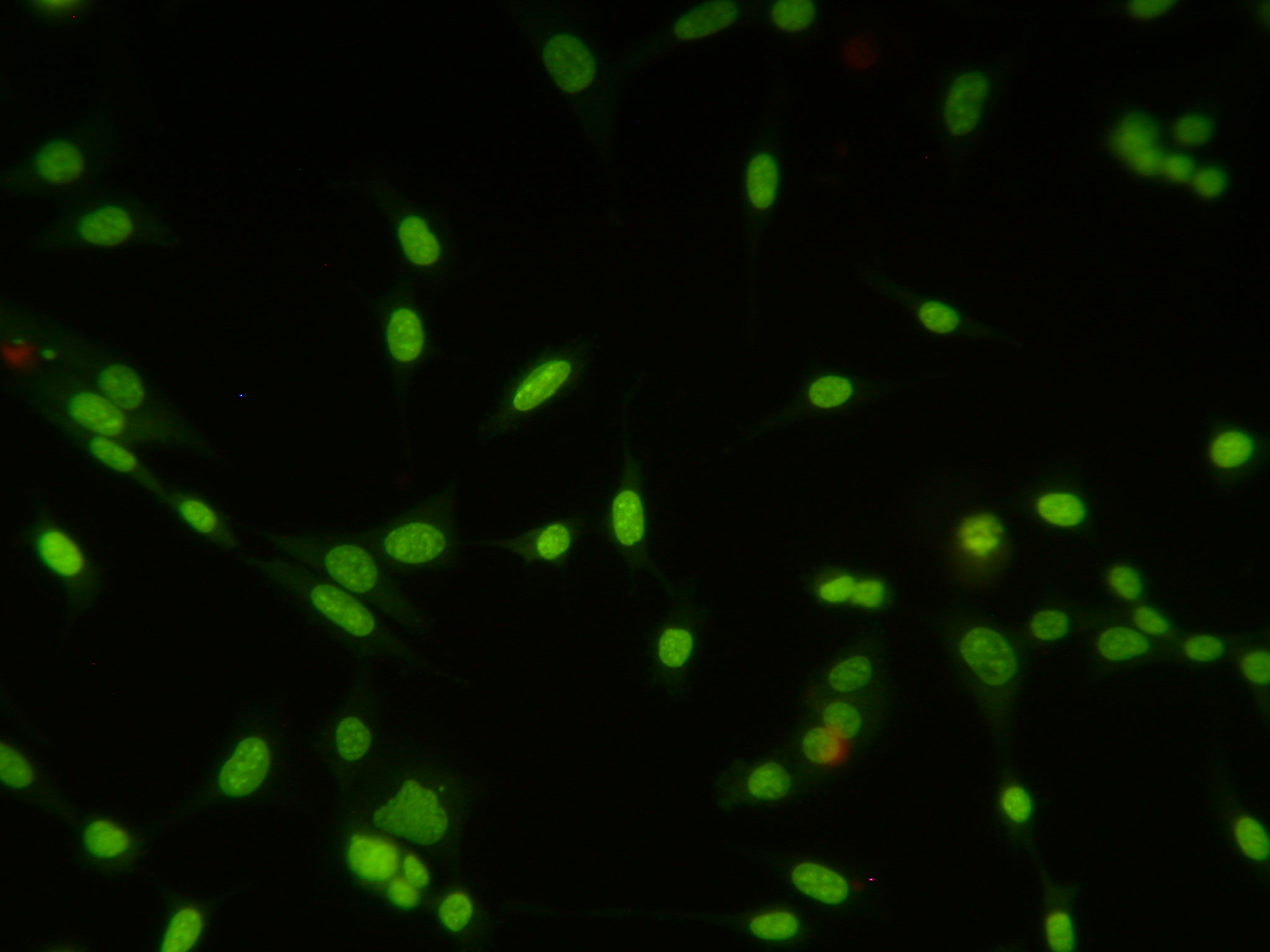

Supplement: S2 File — (ZIP) [file pone.0208866.s002.zip › Fig 3 raw images/replacement images for missing image files Fig 3/DSCN9701 wt cntrl.JPG]

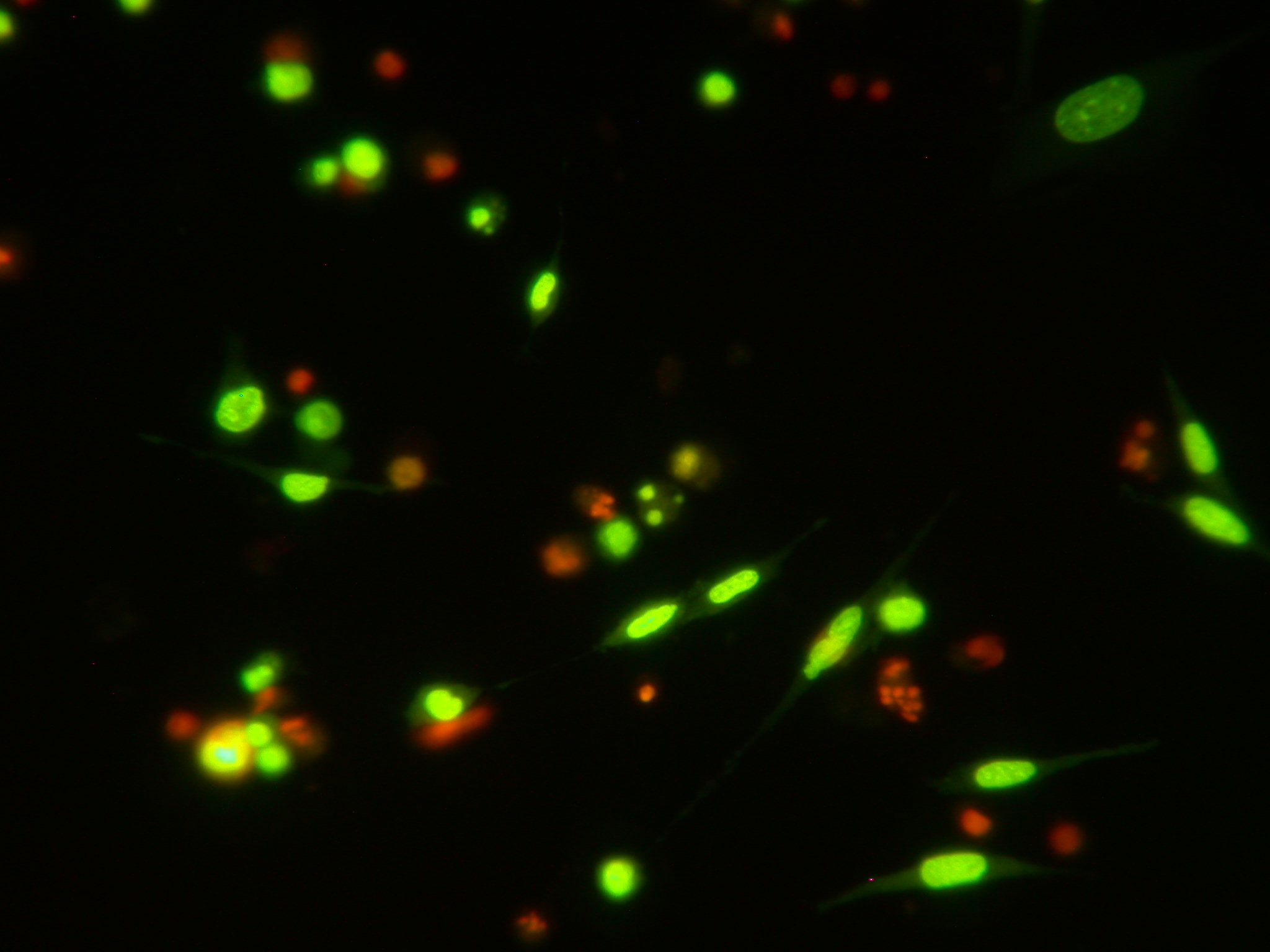

Supplement: S2 File — (ZIP) [file pone.0208866.s002.zip › Fig 3 raw images/present in original Fig 3/DSCN9710.JPG]

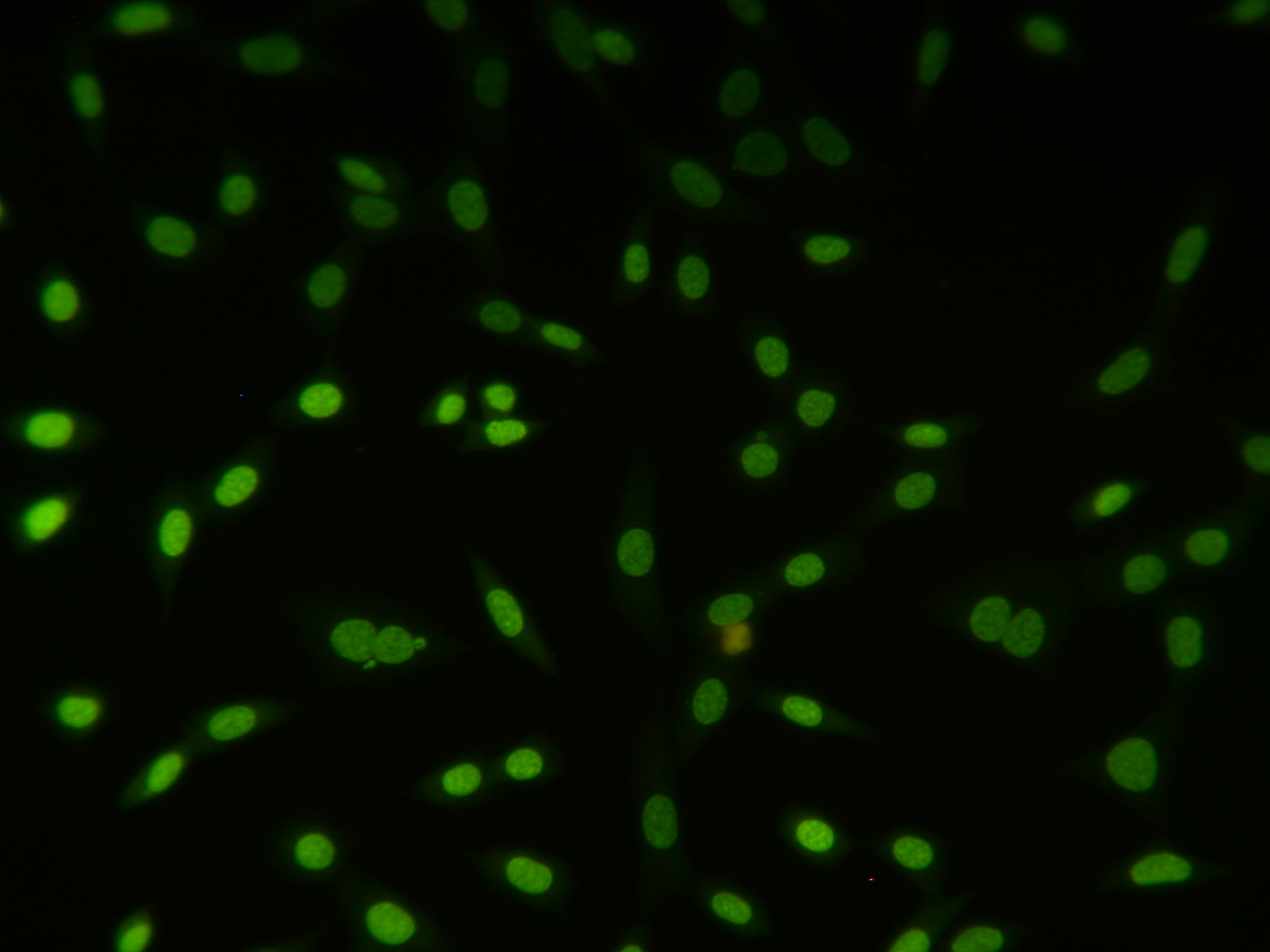

Supplement: S2 File — (ZIP) [file pone.0208866.s002.zip › Fig 3 raw images/present in original Fig 3/DSCN9683 fro cntrl.JPG]

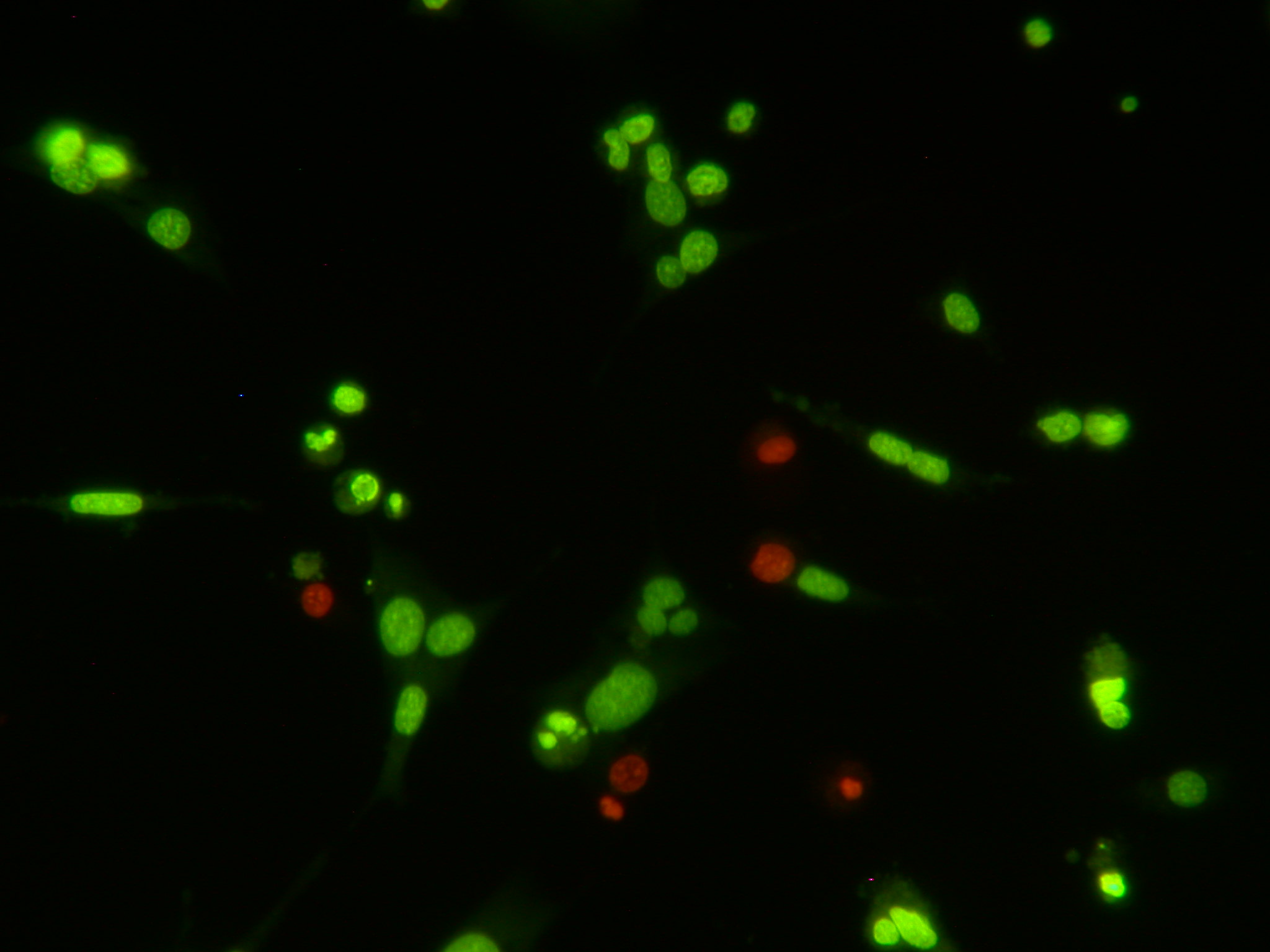

Supplement: S2 File — (ZIP) [file pone.0208866.s002.zip › Fig 3 raw images/present in original Fig 3/DSCN9738.JPG]

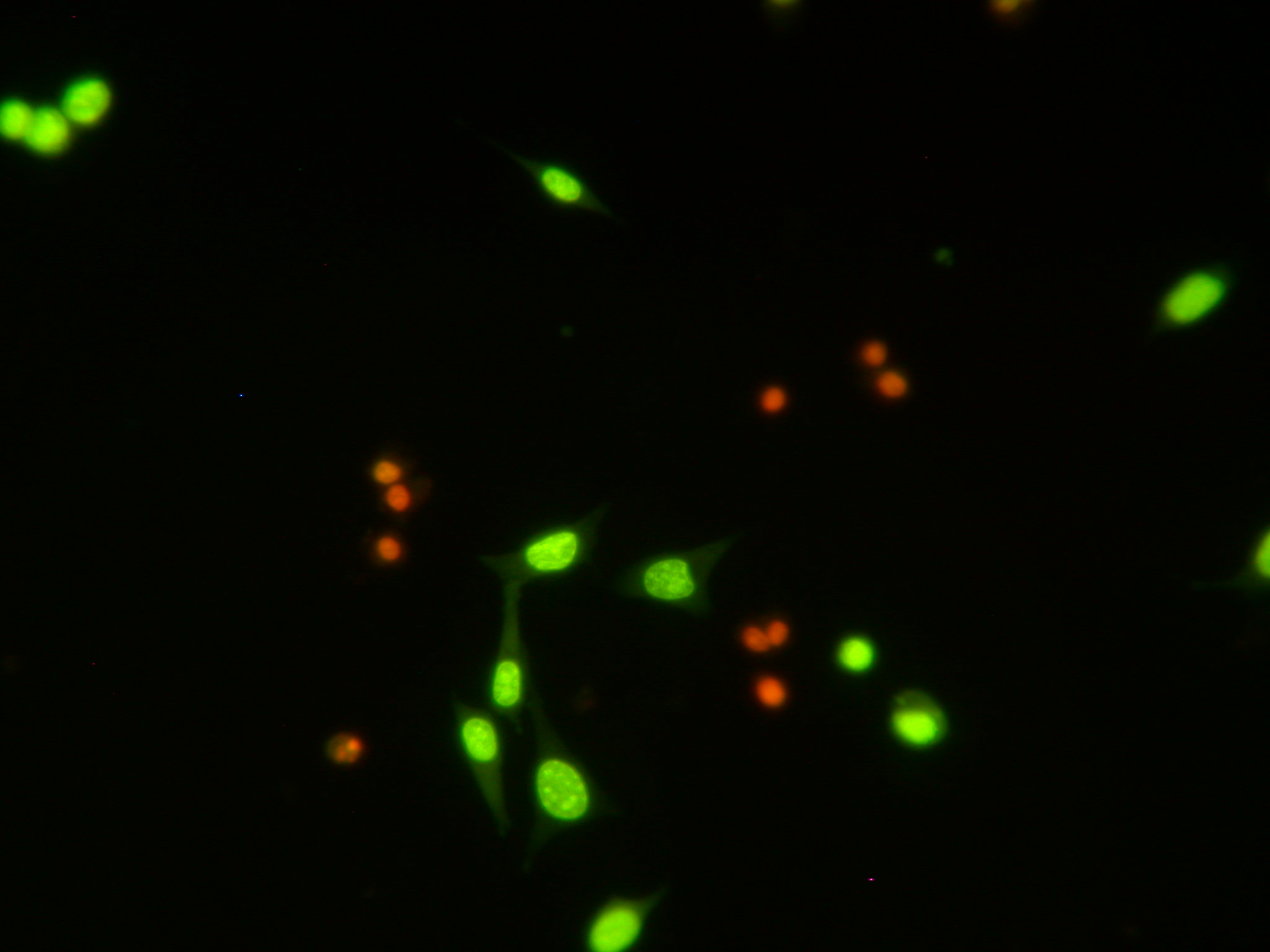

Supplement: S2 File — (ZIP) [file pone.0208866.s002.zip › Fig 3 raw images/present in original Fig 3/DSCN9699.JPG]

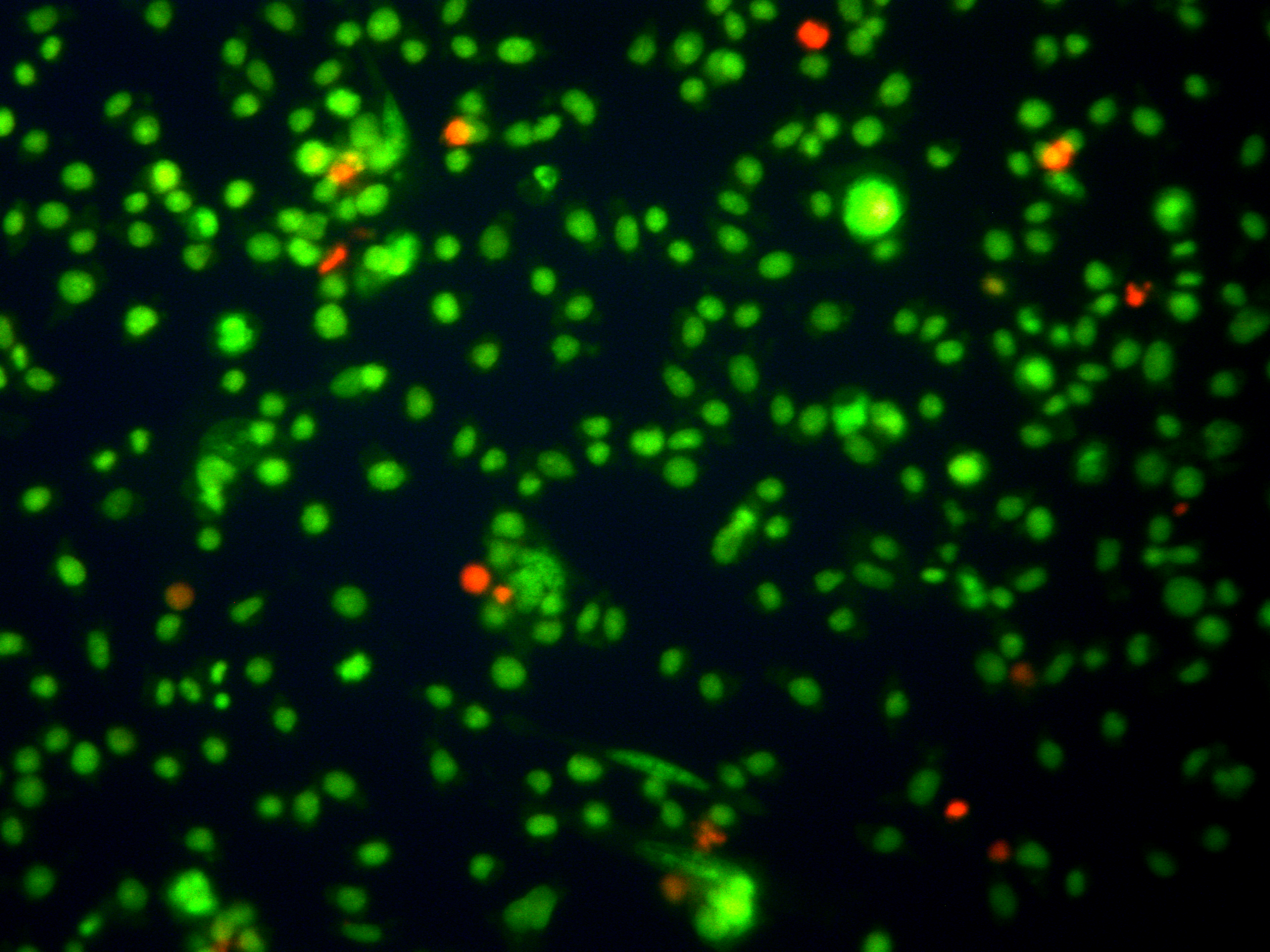

Supplement: S3 File — (ZIP) [file pone.0208866.s003.zip › S3_File/pone.0009826 EOC Replication Data 2018 (1 of 4)/cnt.tif]

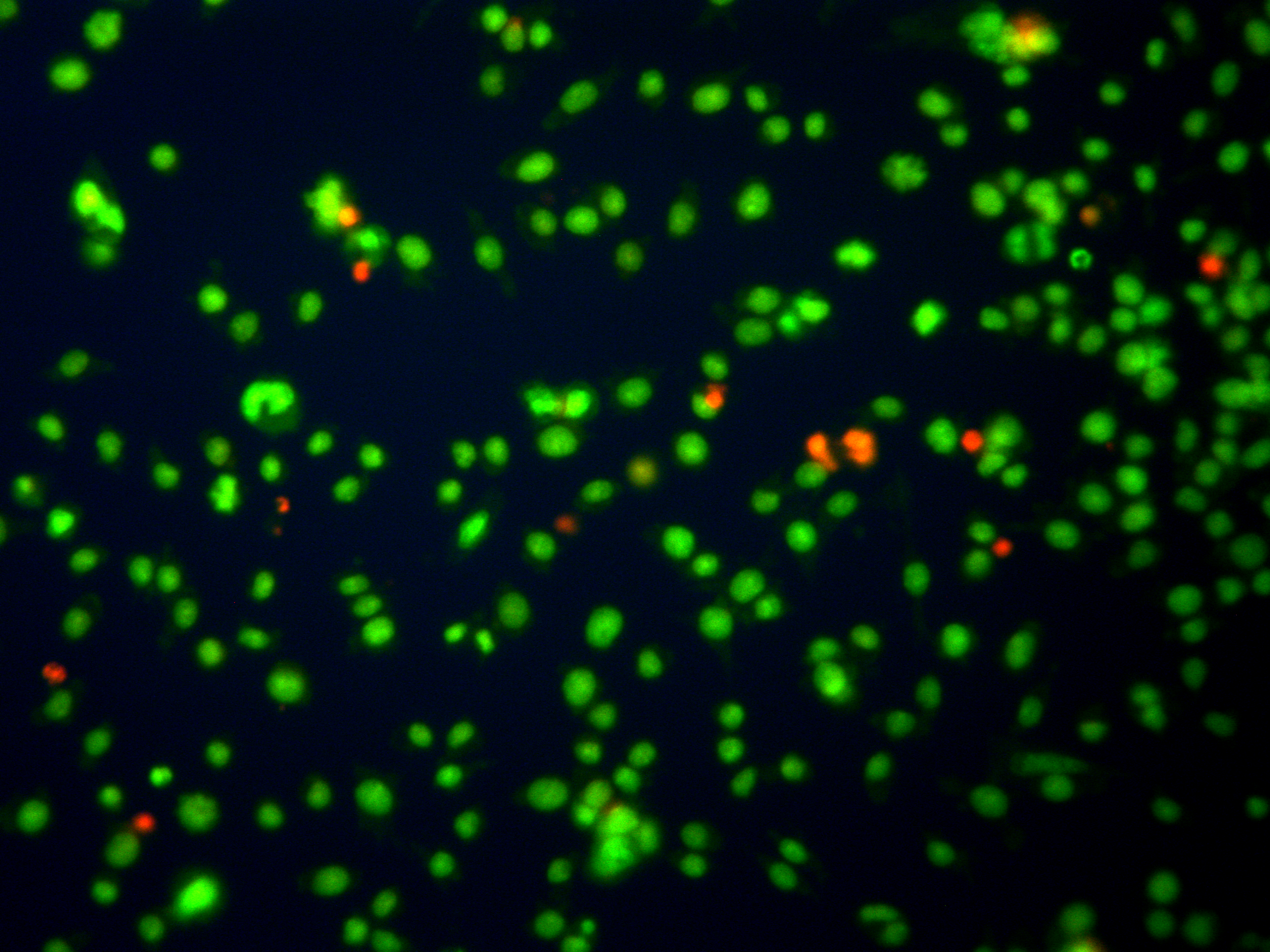

Supplement: S3 File — (ZIP) [file pone.0208866.s003.zip › S3_File/pone.0009826 EOC Replication Data 2018 (1 of 4)/cnt0001.tif]

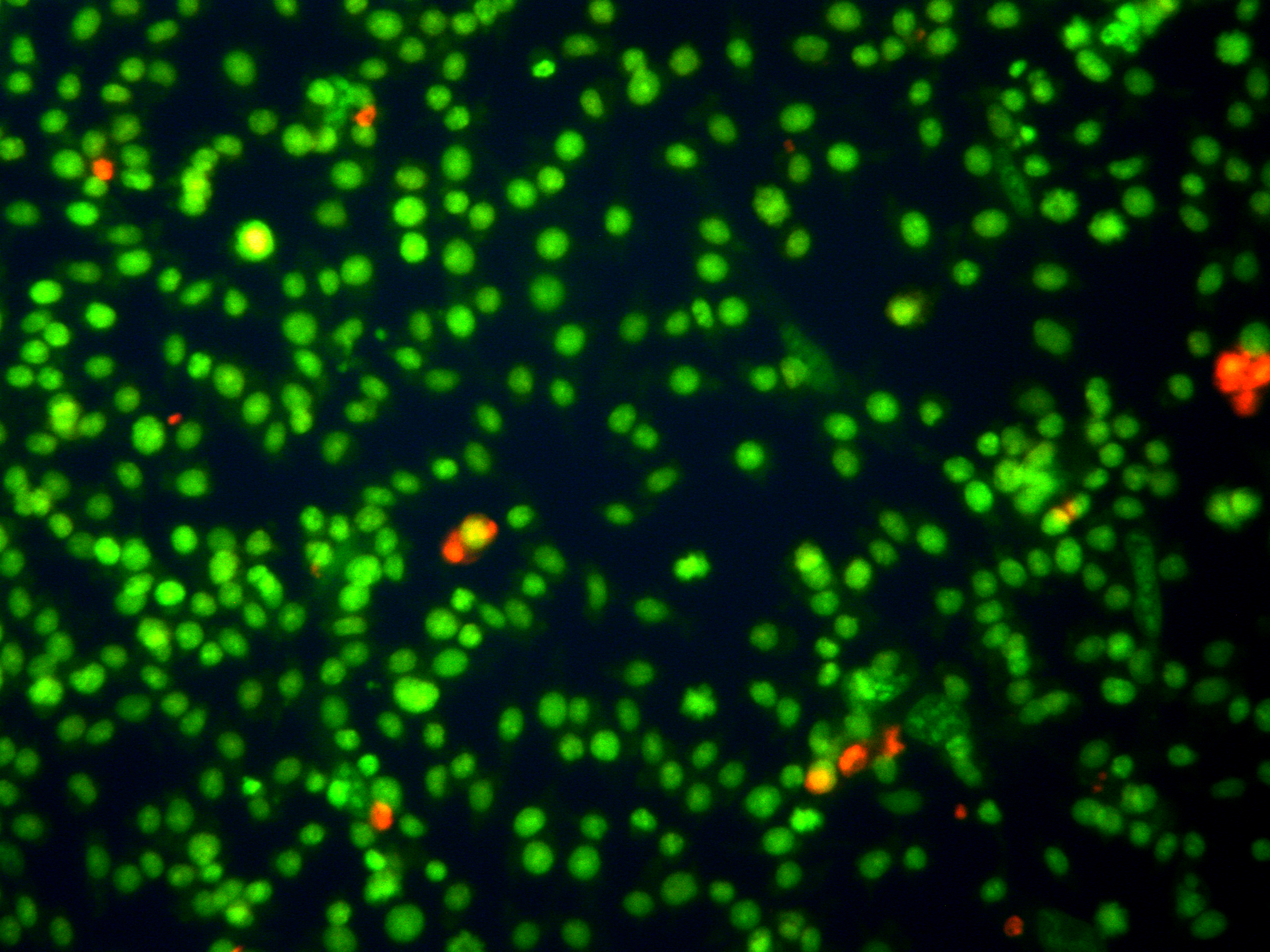

Supplement: S3 File — (ZIP) [file pone.0208866.s003.zip › S3_File/pone.0009826 EOC Replication Data 2018 (1 of 4)/cnt0002.tif]

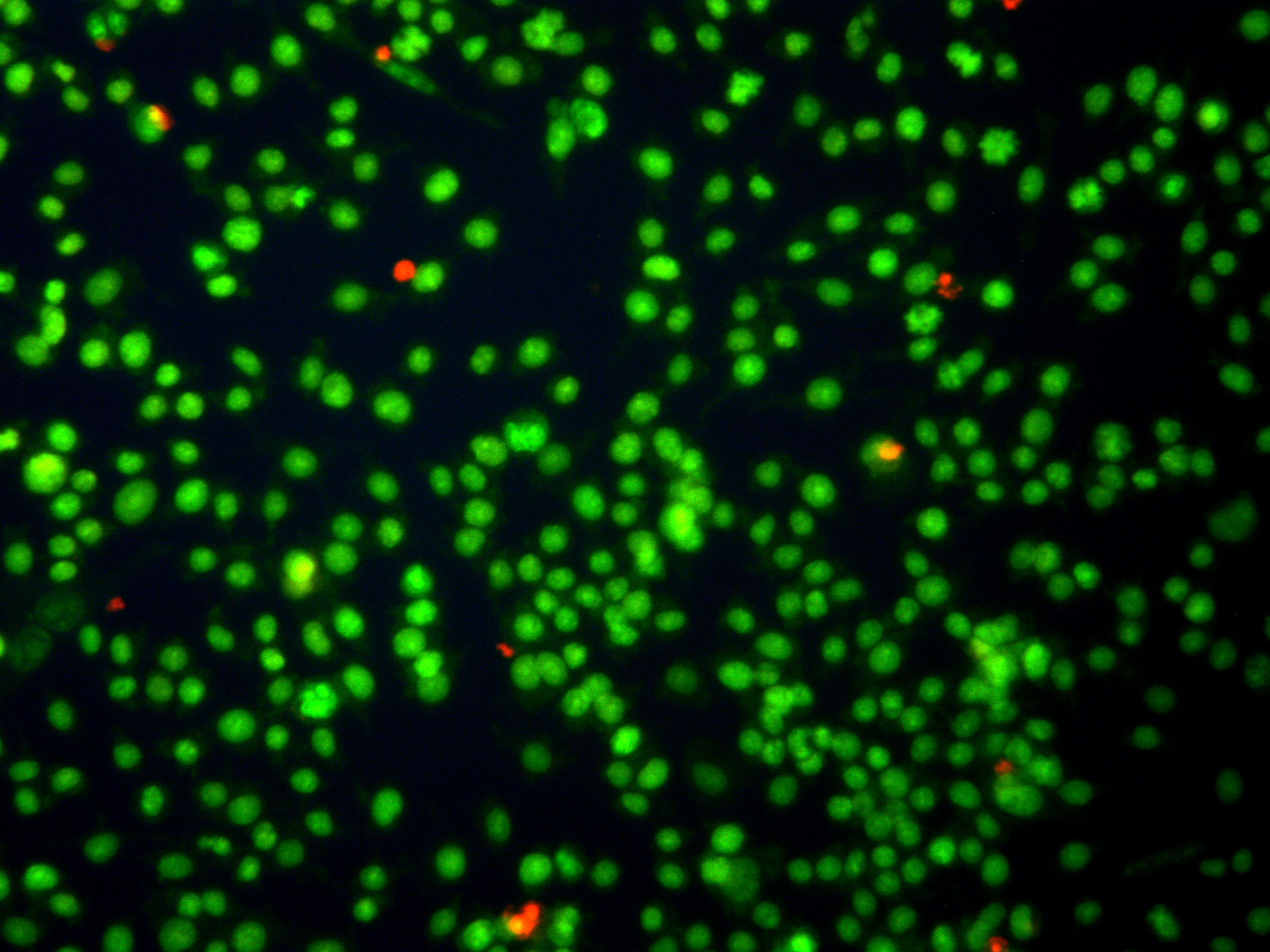

Supplement: S3 File — (ZIP) [file pone.0208866.s003.zip › S3_File/pone.0009826 EOC Replication Data 2018 (1 of 4)/cnt0003.tif]

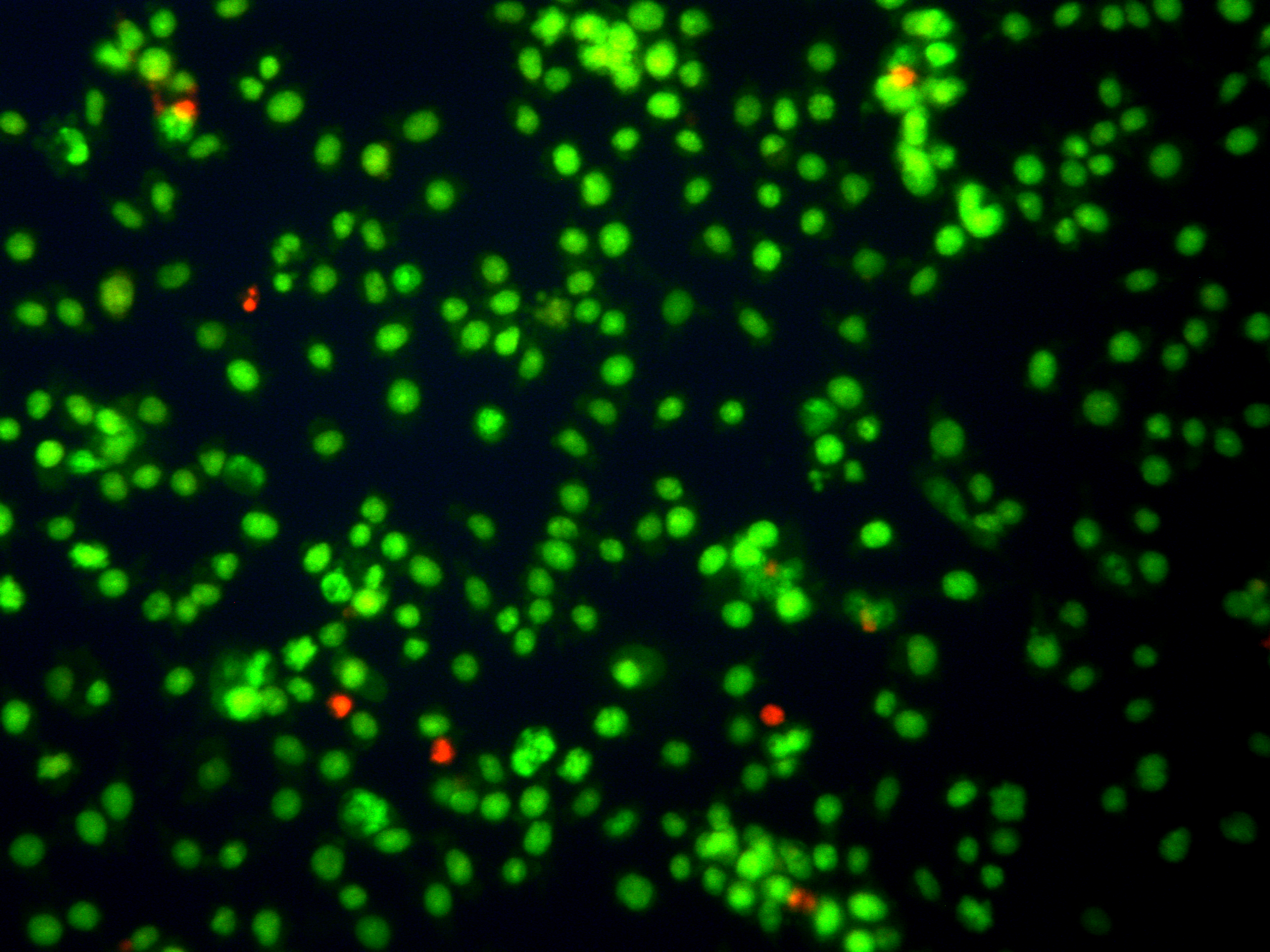

Supplement: S3 File — (ZIP) [file pone.0208866.s003.zip › S3_File/pone.0009826 EOC Replication Data 2018 (1 of 4)/cnt0004.tif]

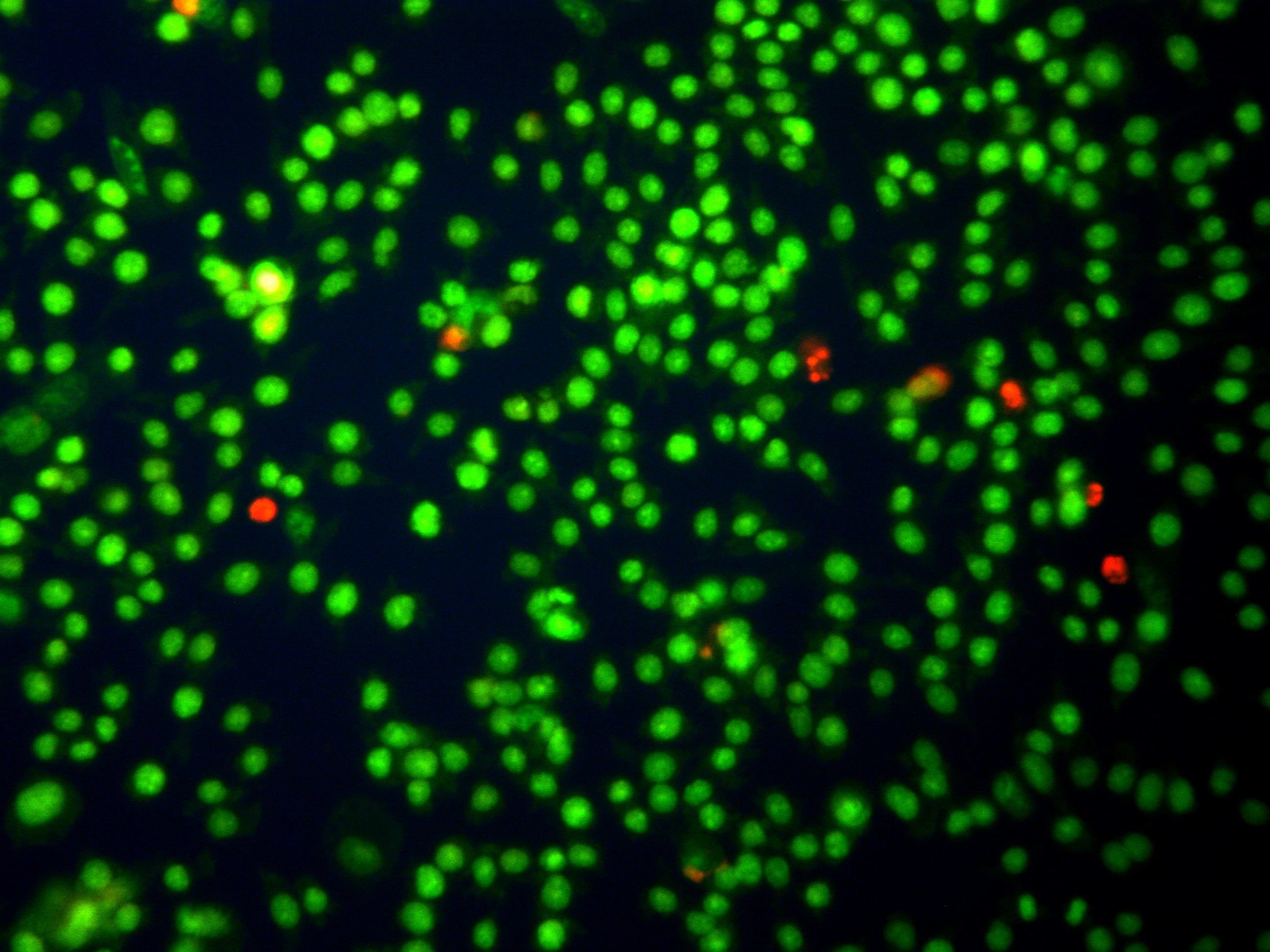

Supplement: S3 File — (ZIP) [file pone.0208866.s003.zip › S3_File/pone.0009826 EOC Replication Data 2018 (1 of 4)/cnt0005.tif]

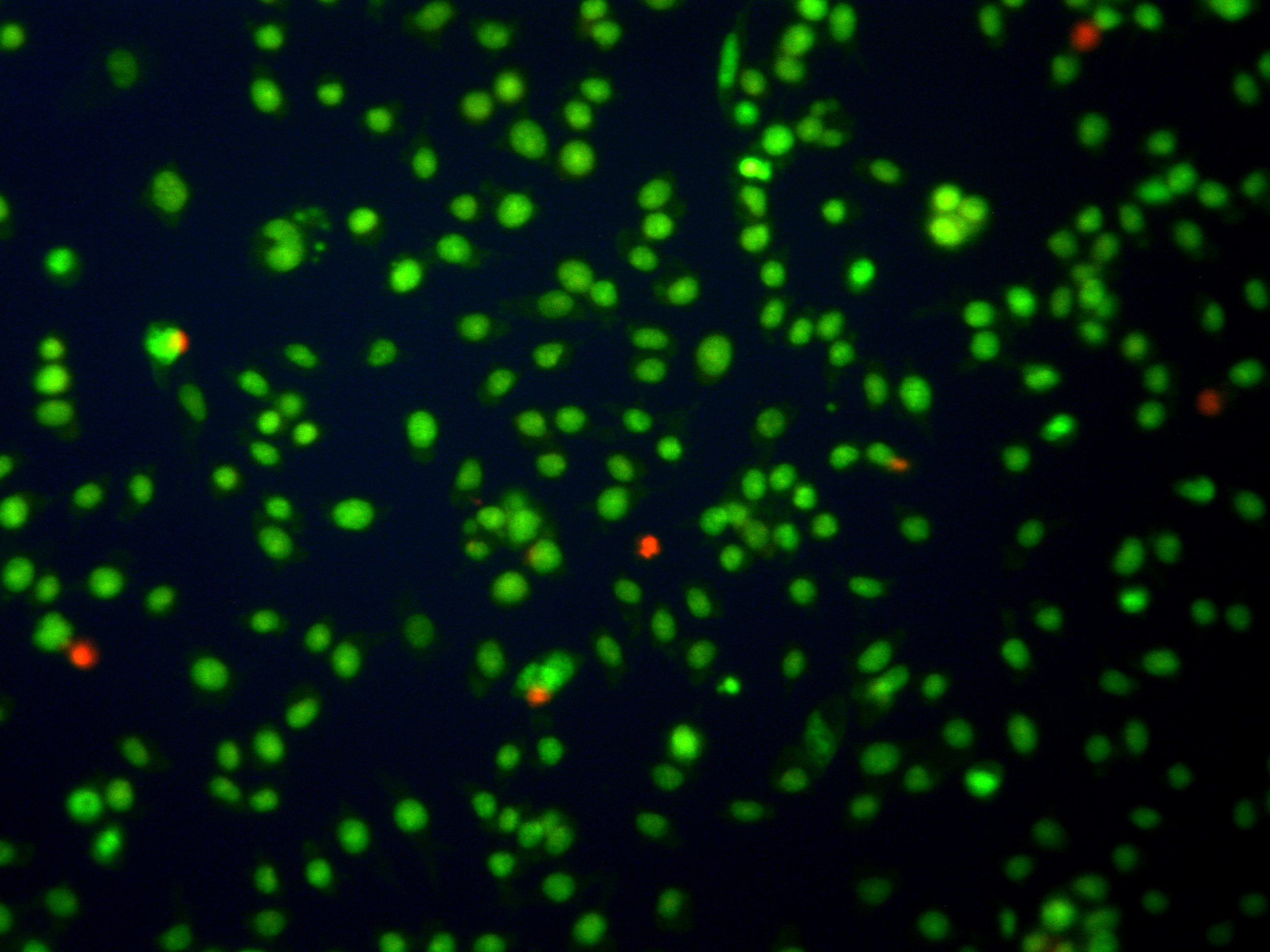

Supplement: S3 File — (ZIP) [file pone.0208866.s003.zip › S3_File/pone.0009826 EOC Replication Data 2018 (1 of 4)/cnt0006.tif]

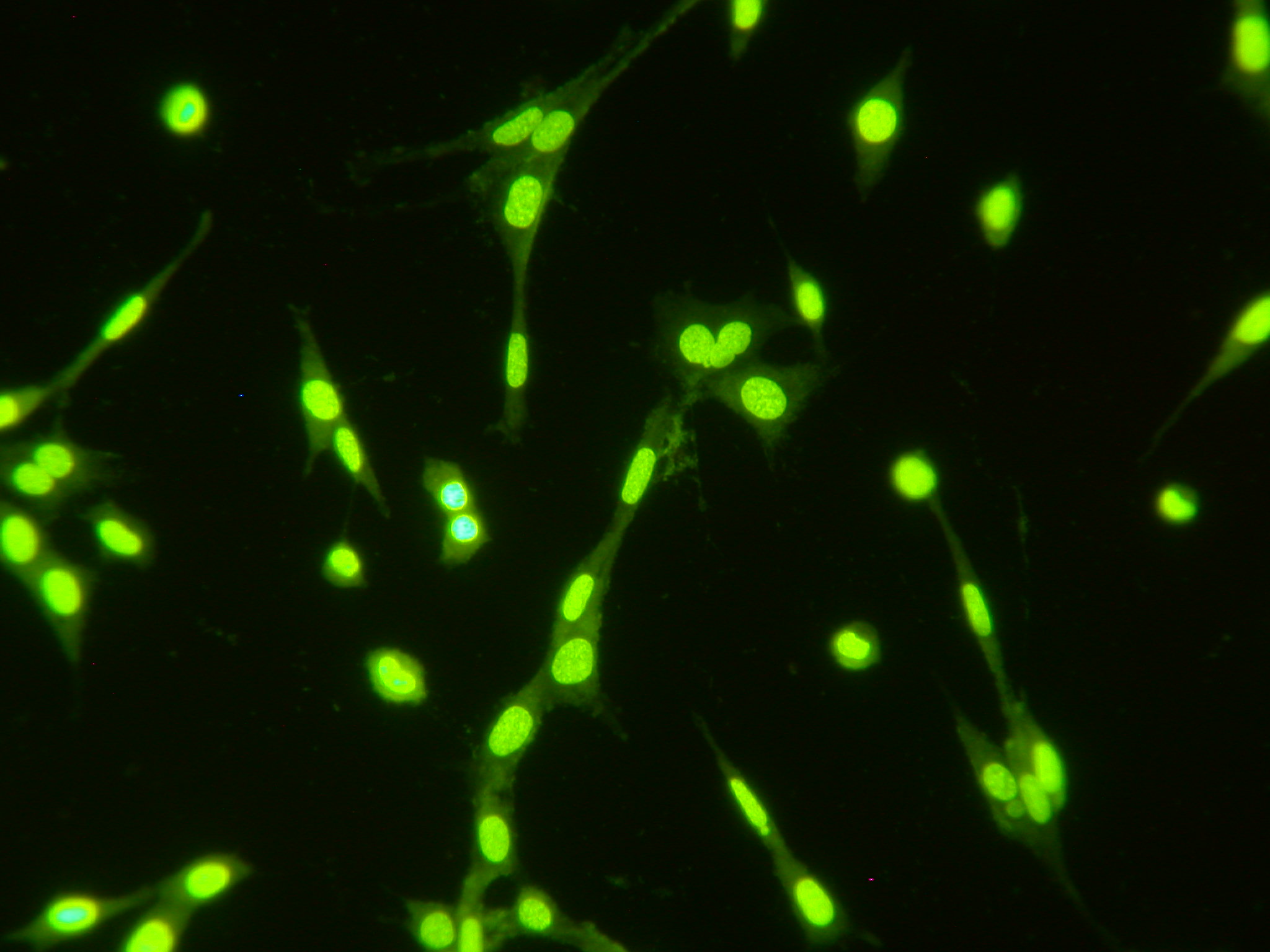

Supplement: S3 File — (ZIP) [file pone.0208866.s003.zip › S3_File/pone.0009826 EOC Replication Data 2018 (1 of 4)/DSCN9530.JPG]

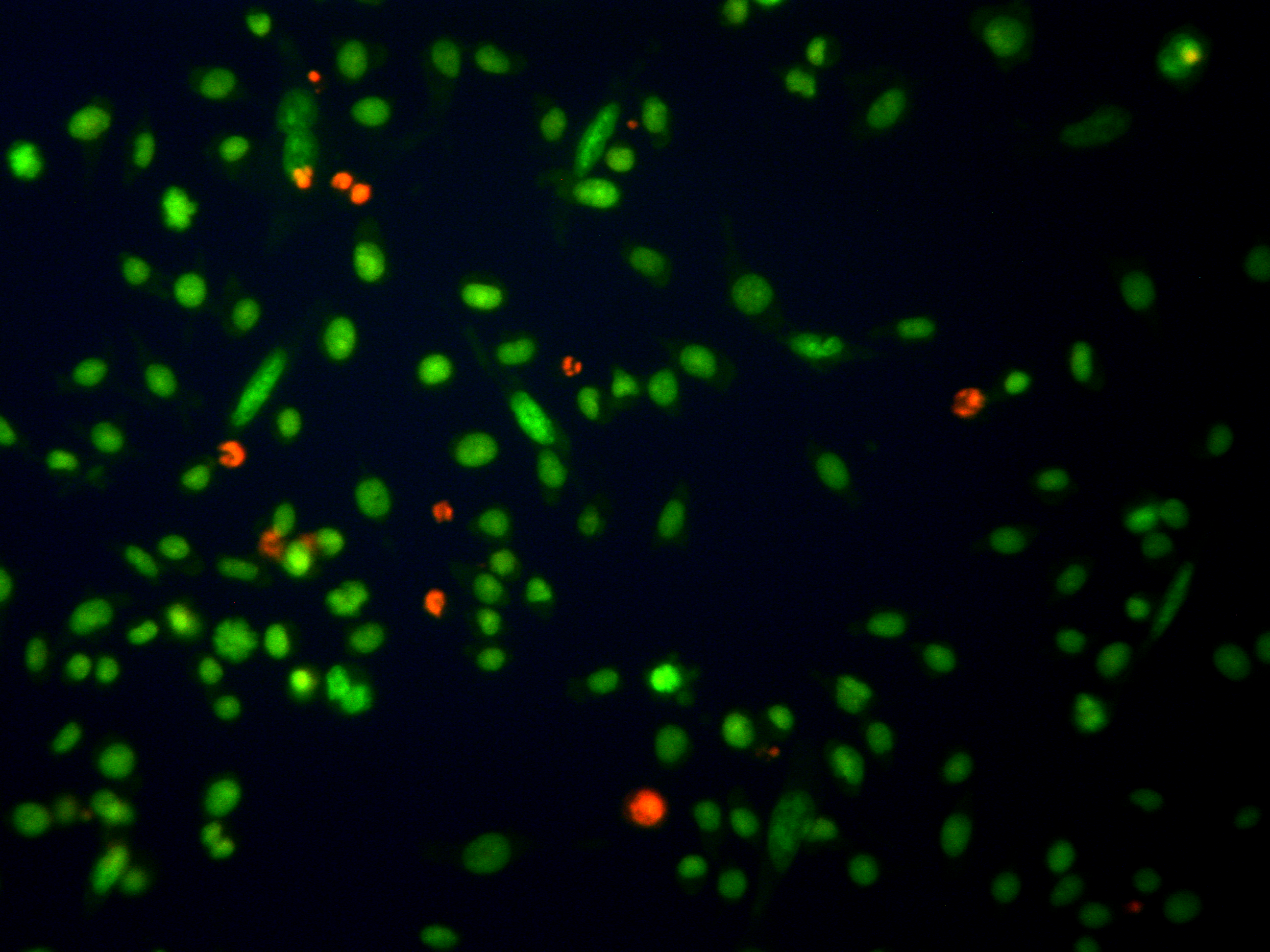

Supplement: S3 File — (ZIP) [file pone.0208866.s003.zip › S3_File/pone.0009826 EOC Replication Data 2018 (1 of 4)/tnf.tif]

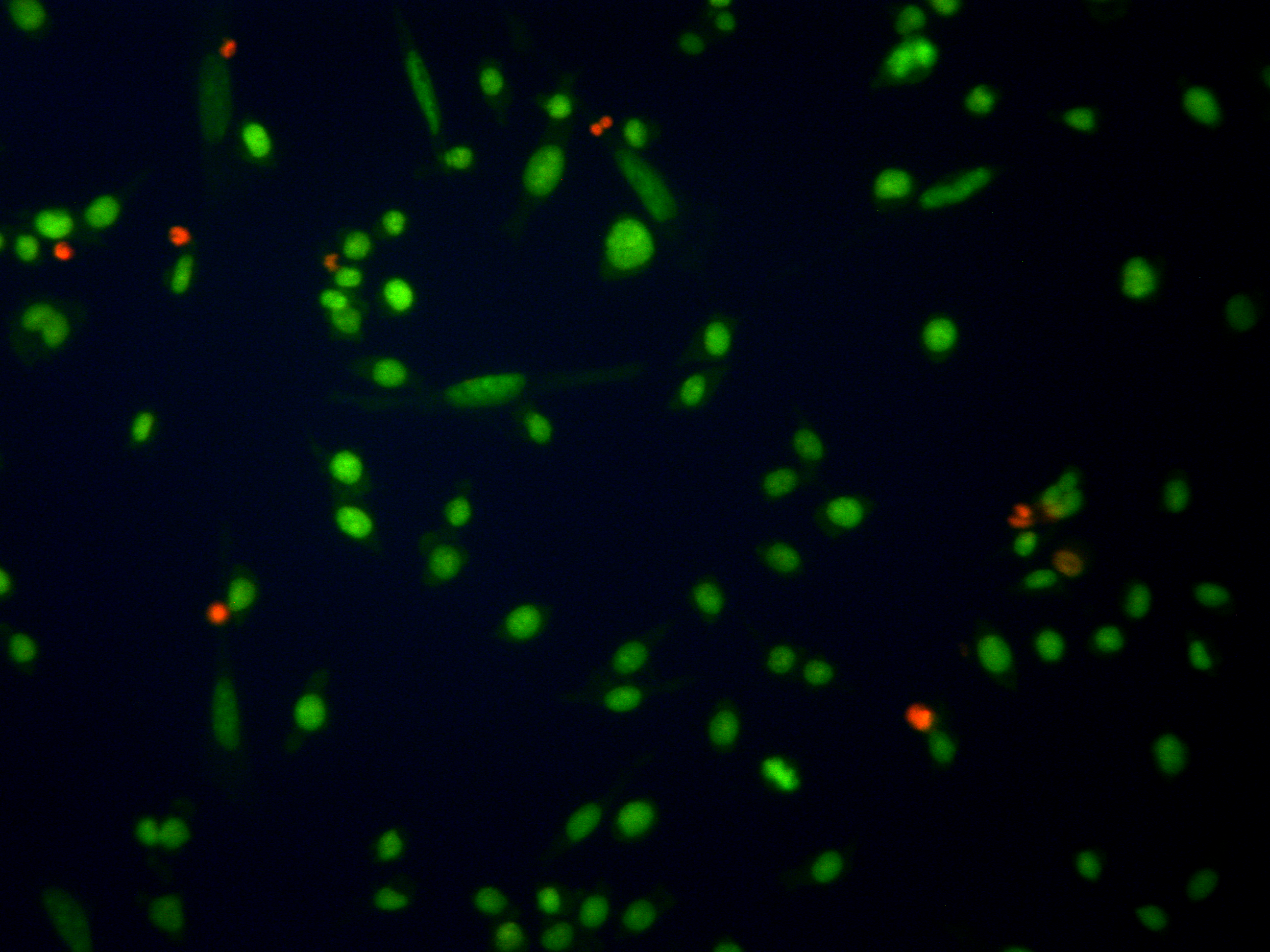

Supplement: S3 File — (ZIP) [file pone.0208866.s003.zip › S3_File/pone.0009826 EOC Replication Data 2018 (1 of 4)/tnf0001.tif]

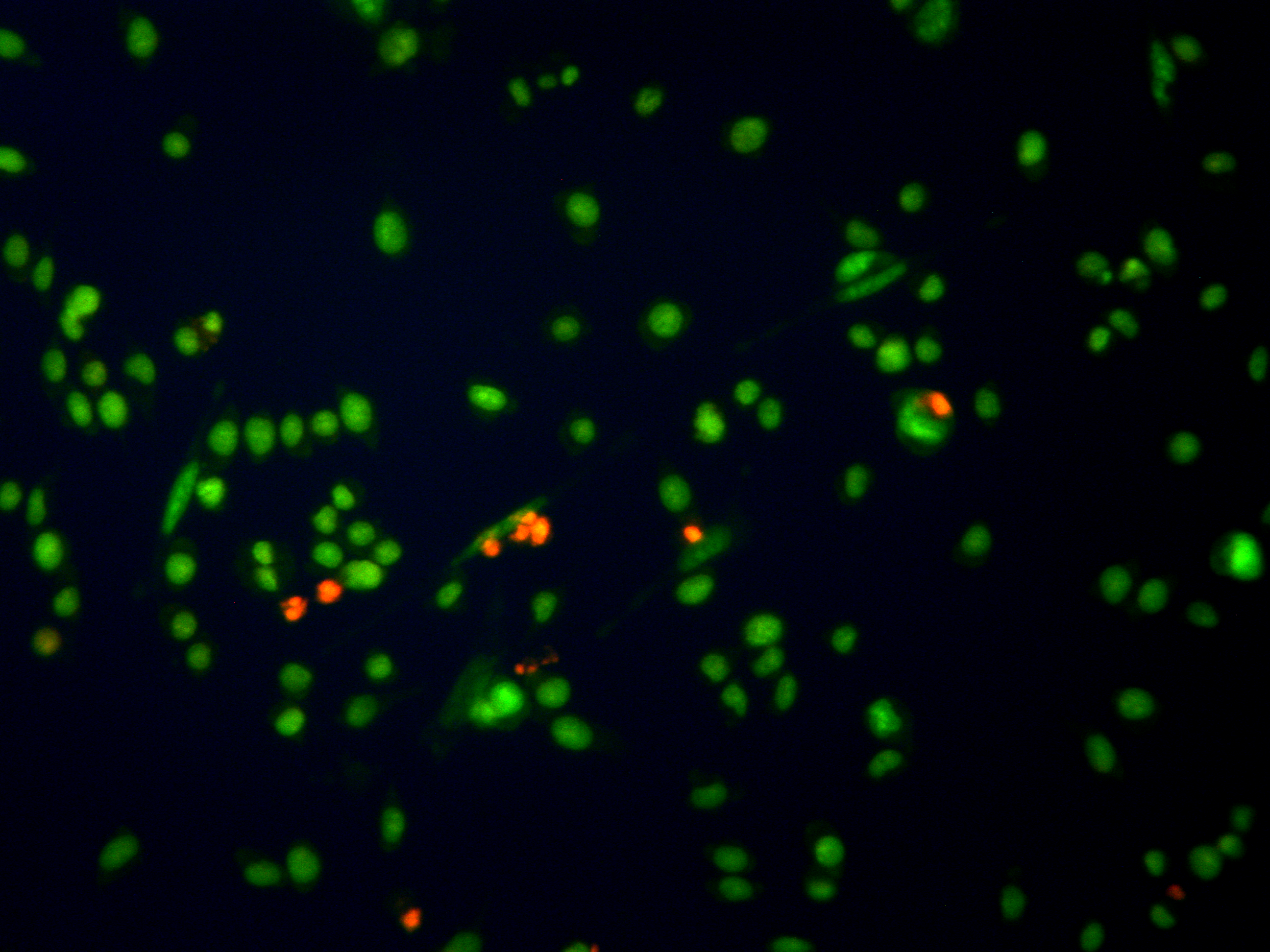

Supplement: S3 File — (ZIP) [file pone.0208866.s003.zip › S3_File/pone.0009826 EOC Replication Data 2018 (1 of 4)/tnf0002.tif]

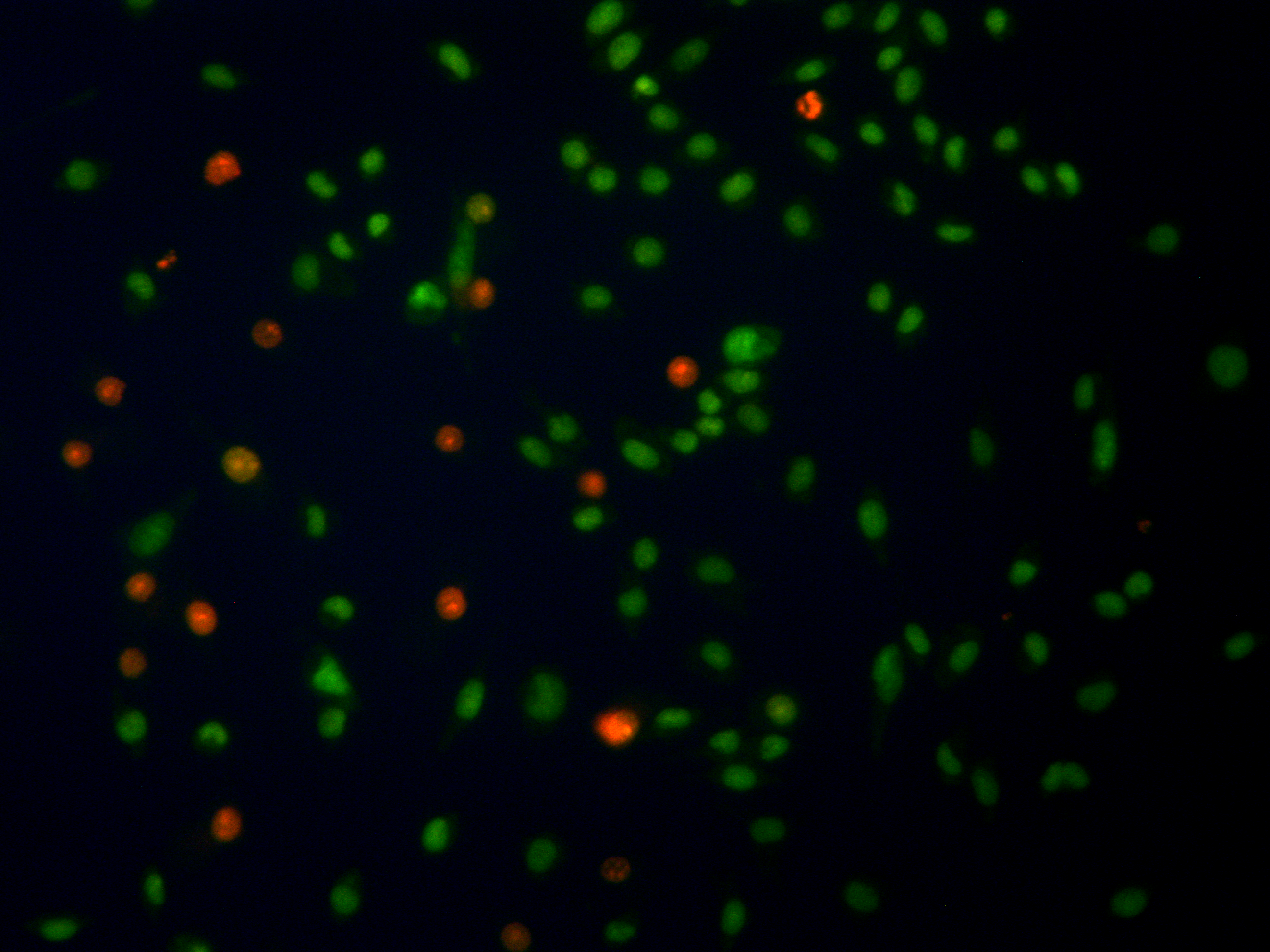

Supplement: S3 File — (ZIP) [file pone.0208866.s003.zip › S3_File/pone.0009826 EOC Replication Data 2018 (1 of 4)/tnf0003.tif]

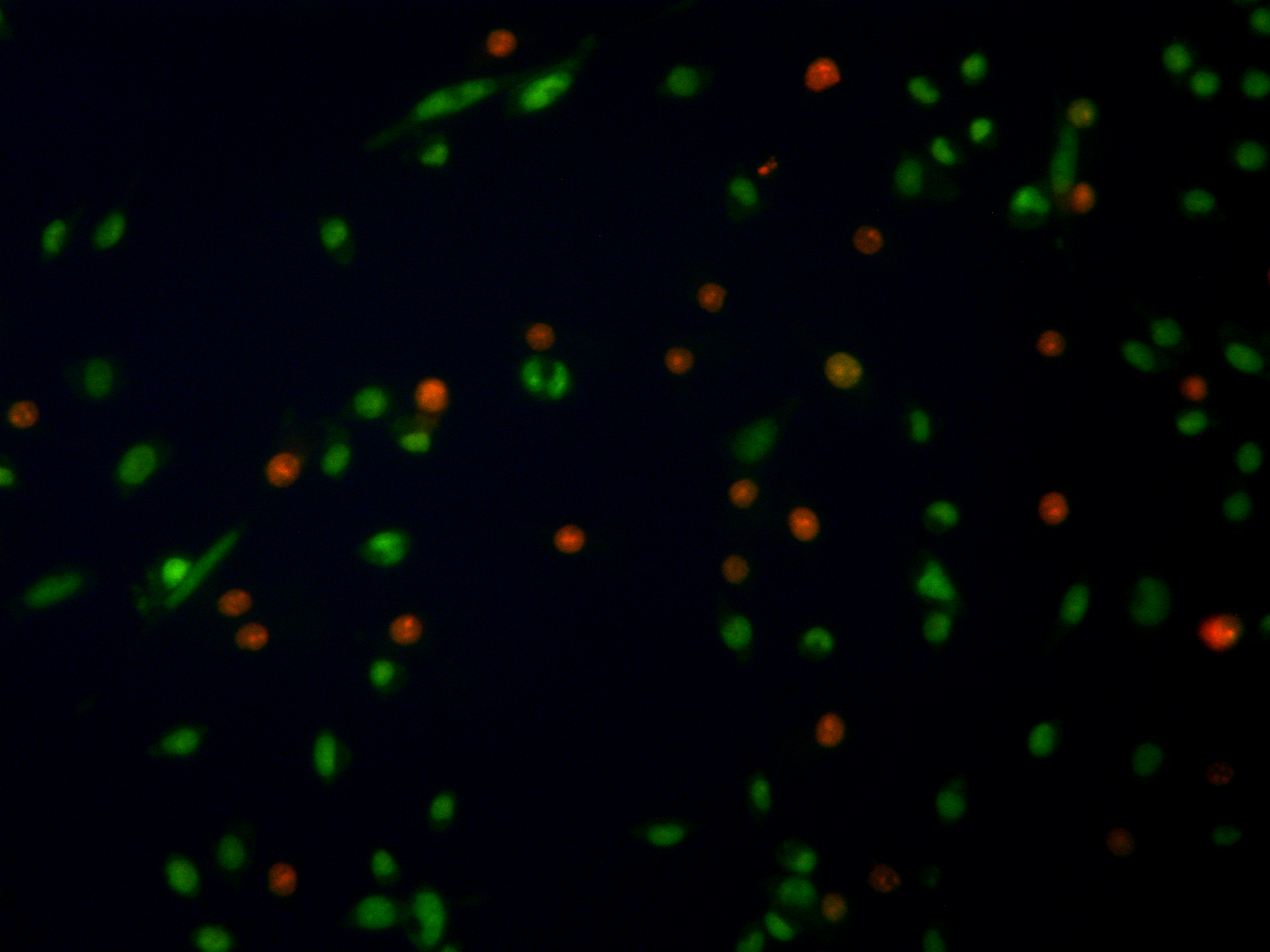

Supplement: S3 File — (ZIP) [file pone.0208866.s003.zip › S3_File/pone.0009826 EOC Replication Data 2018 (1 of 4)/tnf0004.tif]

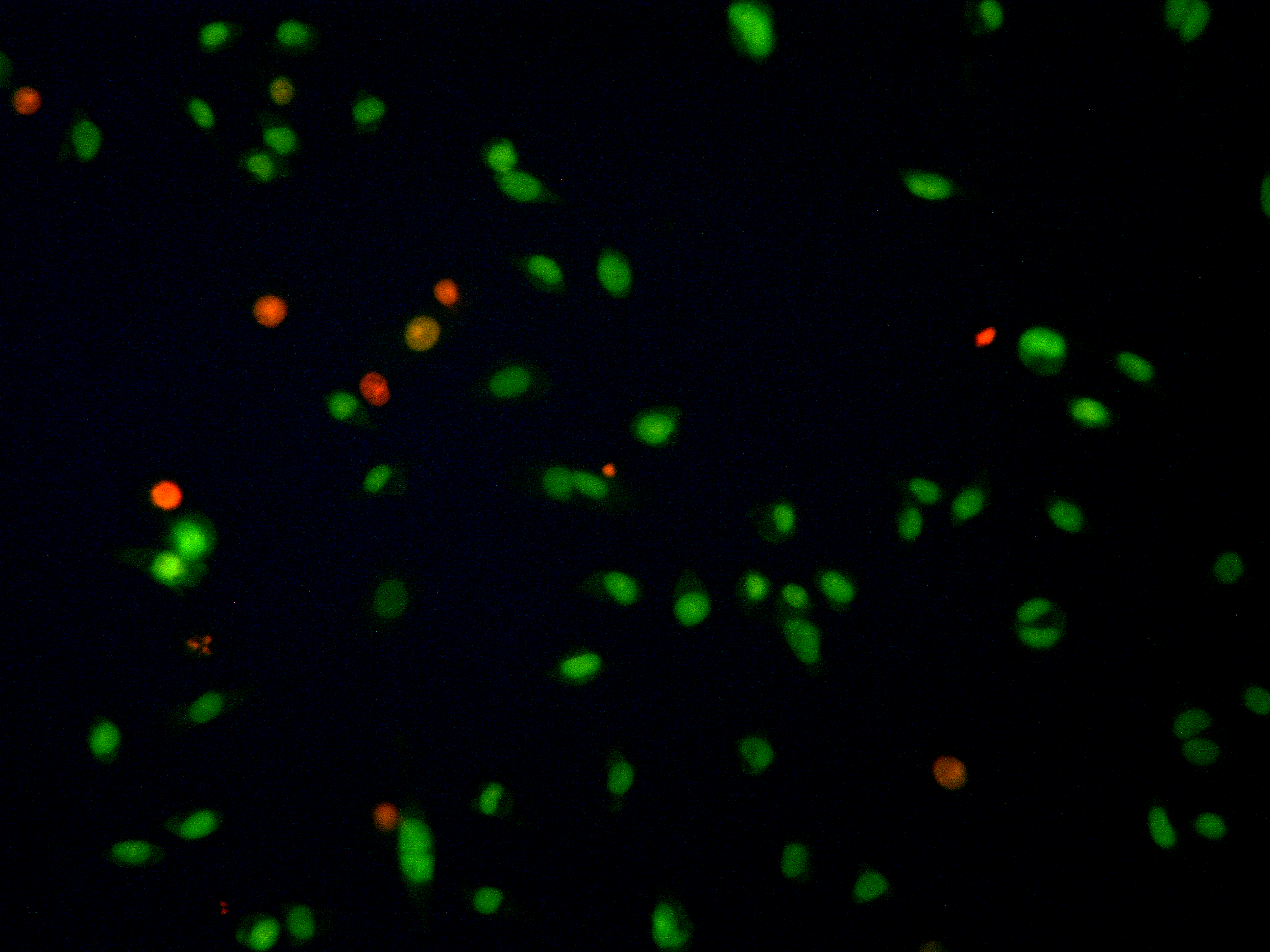

Supplement: S3 File — (ZIP) [file pone.0208866.s003.zip › S3_File/pone.0009826 EOC Replication Data 2018 (1 of 4)/tnf0006.tif]

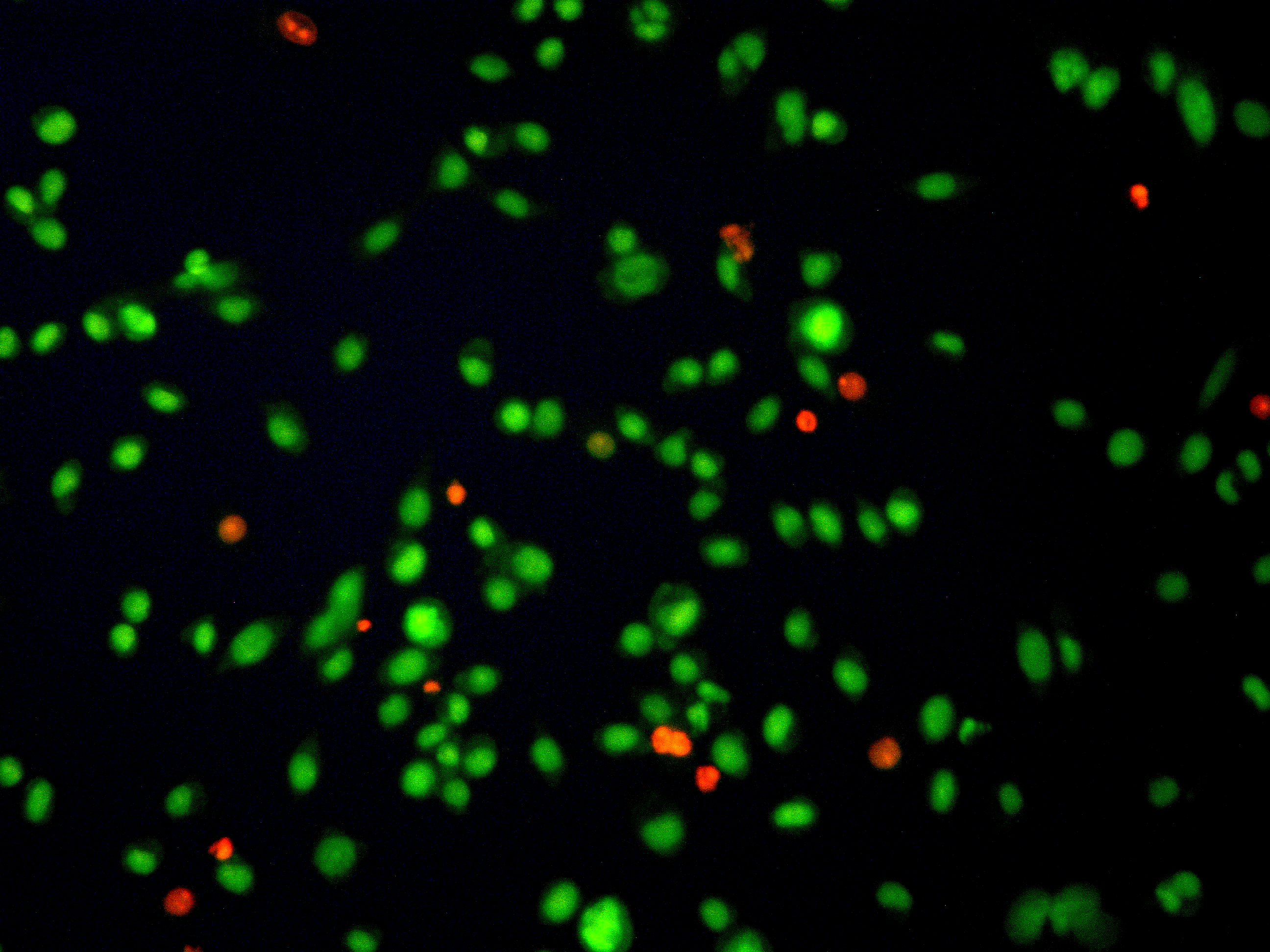

Supplement: S3 File — (ZIP) [file pone.0208866.s003.zip › S3_File/pone.0009826 EOC Replication Data 2018 (1 of 4)/tnf0007.tif]

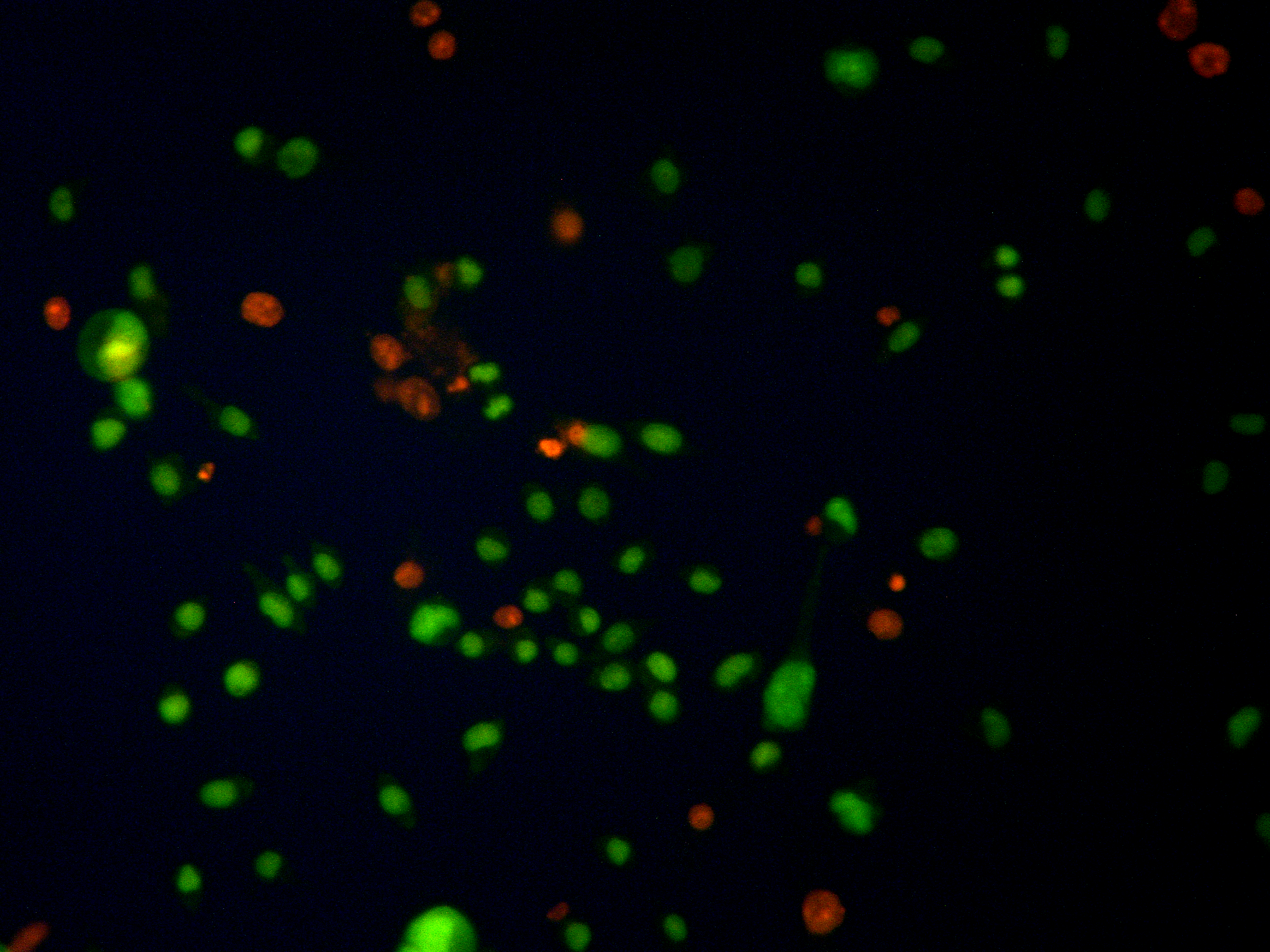

Supplement: S3 File — (ZIP) [file pone.0208866.s003.zip › S3_File/pone.0009826 EOC Replication Data 2018 (1 of 4)/tnf0008.tif]

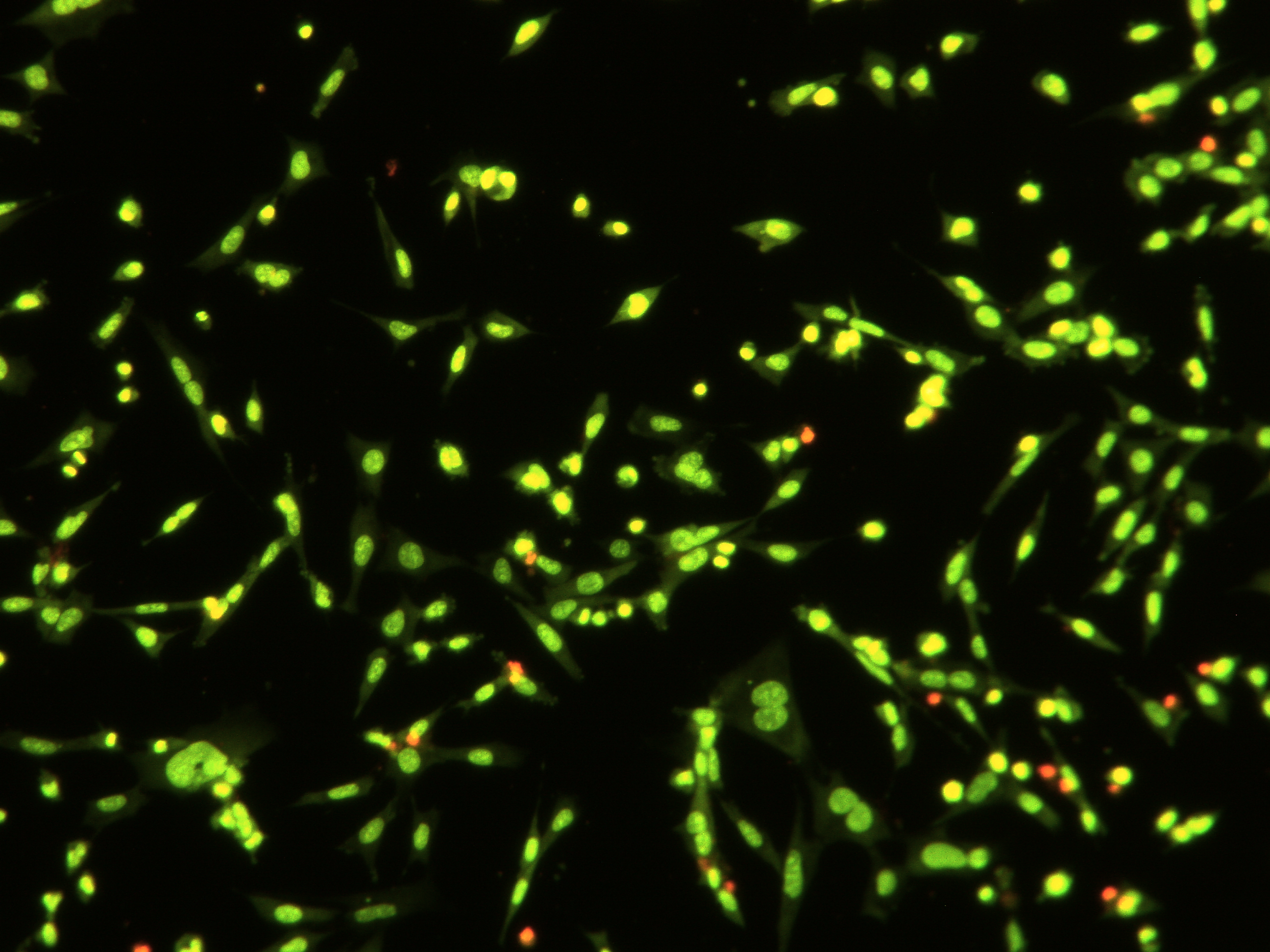

Supplement: S3 File — (ZIP) [file pone.0208866.s003.zip › S3_File/pone.0009826 EOC Replication Data 2018 (1 of 4)/wt cnt 0001.tif]

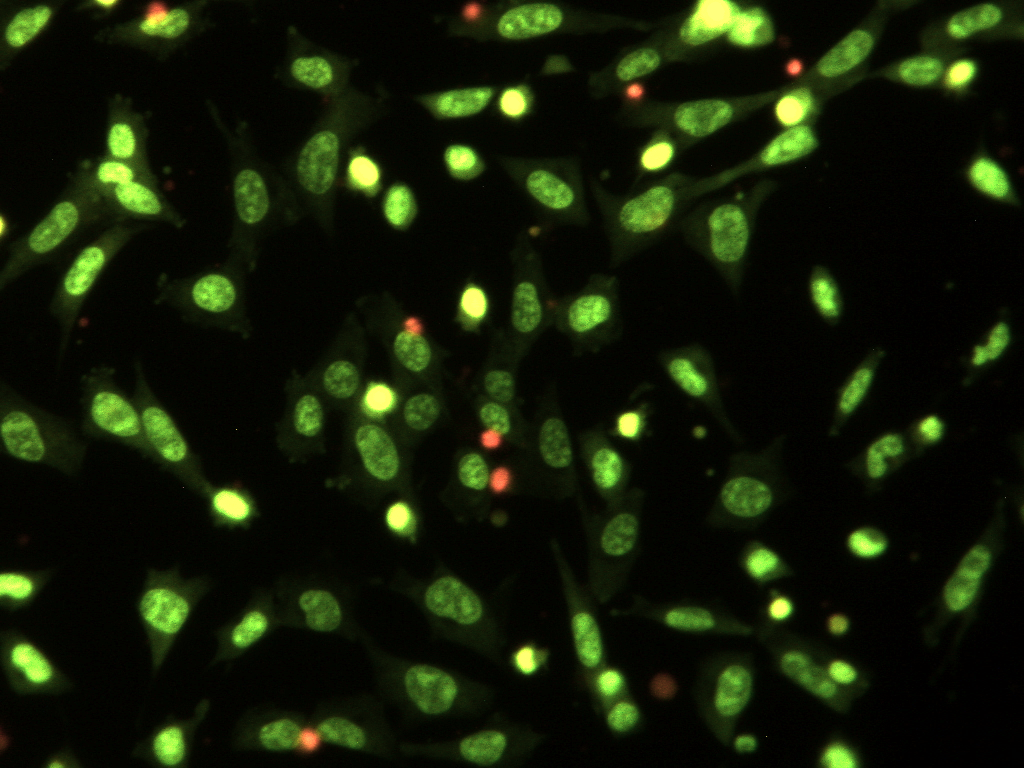

Supplement: S3 File — (ZIP) [file pone.0208866.s003.zip › S3_File/pone.0009826 EOC Replication Data 2018 (1 of 4)/wt cnt 0001_001.tif]

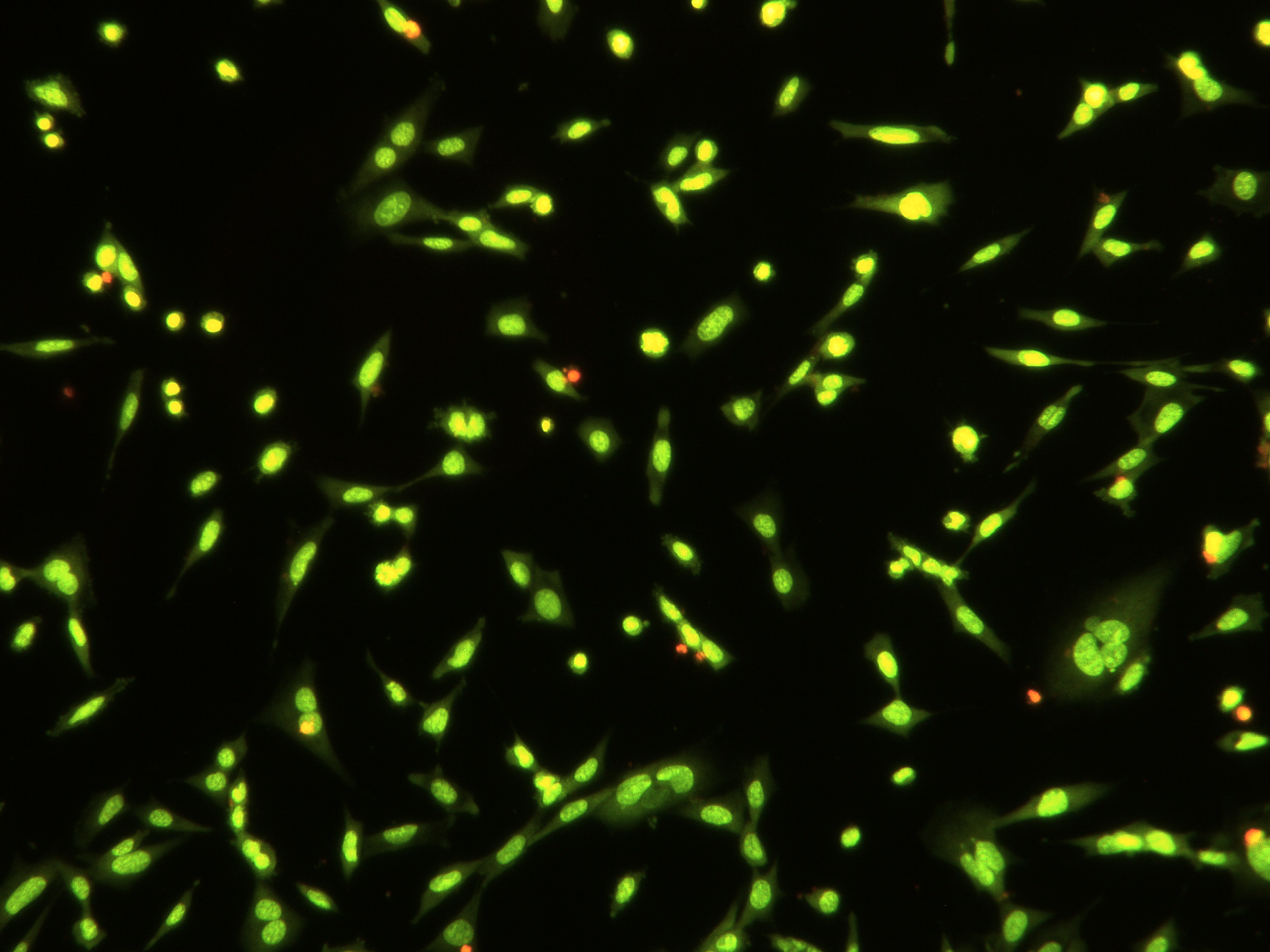

Supplement: S4 File — (ZIP) [file pone.0208866.s004.zip › S4_File/pone.0009826 EOC Replication Data 2018 (2 of 4)/wt cnt 0002.tif]

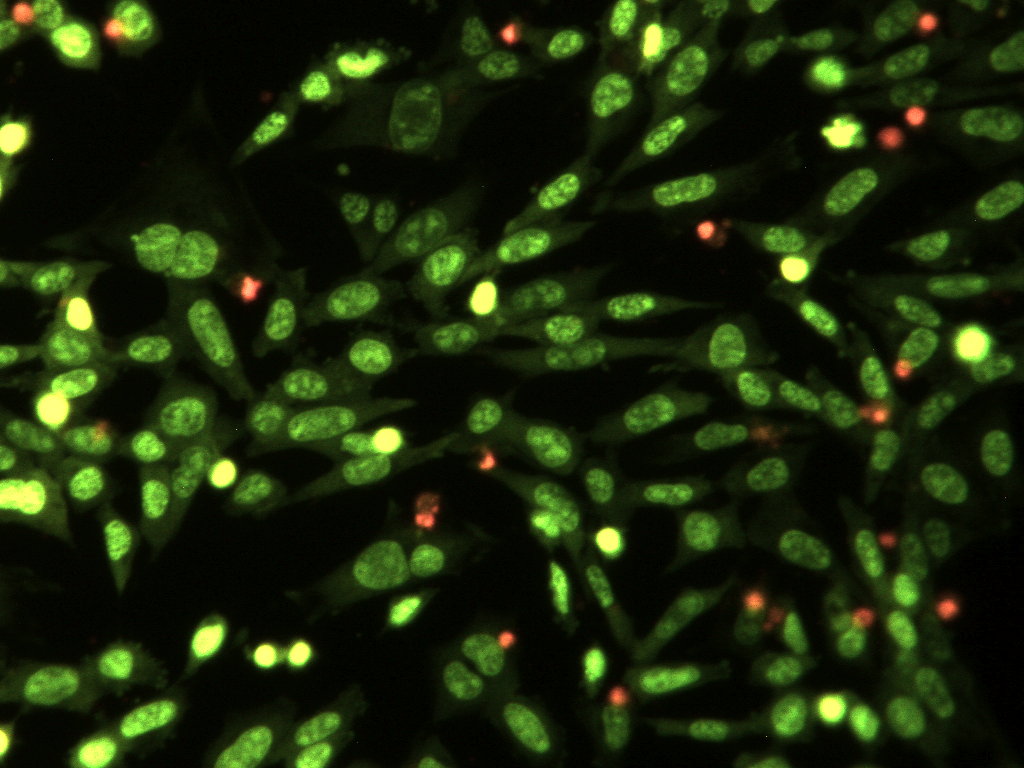

Supplement: S4 File — (ZIP) [file pone.0208866.s004.zip › S4_File/pone.0009826 EOC Replication Data 2018 (2 of 4)/wt cnt 0002_001.tif]

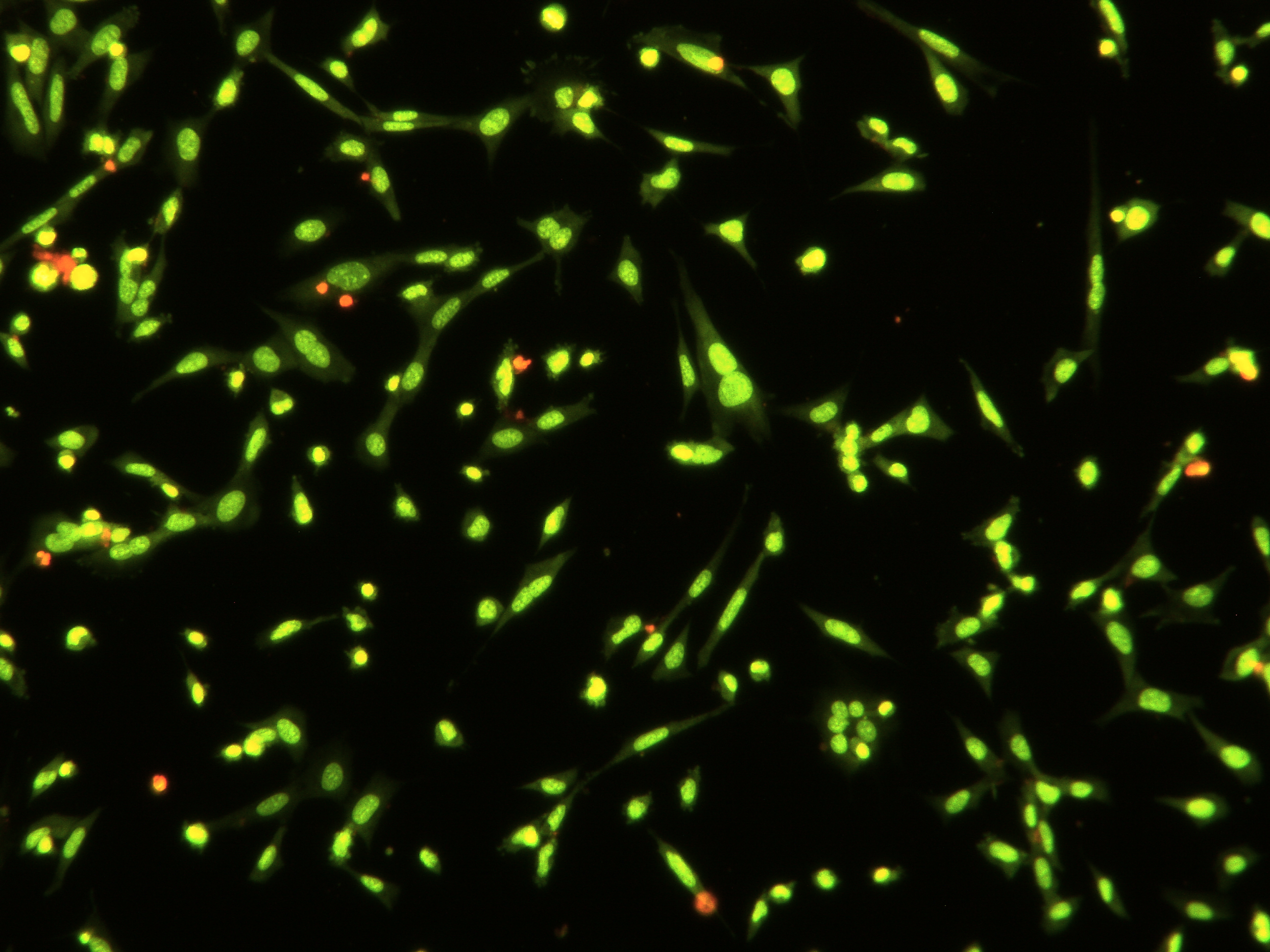

Supplement: S4 File — (ZIP) [file pone.0208866.s004.zip › S4_File/pone.0009826 EOC Replication Data 2018 (2 of 4)/wt cnt 0003.tif]

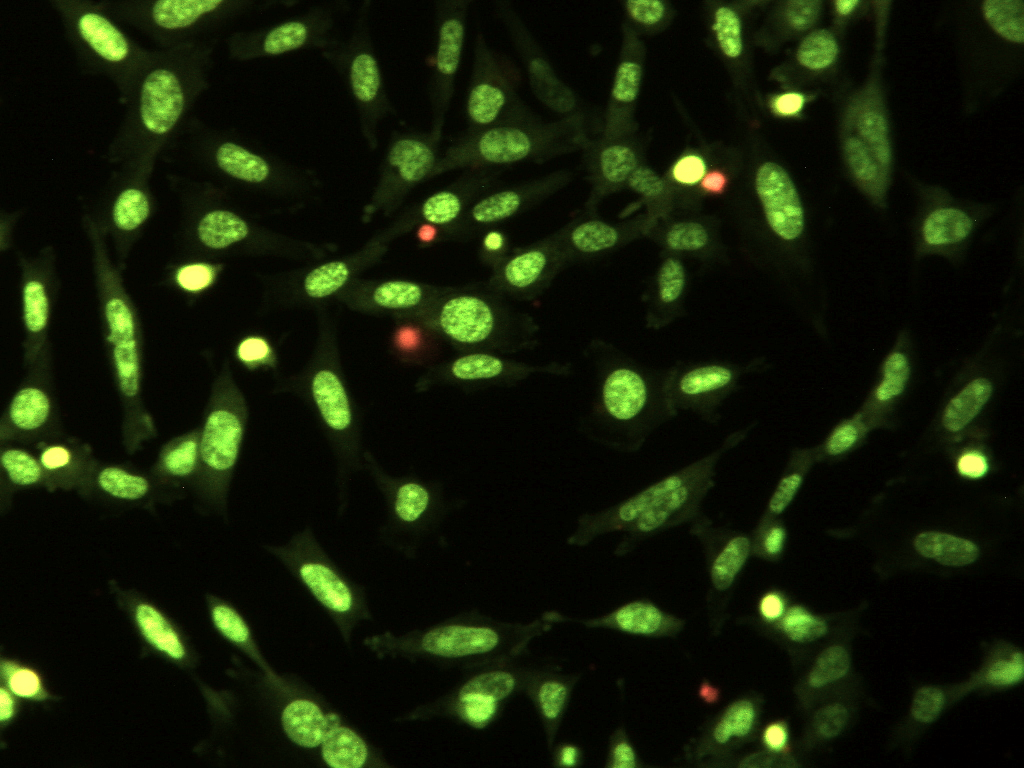

Supplement: S4 File — (ZIP) [file pone.0208866.s004.zip › S4_File/pone.0009826 EOC Replication Data 2018 (2 of 4)/wt cnt 0003_001.tif]

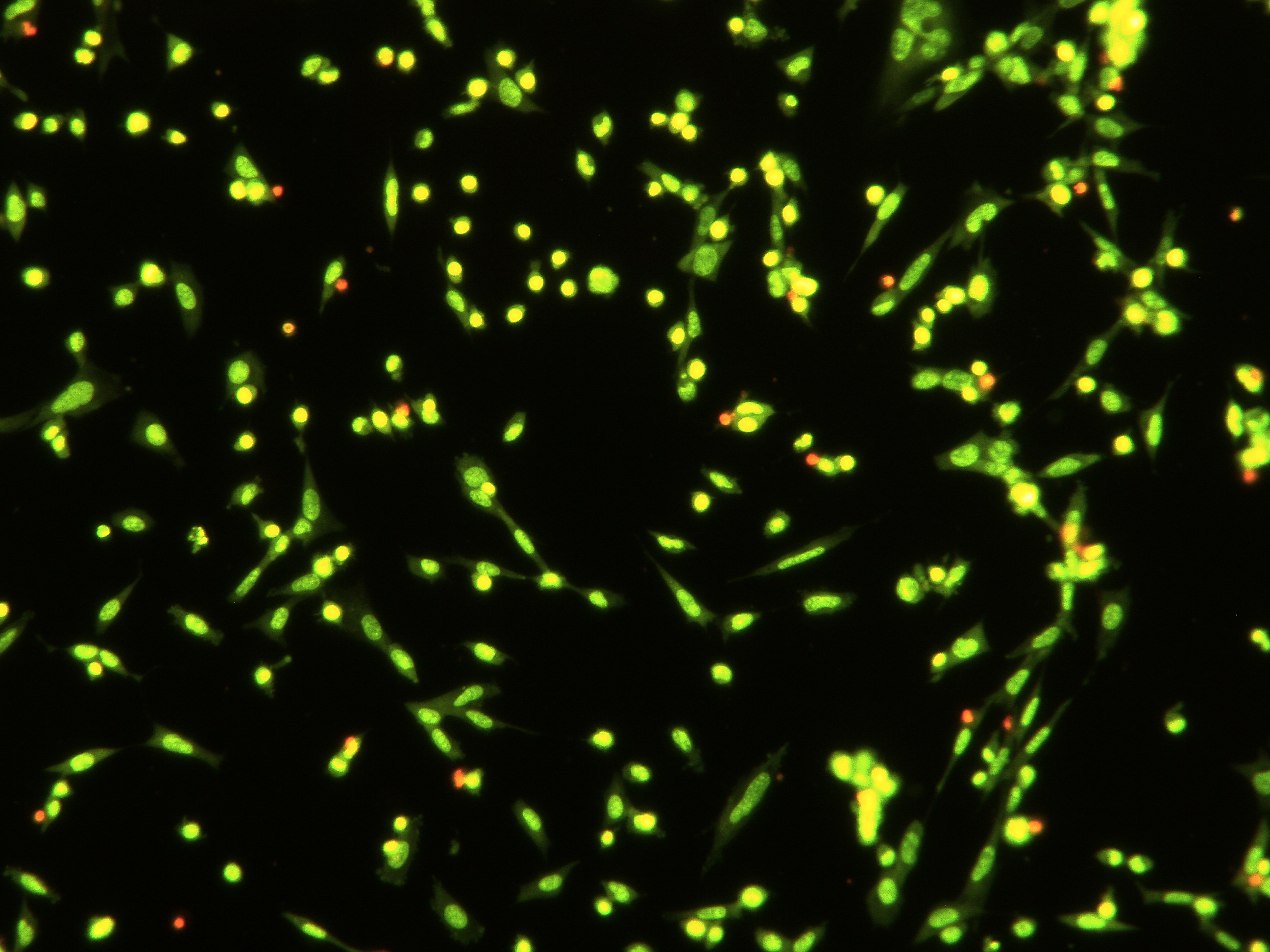

Supplement: S4 File — (ZIP) [file pone.0208866.s004.zip › S4_File/pone.0009826 EOC Replication Data 2018 (2 of 4)/wt cnt 0004.tif]

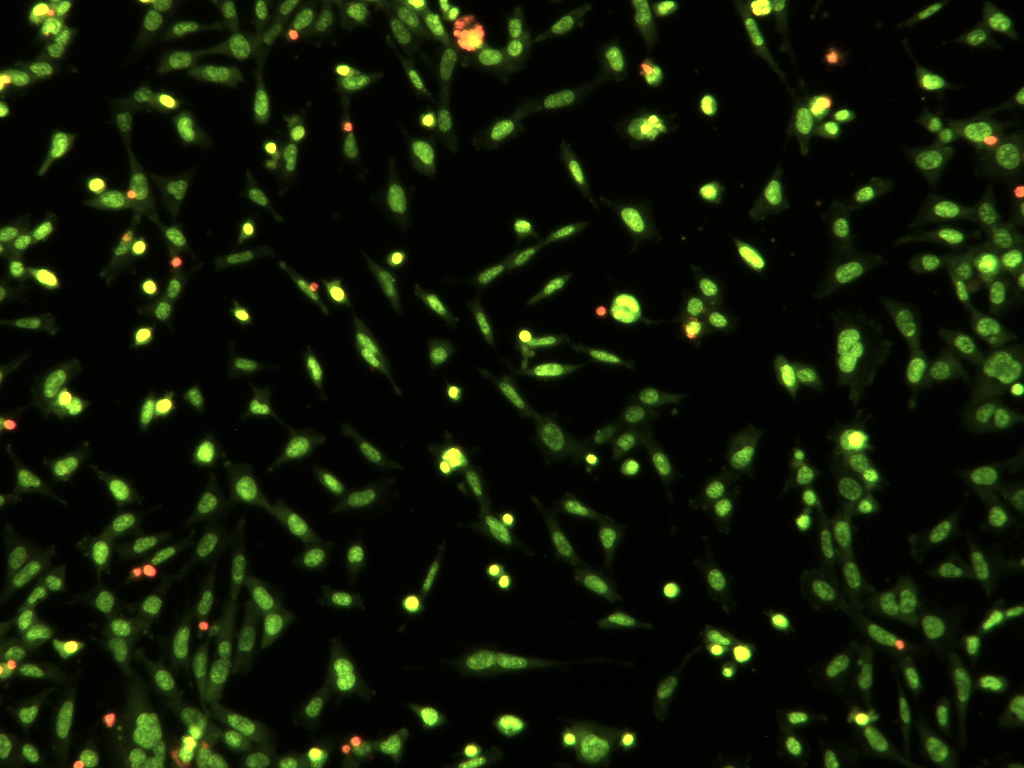

Supplement: S4 File — (ZIP) [file pone.0208866.s004.zip › S4_File/pone.0009826 EOC Replication Data 2018 (2 of 4)/wt cnt 0004_001.tif]

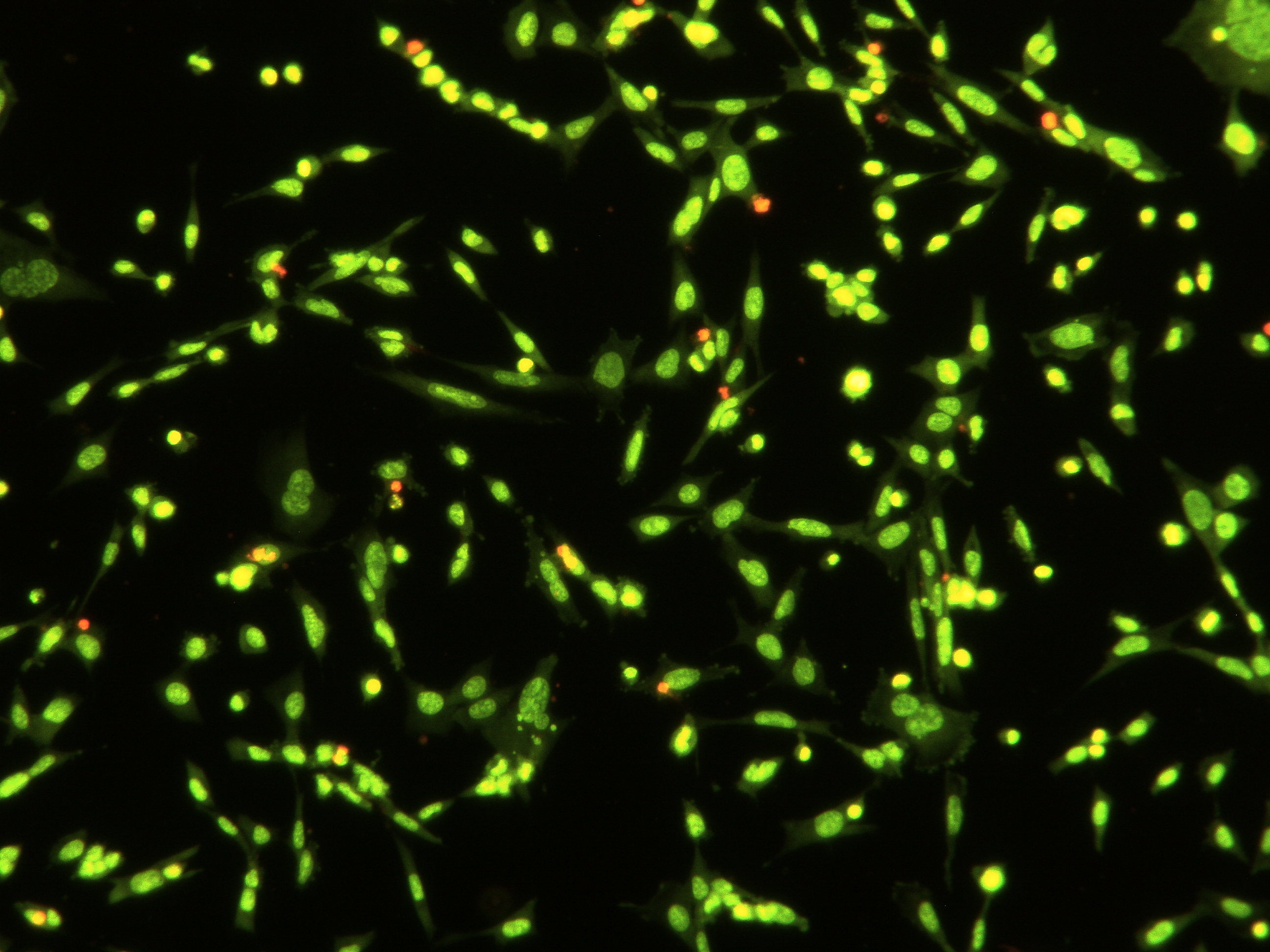

Supplement: S4 File — (ZIP) [file pone.0208866.s004.zip › S4_File/pone.0009826 EOC Replication Data 2018 (2 of 4)/wt cnt 0005.tif]

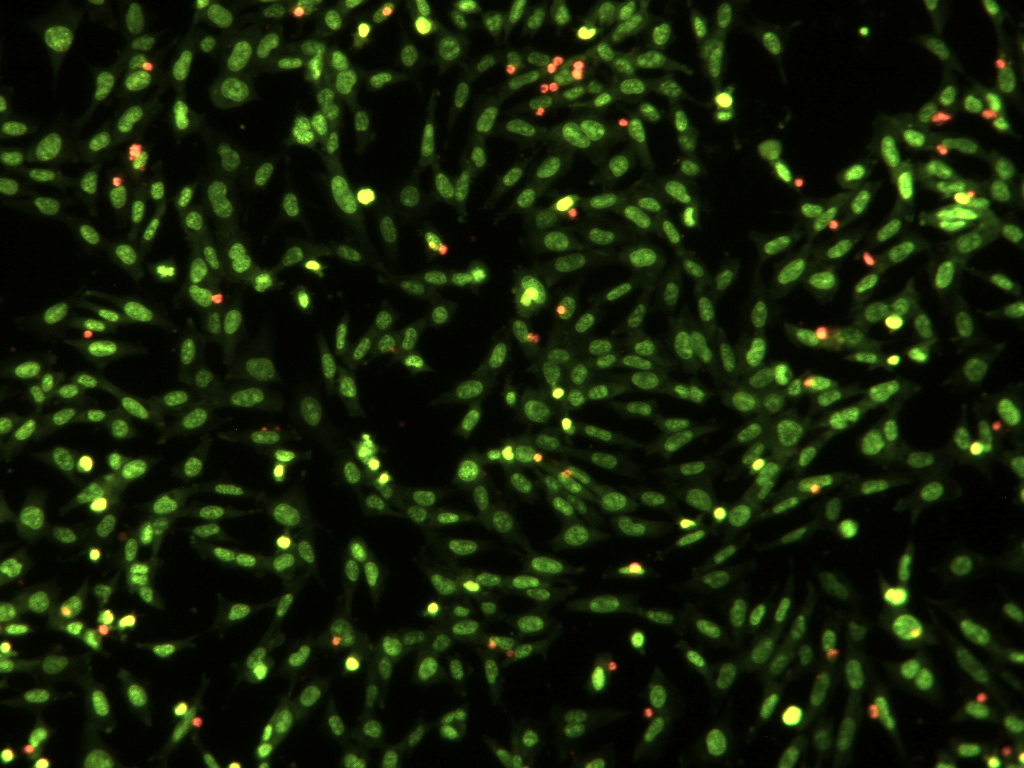

Supplement: S4 File — (ZIP) [file pone.0208866.s004.zip › S4_File/pone.0009826 EOC Replication Data 2018 (2 of 4)/wt cnt 0005_001.tif]

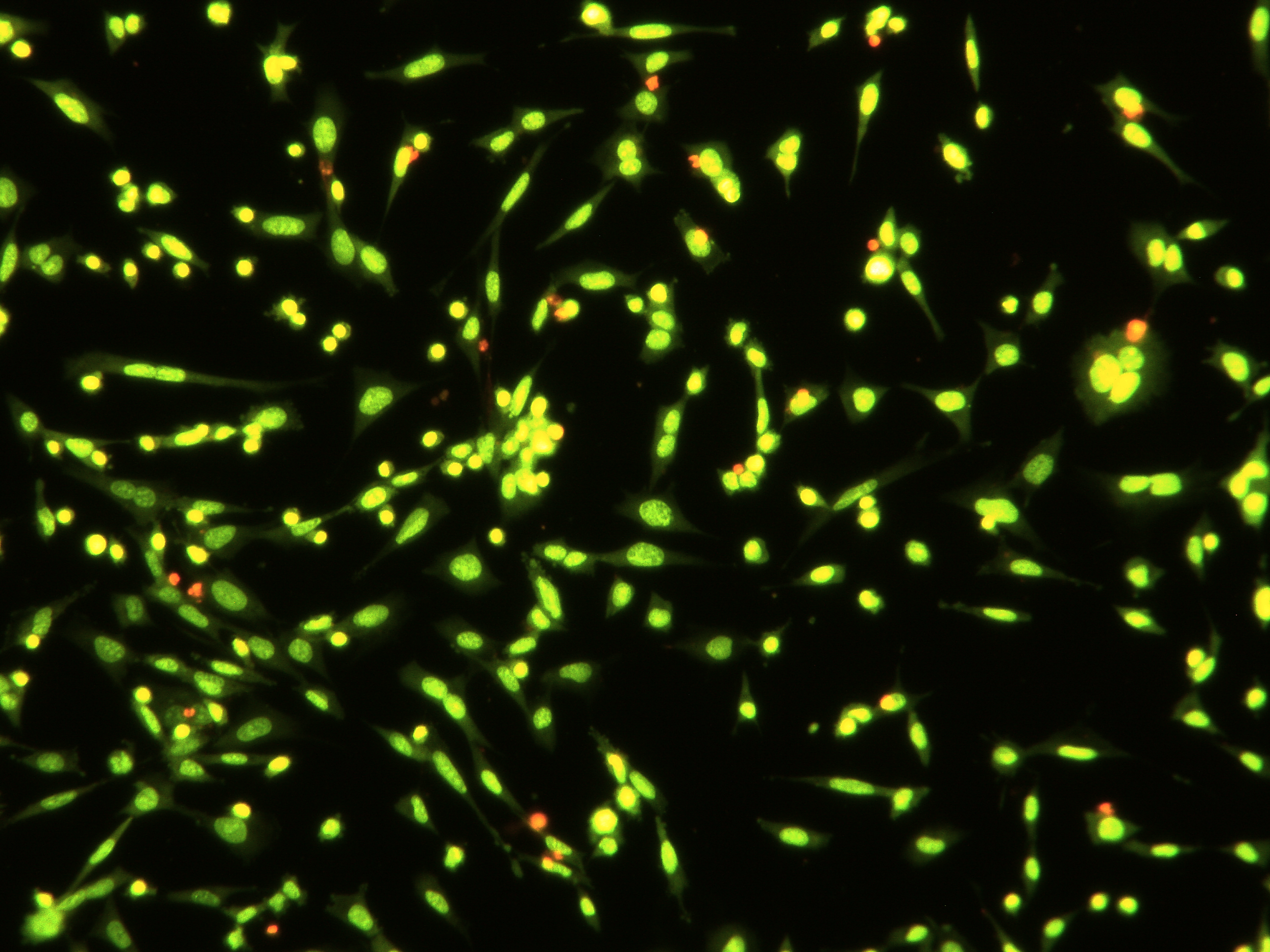

Supplement: S4 File — (ZIP) [file pone.0208866.s004.zip › S4_File/pone.0009826 EOC Replication Data 2018 (2 of 4)/wt cnt 0006.tif]

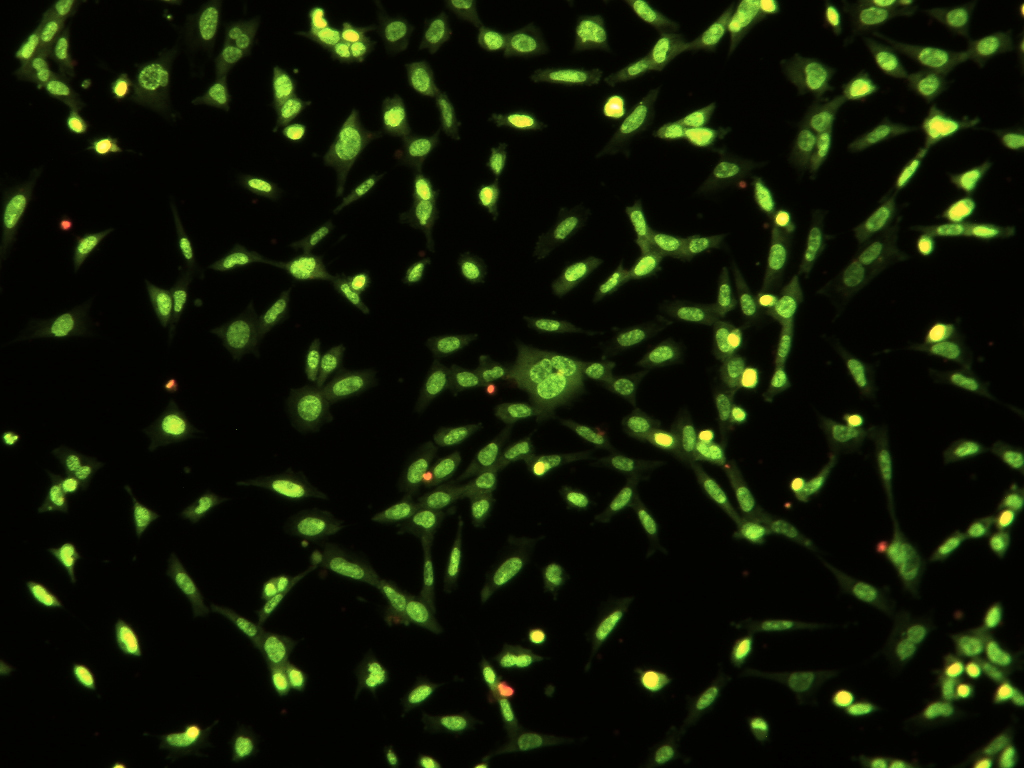

Supplement: S4 File — (ZIP) [file pone.0208866.s004.zip › S4_File/pone.0009826 EOC Replication Data 2018 (2 of 4)/wt cnt 0006_001.tif]

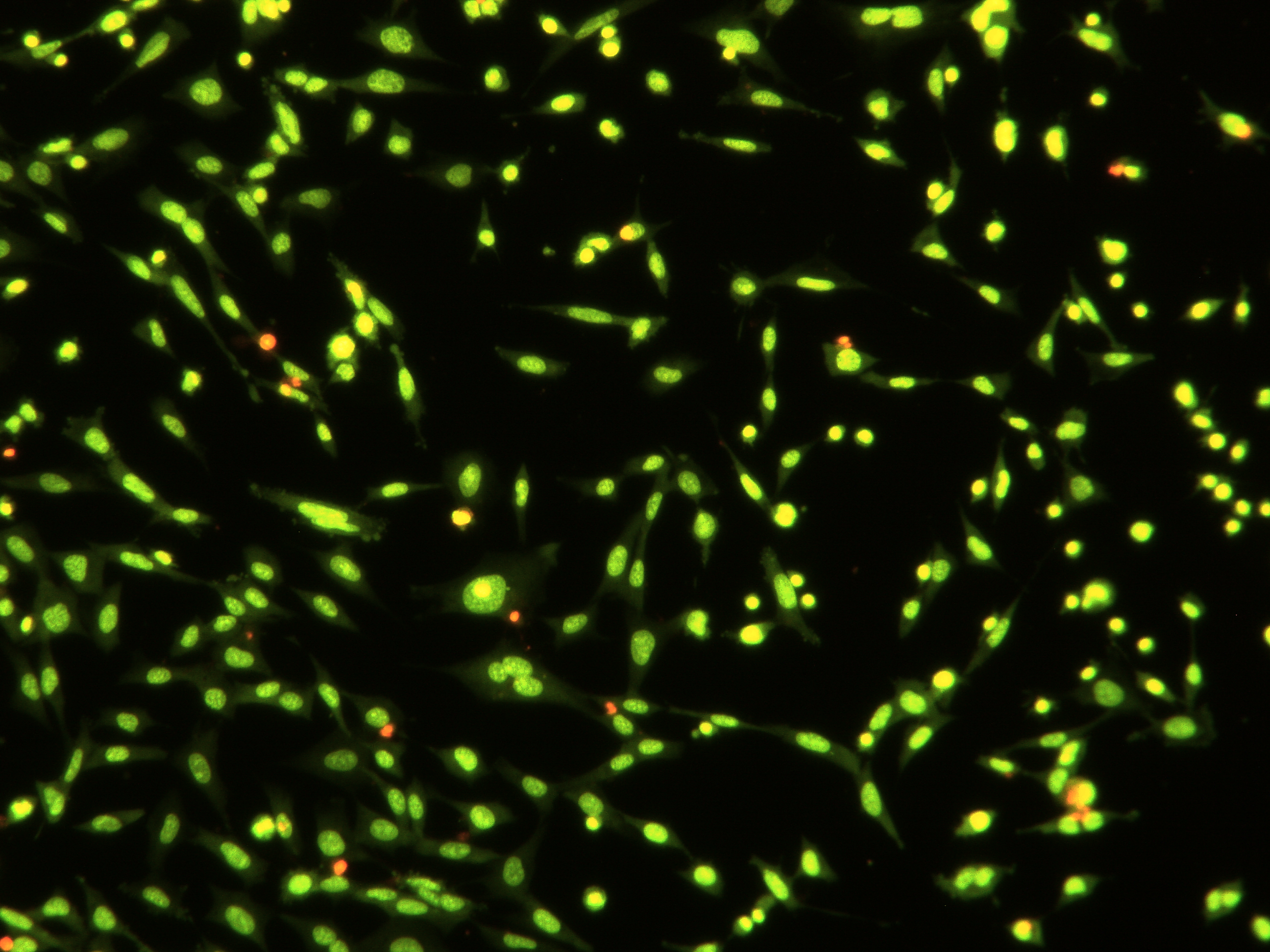

Supplement: S4 File — (ZIP) [file pone.0208866.s004.zip › S4_File/pone.0009826 EOC Replication Data 2018 (2 of 4)/wt cnt 0007.tif]

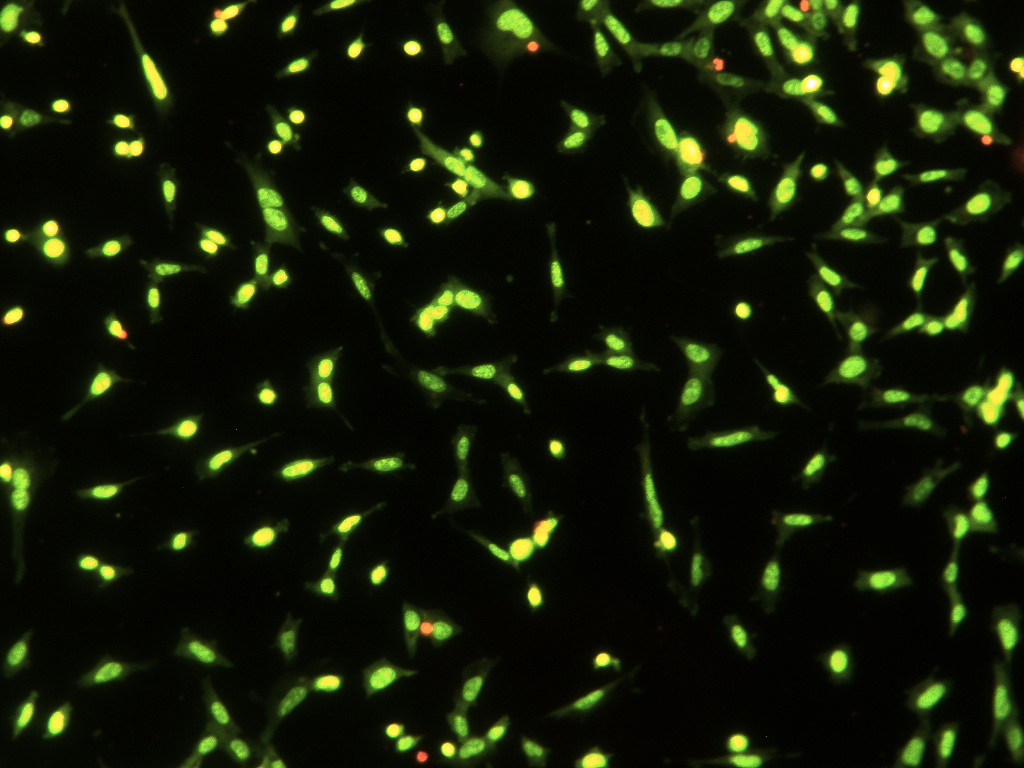

Supplement: S4 File — (ZIP) [file pone.0208866.s004.zip › S4_File/pone.0009826 EOC Replication Data 2018 (2 of 4)/wt cnt 0007_001.tif]

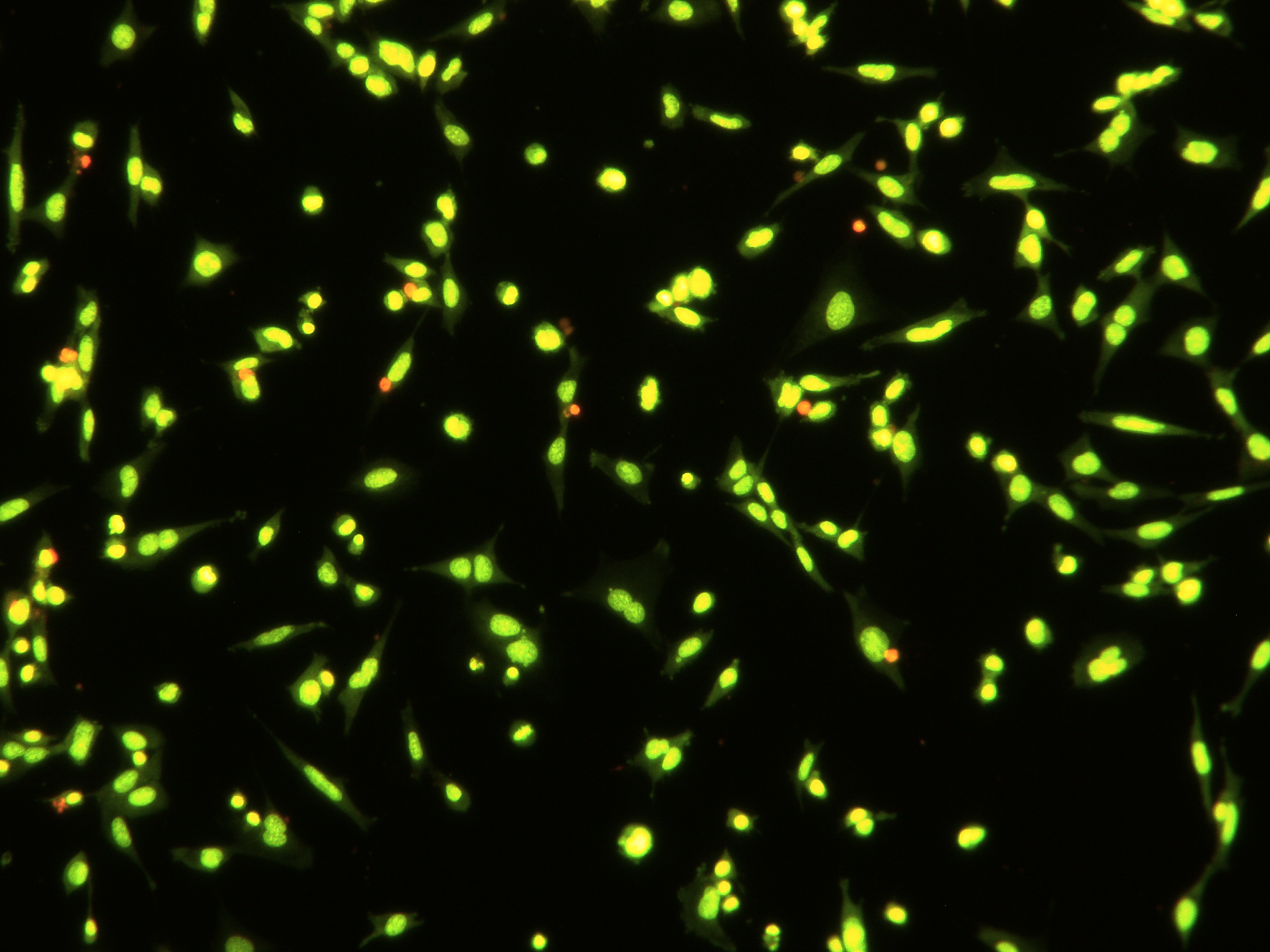

Supplement: S4 File — (ZIP) [file pone.0208866.s004.zip › S4_File/pone.0009826 EOC Replication Data 2018 (2 of 4)/wt cnt 0008.tif]

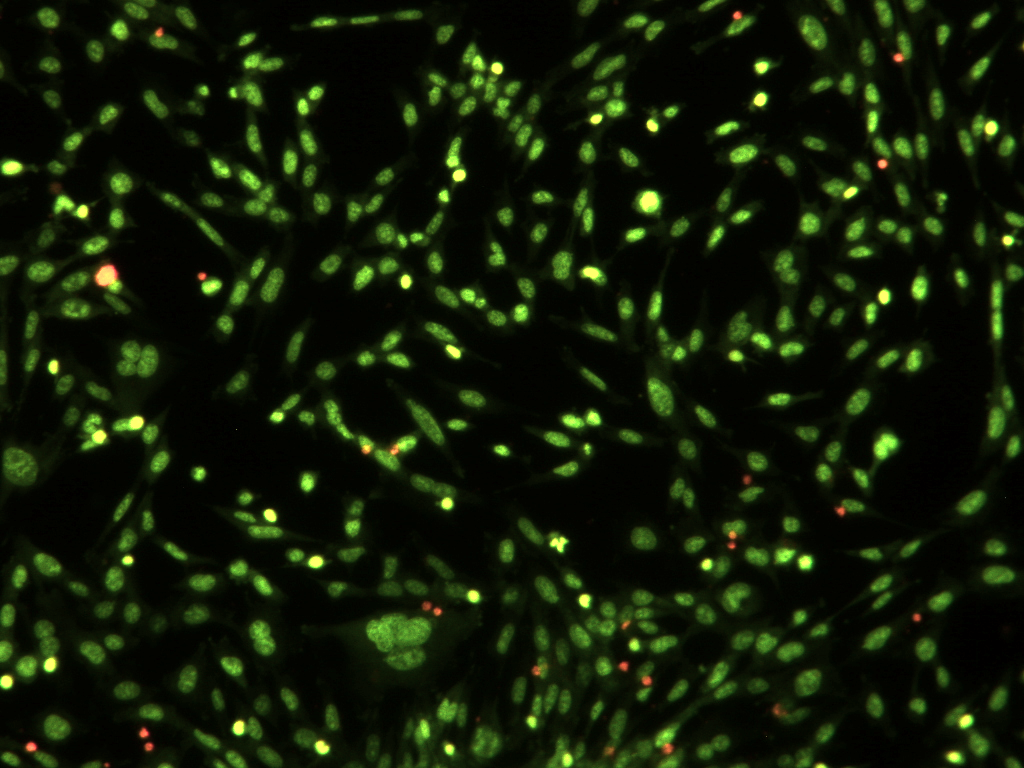

Supplement: S4 File — (ZIP) [file pone.0208866.s004.zip › S4_File/pone.0009826 EOC Replication Data 2018 (2 of 4)/wt cnt 0008_001.tif]

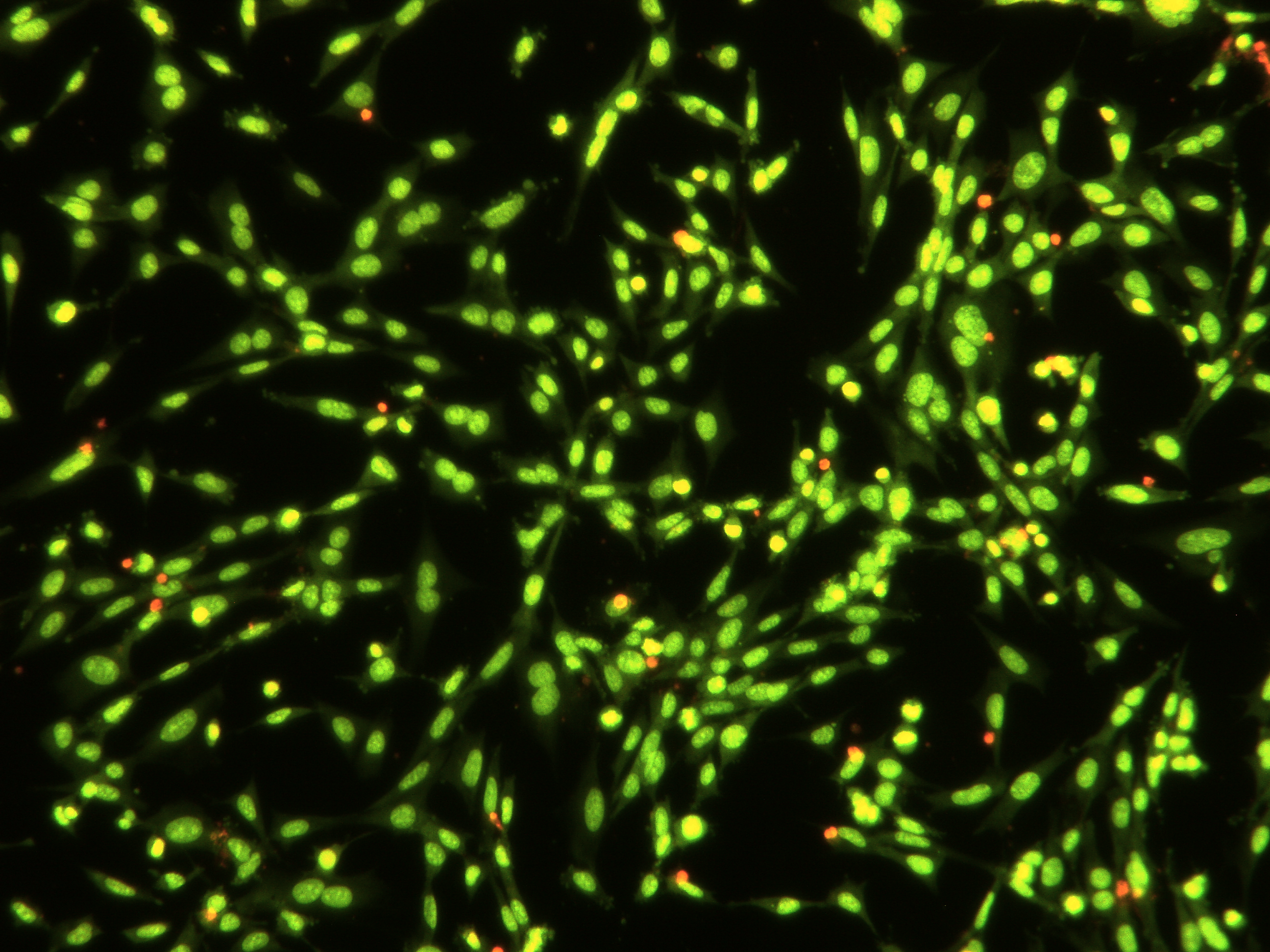

Supplement: S4 File — (ZIP) [file pone.0208866.s004.zip › S4_File/pone.0009826 EOC Replication Data 2018 (2 of 4)/wt cnt 0009.tif]

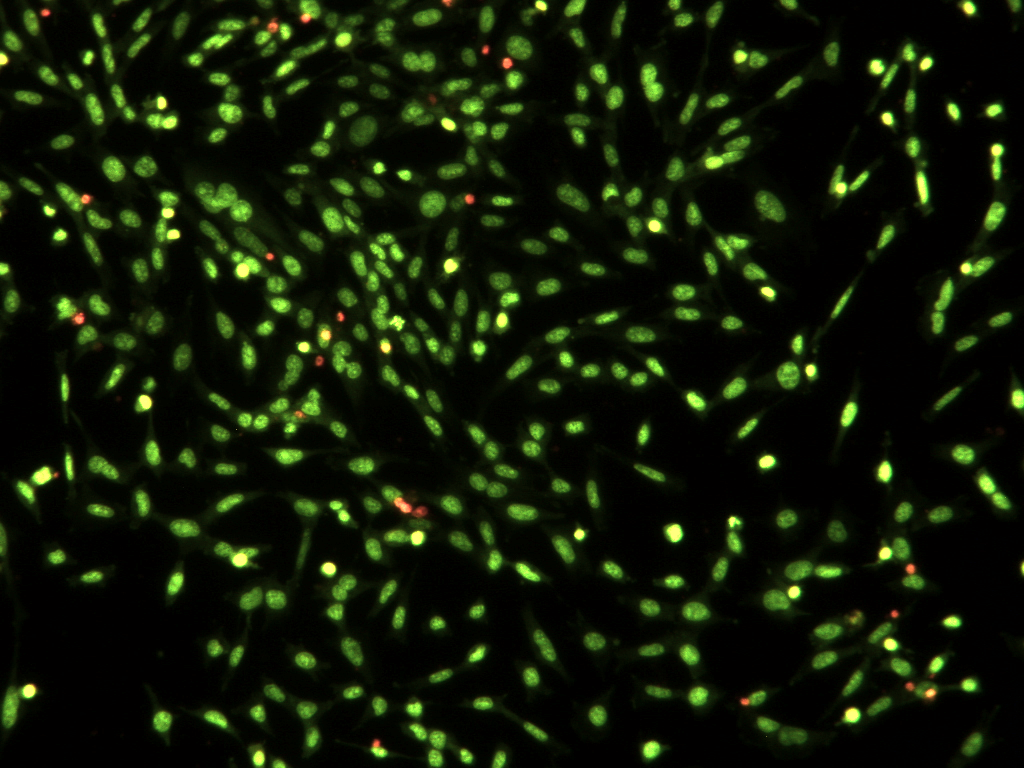

Supplement: S4 File — (ZIP) [file pone.0208866.s004.zip › S4_File/pone.0009826 EOC Replication Data 2018 (2 of 4)/wt cnt 0009_001.tif]

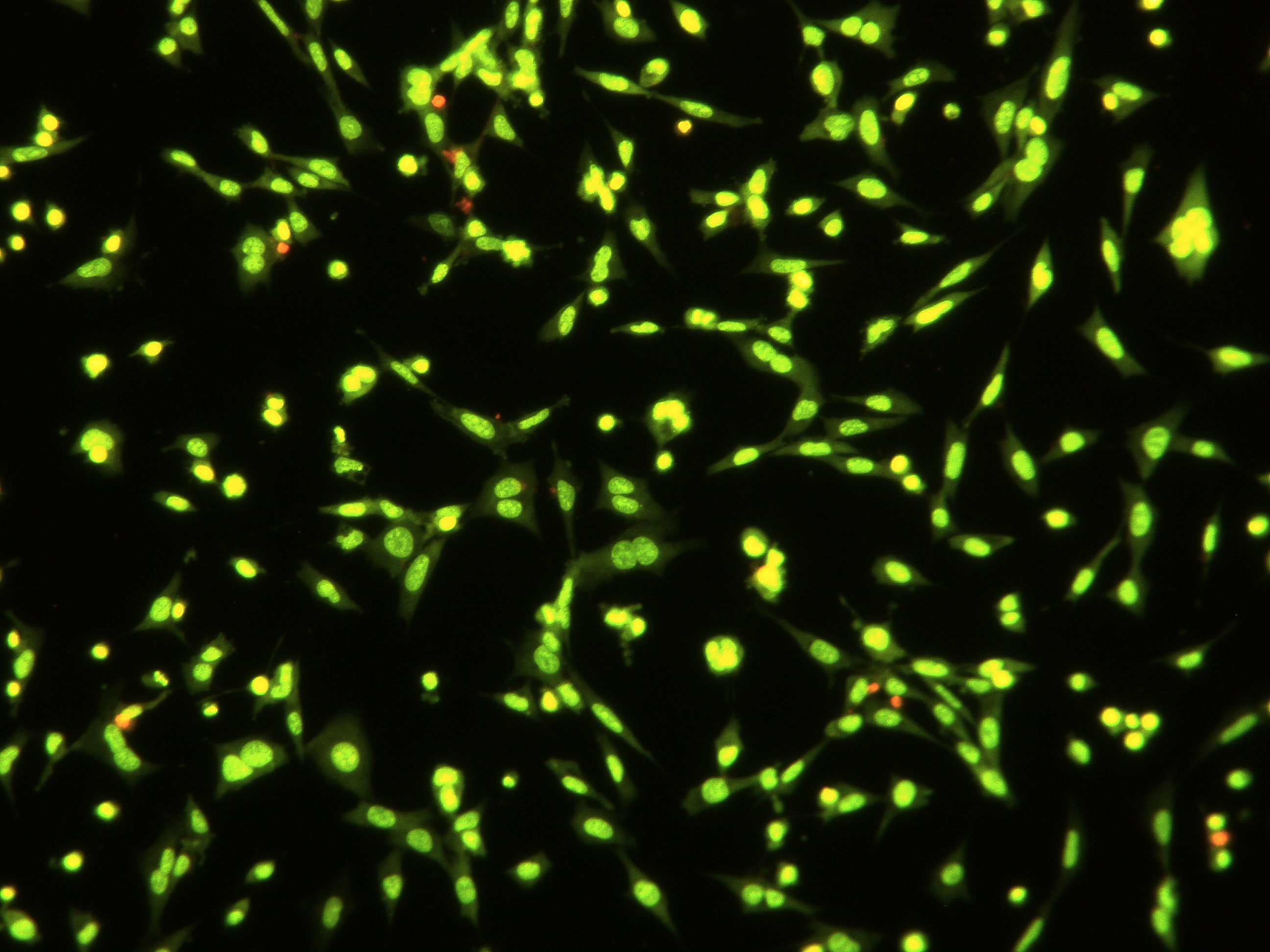

Supplement: S4 File — (ZIP) [file pone.0208866.s004.zip › S4_File/pone.0009826 EOC Replication Data 2018 (2 of 4)/wt cnt 0010.tif]

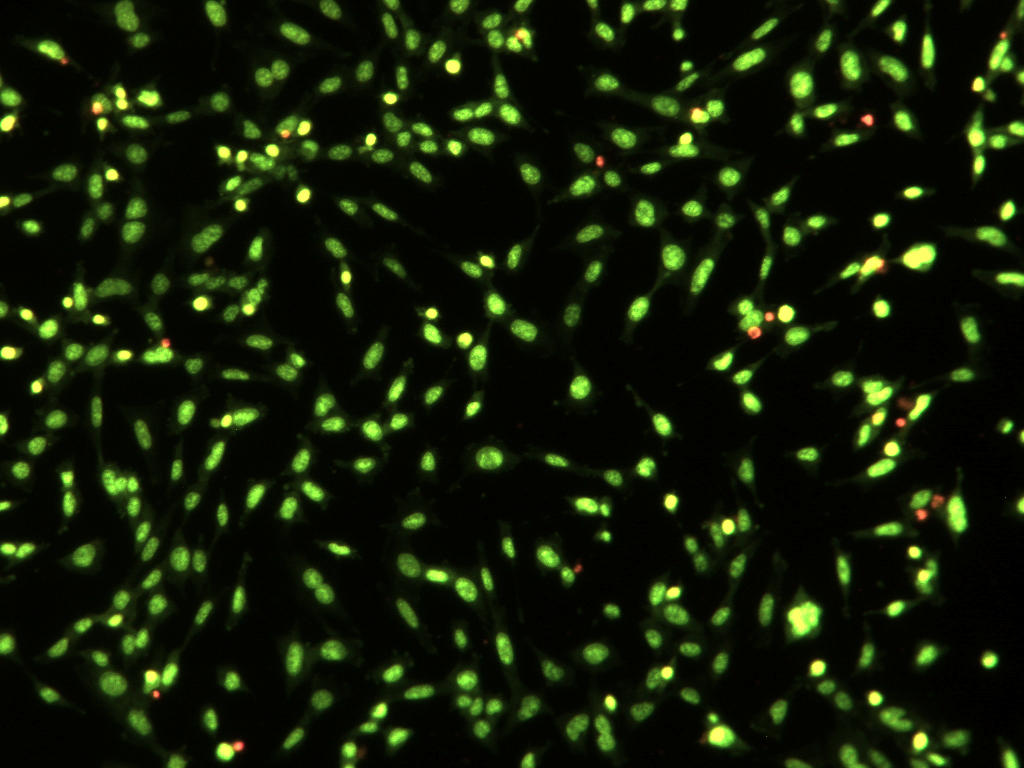

Supplement: S4 File — (ZIP) [file pone.0208866.s004.zip › S4_File/pone.0009826 EOC Replication Data 2018 (2 of 4)/wt cnt 0010_001.tif]

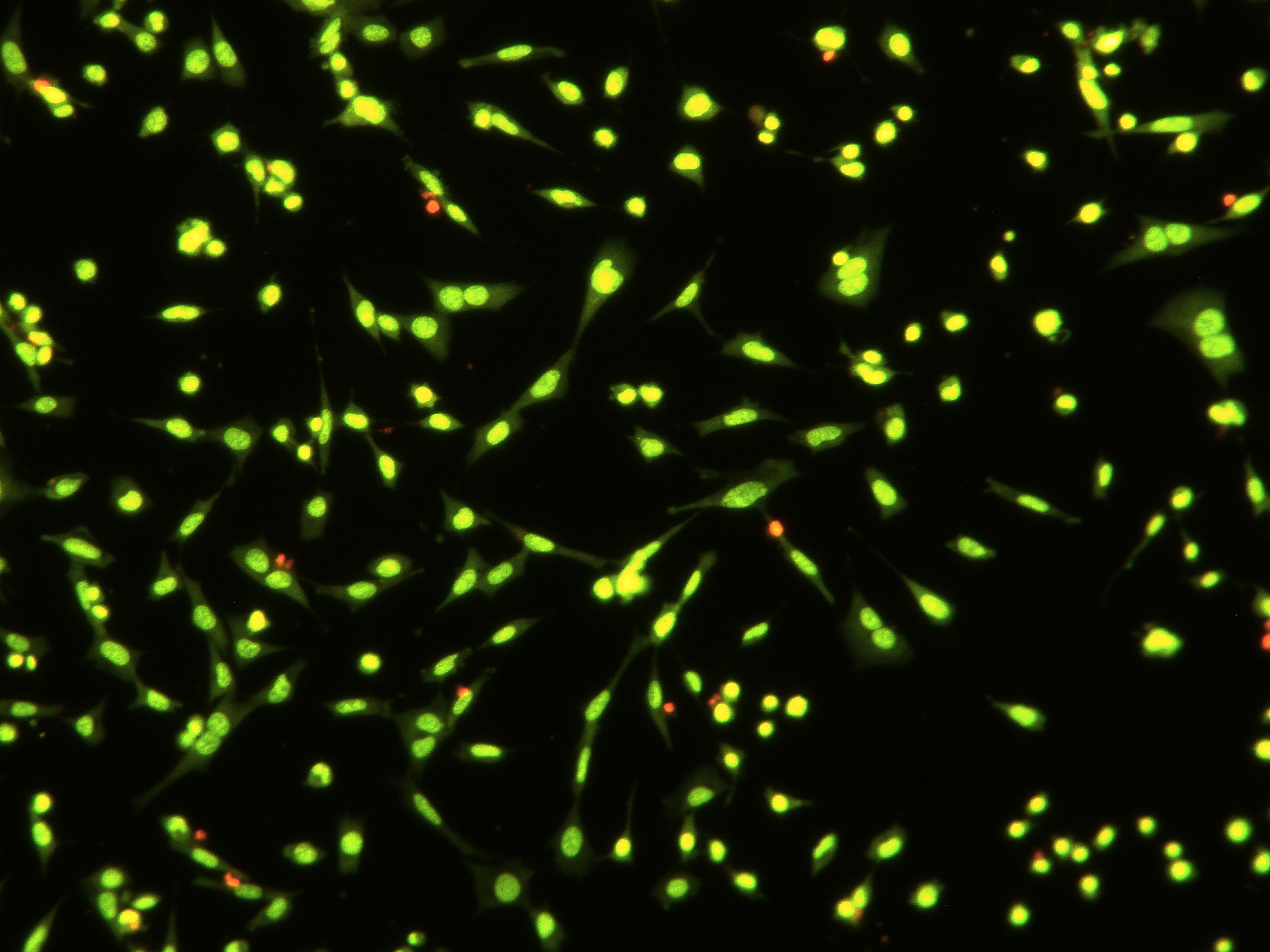

Supplement: S4 File — (ZIP) [file pone.0208866.s004.zip › S4_File/pone.0009826 EOC Replication Data 2018 (2 of 4)/wt cnt 0011.tif]

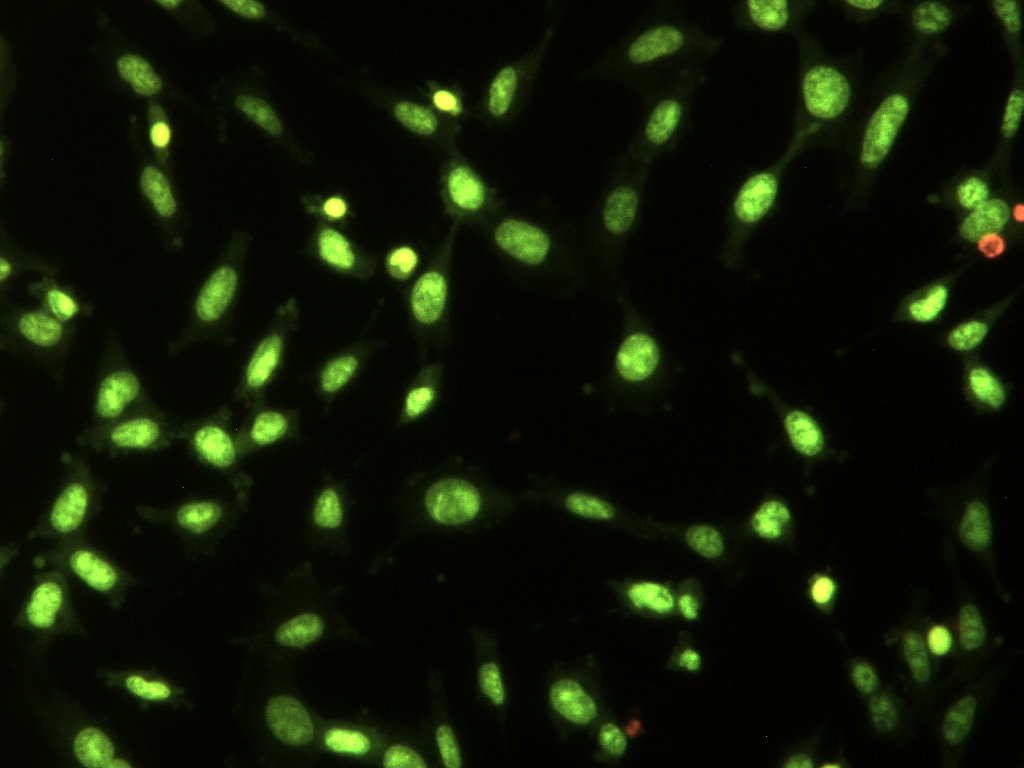

Supplement: S4 File — (ZIP) [file pone.0208866.s004.zip › S4_File/pone.0009826 EOC Replication Data 2018 (2 of 4)/wt cnt 0011_001.tif]

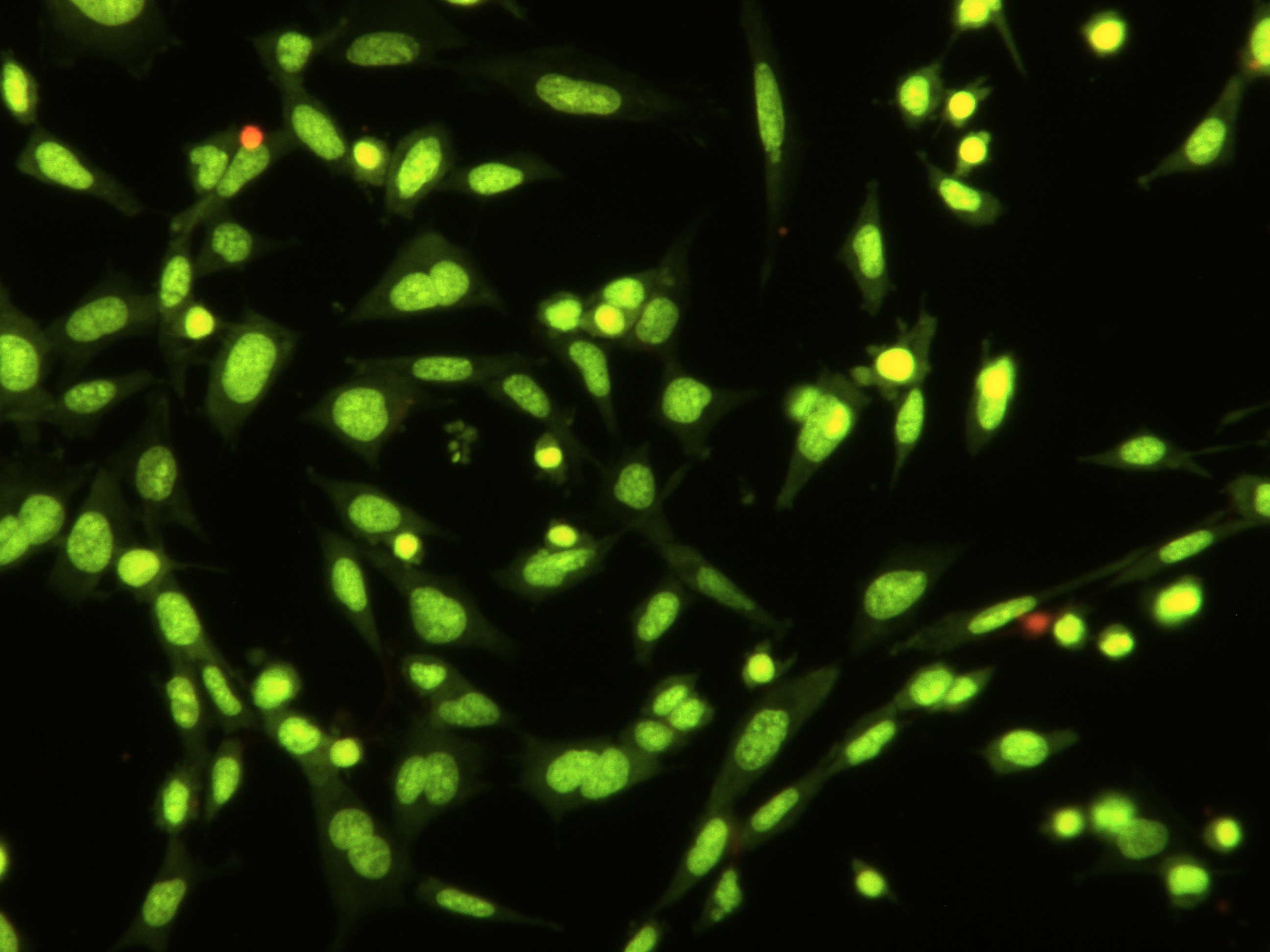

Supplement: S5 File — (ZIP) [file pone.0208866.s005.zip › S5_File/pone.0009826 EOC Replication Data 2018 (3 of 4)/wt cnt 0012.tif]

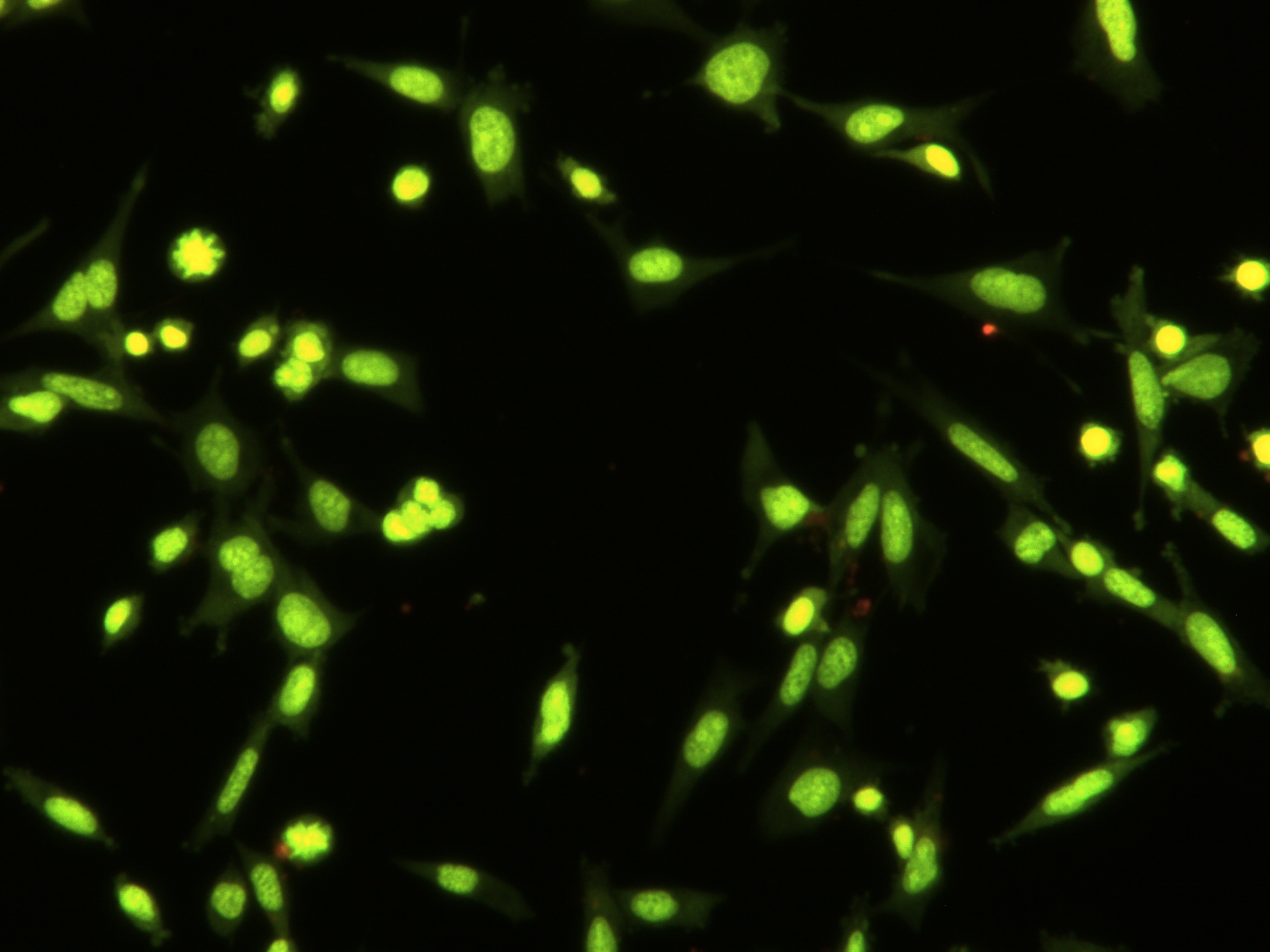

Supplement: S5 File — (ZIP) [file pone.0208866.s005.zip › S5_File/pone.0009826 EOC Replication Data 2018 (3 of 4)/wt cnt 0013.tif]

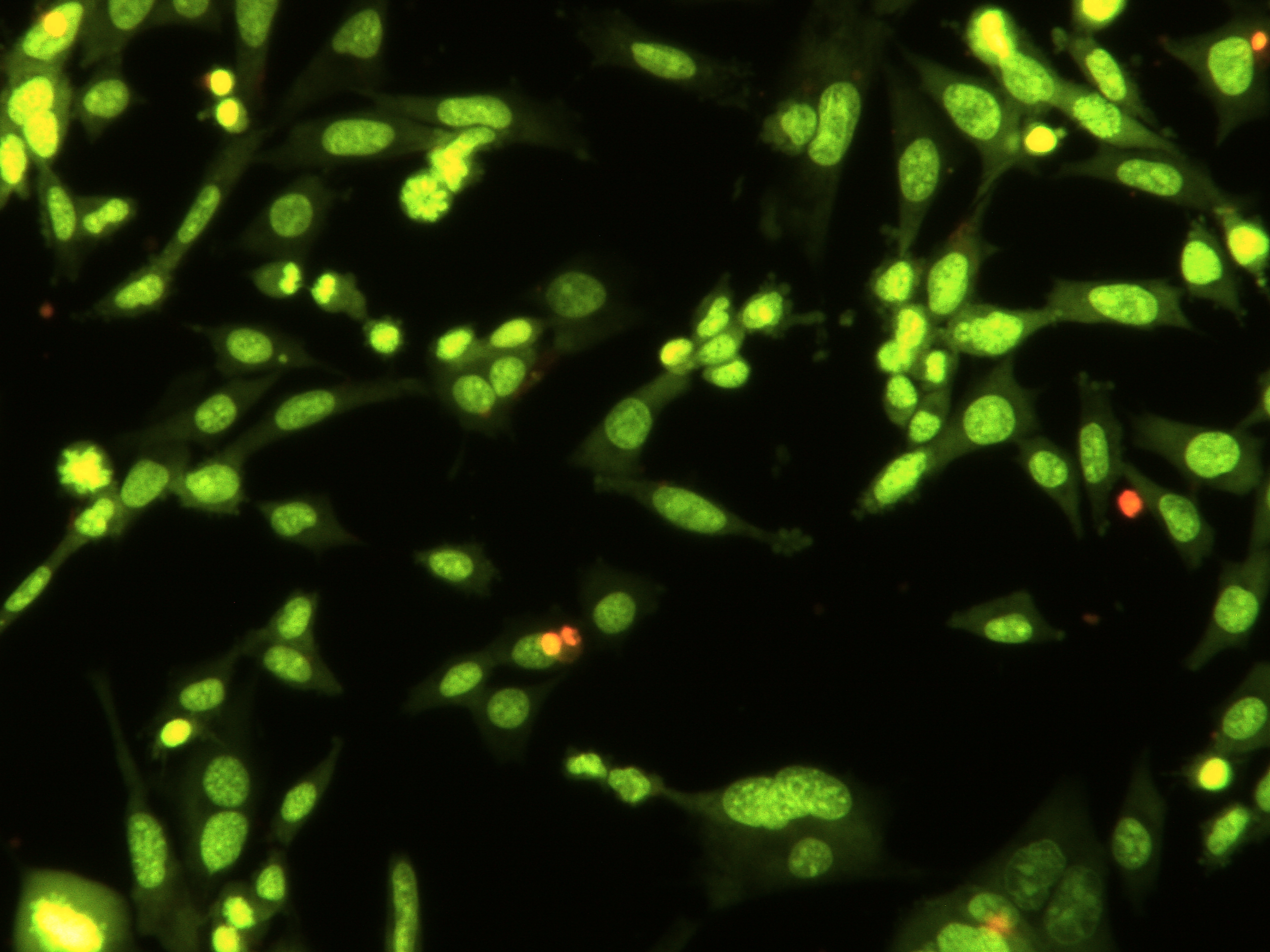

Supplement: S5 File — (ZIP) [file pone.0208866.s005.zip › S5_File/pone.0009826 EOC Replication Data 2018 (3 of 4)/wt cnt 0014.tif]

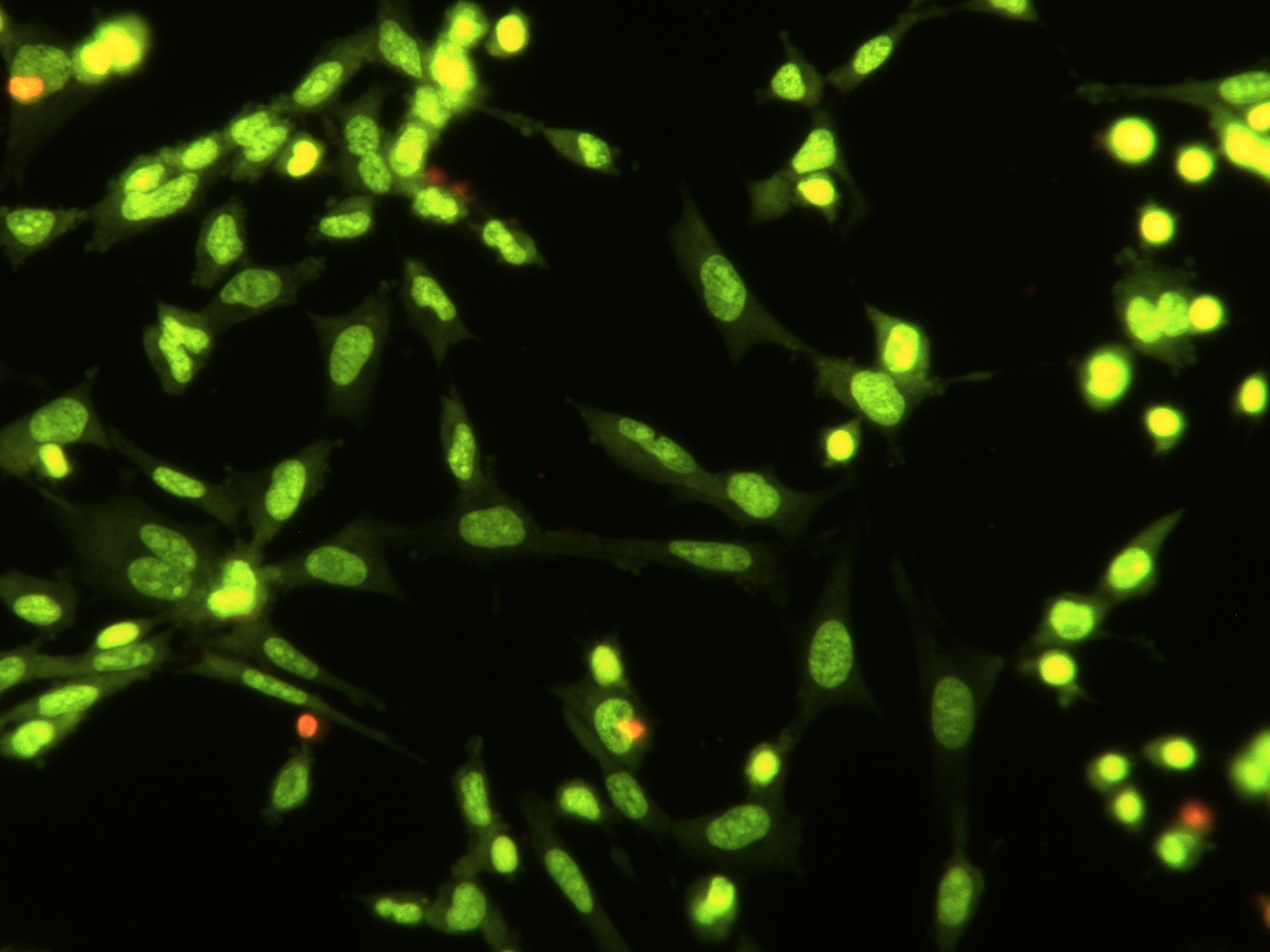

Supplement: S5 File — (ZIP) [file pone.0208866.s005.zip › S5_File/pone.0009826 EOC Replication Data 2018 (3 of 4)/wt cnt 0015.tif]

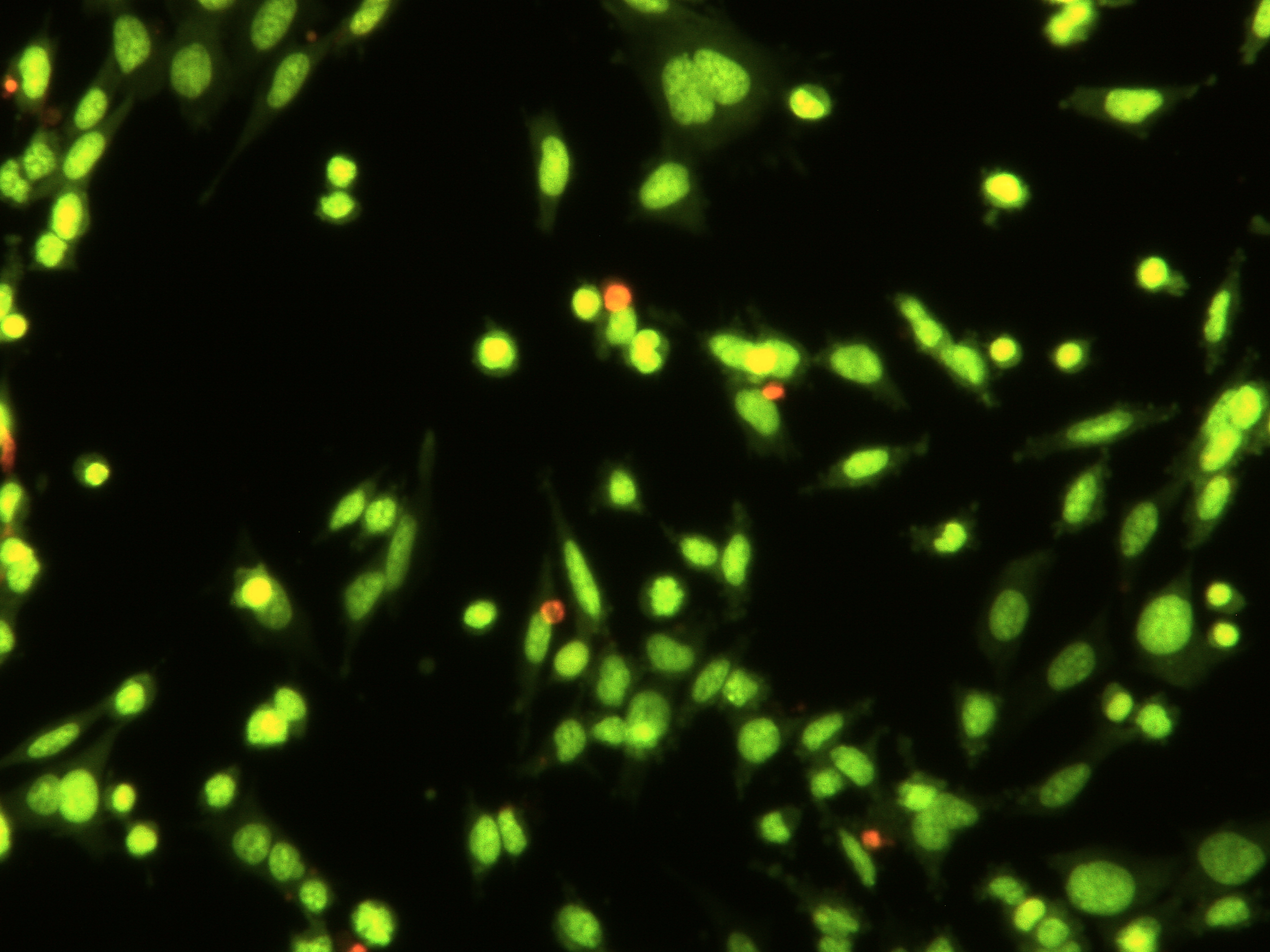

Supplement: S5 File — (ZIP) [file pone.0208866.s005.zip › S5_File/pone.0009826 EOC Replication Data 2018 (3 of 4)/wt cnt 0016.tif]

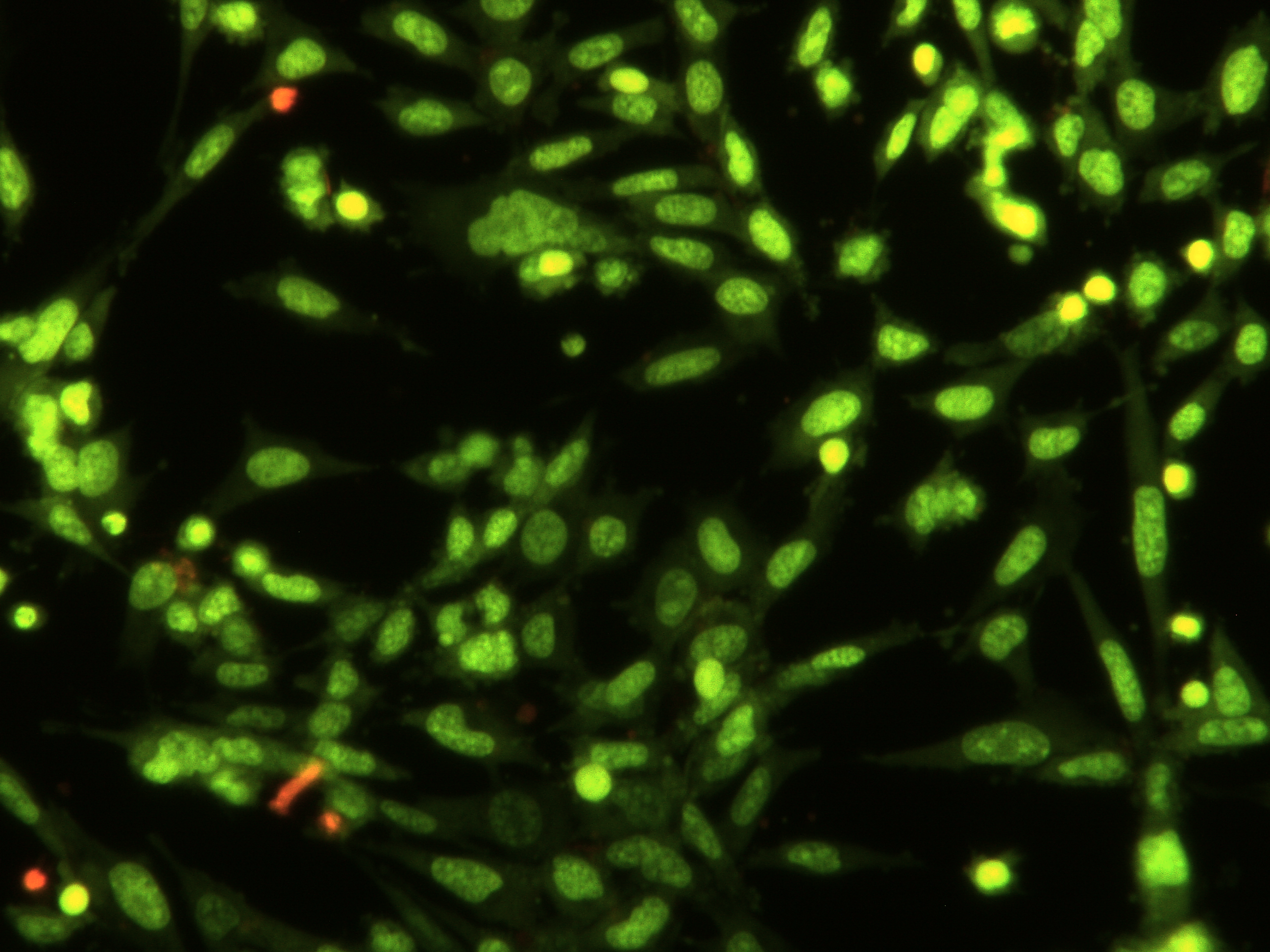

Supplement: S5 File — (ZIP) [file pone.0208866.s005.zip › S5_File/pone.0009826 EOC Replication Data 2018 (3 of 4)/wt cnt 0017.tif]

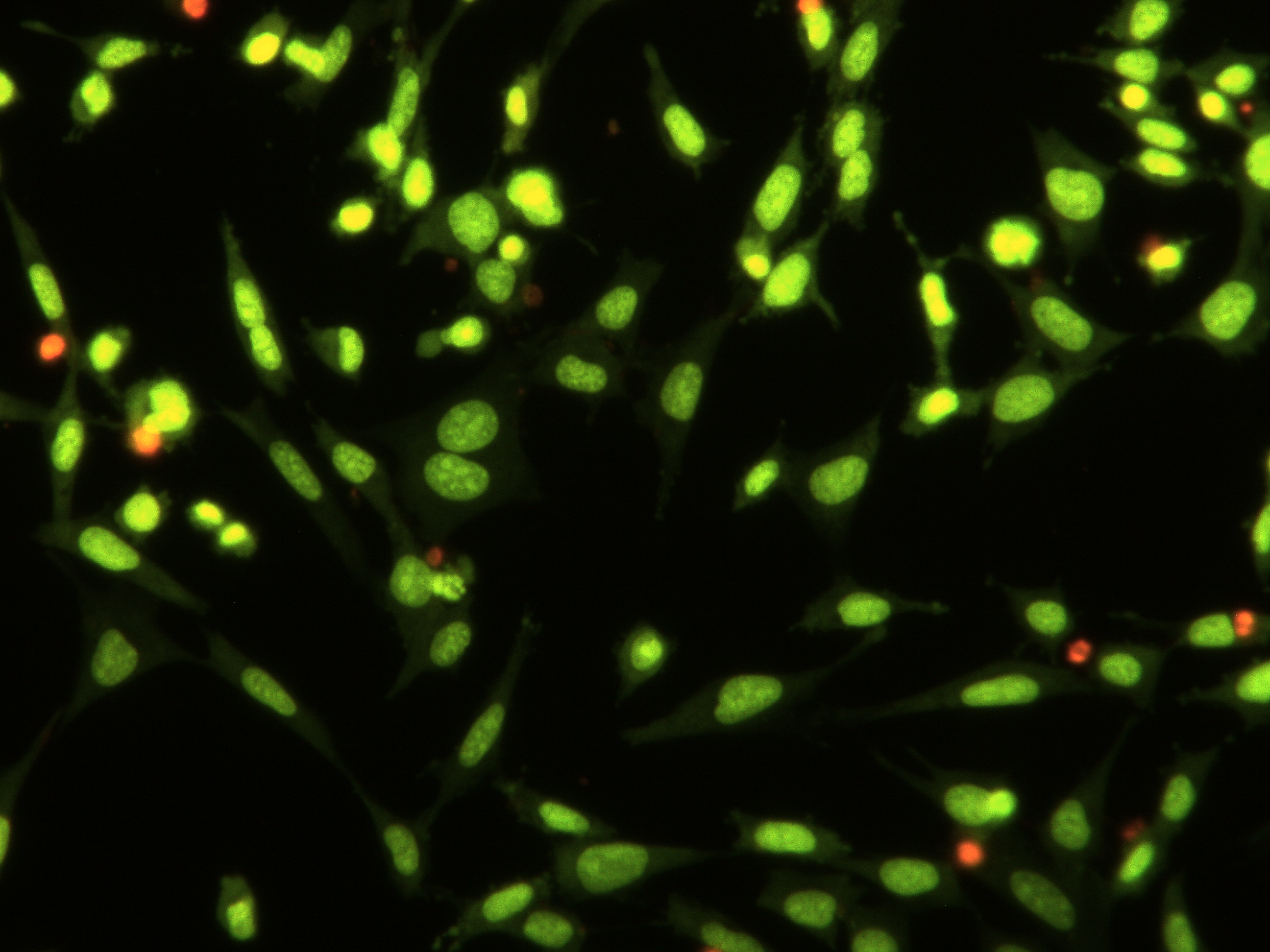

Supplement: S5 File — (ZIP) [file pone.0208866.s005.zip › S5_File/pone.0009826 EOC Replication Data 2018 (3 of 4)/wt cnt 0018.tif]

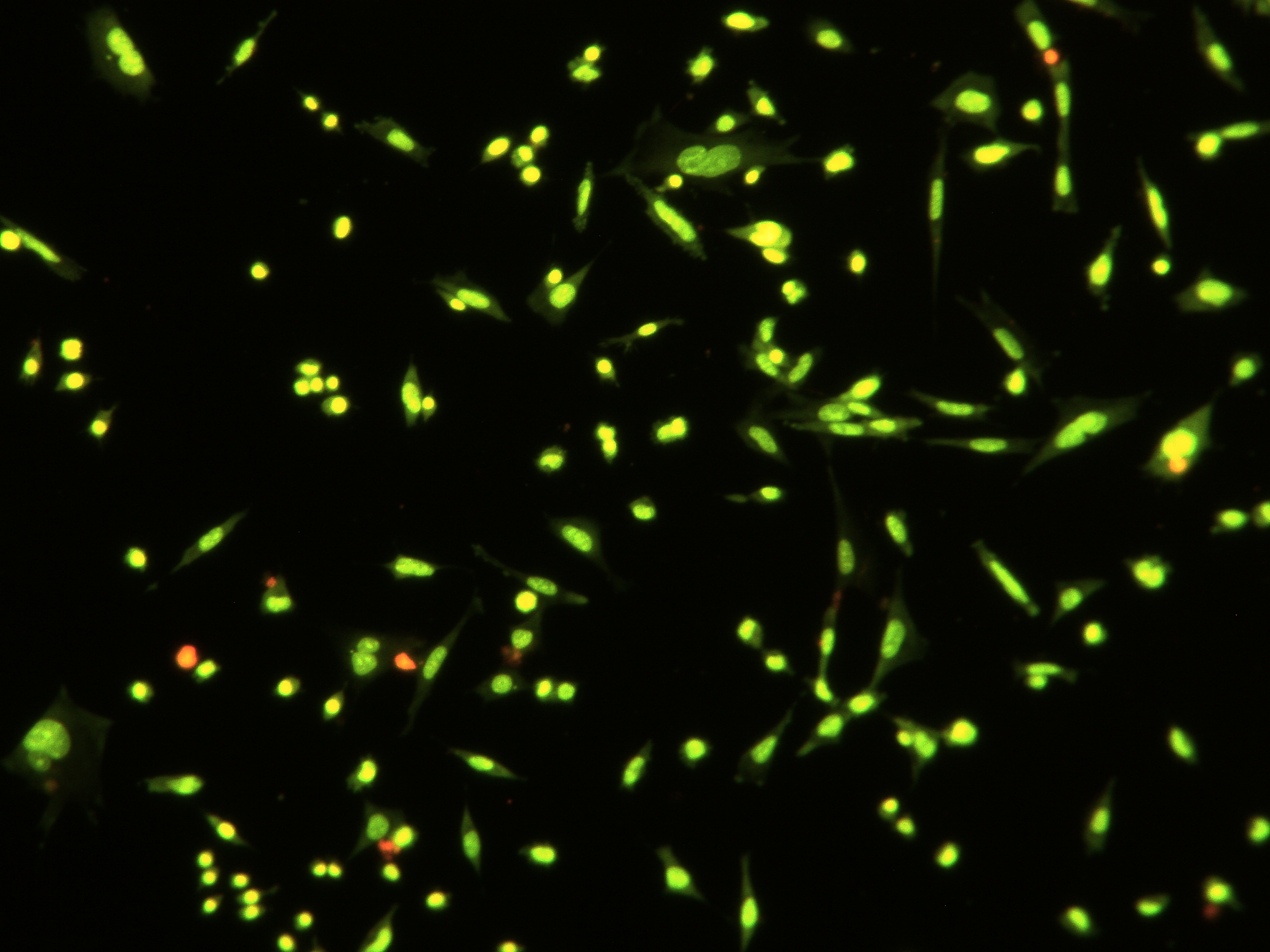

Supplement: S5 File — (ZIP) [file pone.0208866.s005.zip › S5_File/pone.0009826 EOC Replication Data 2018 (3 of 4)/wt cnt.tif]

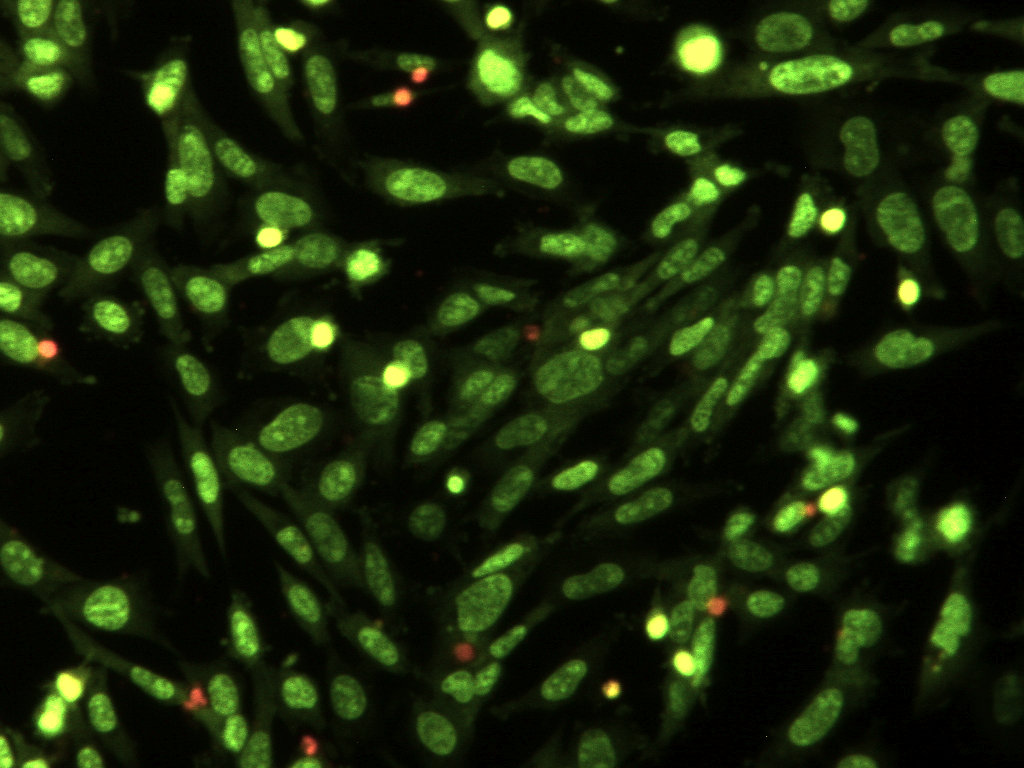

Supplement: S5 File — (ZIP) [file pone.0208866.s005.zip › S5_File/pone.0009826 EOC Replication Data 2018 (3 of 4)/wt cnt_001.tif]

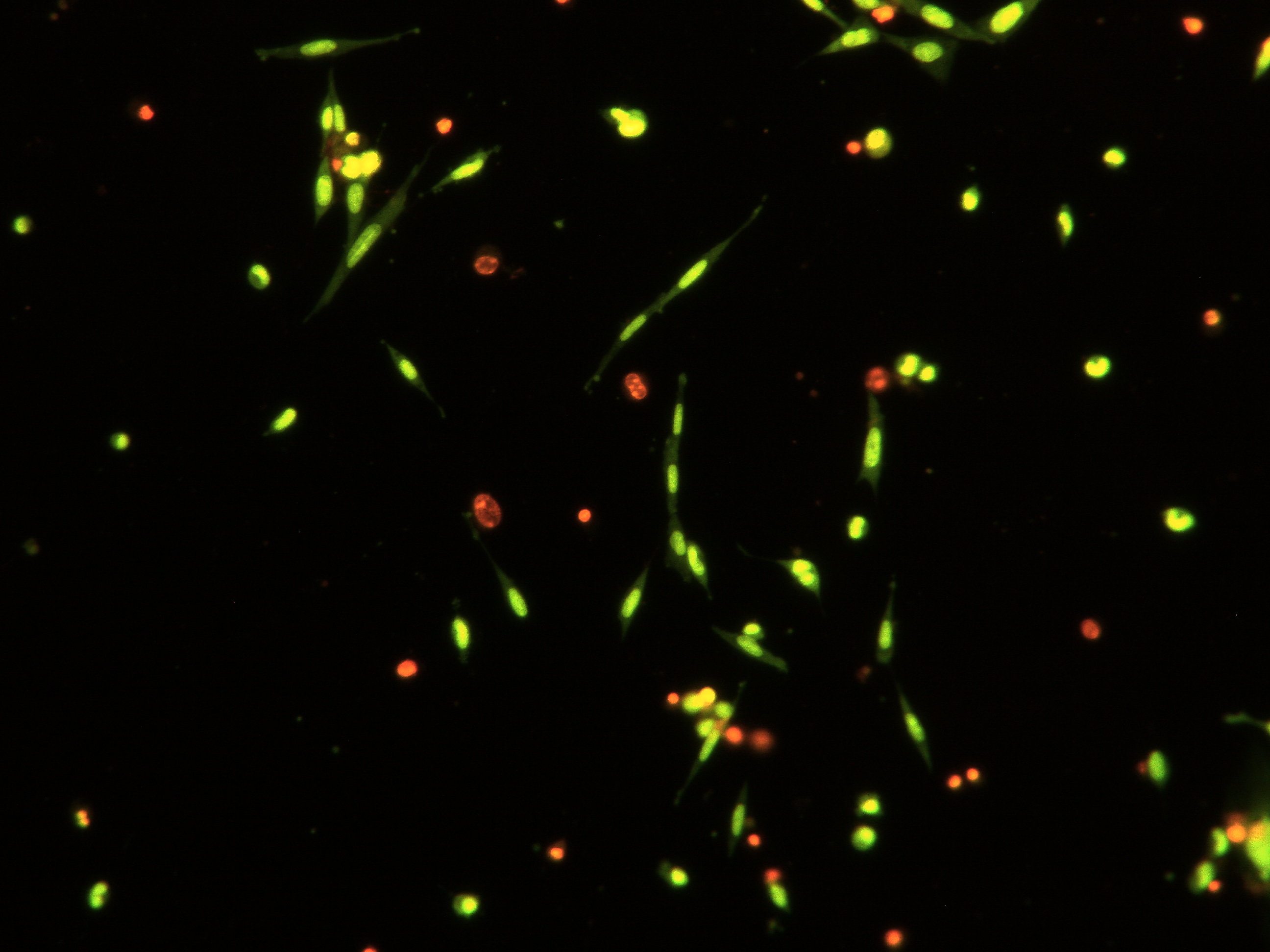

Supplement: S5 File — (ZIP) [file pone.0208866.s005.zip › S5_File/pone.0009826 EOC Replication Data 2018 (3 of 4)/wt ox200 0010.tif]

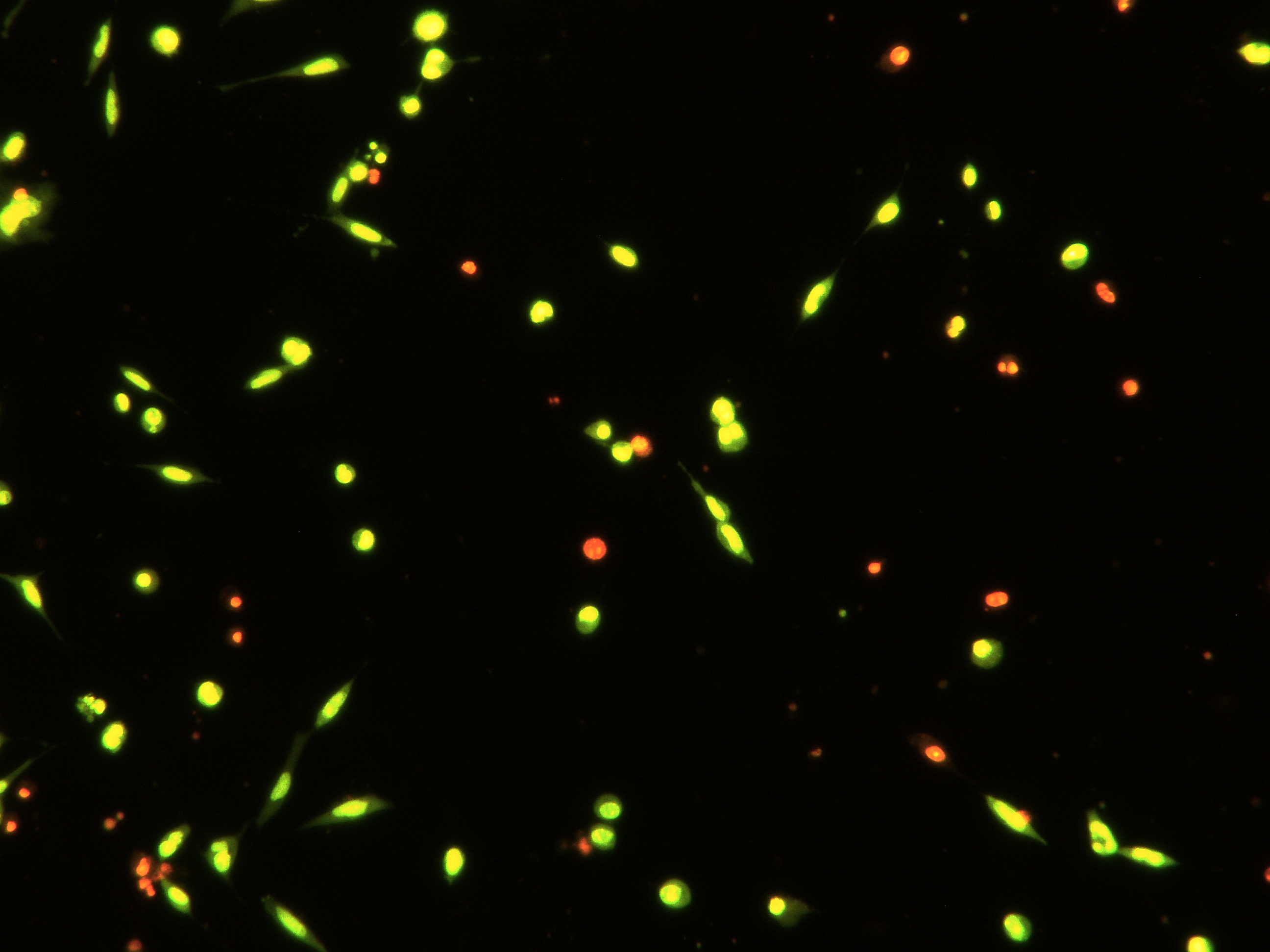

Supplement: S5 File — (ZIP) [file pone.0208866.s005.zip › S5_File/pone.0009826 EOC Replication Data 2018 (3 of 4)/wt ox200 0011.tif]

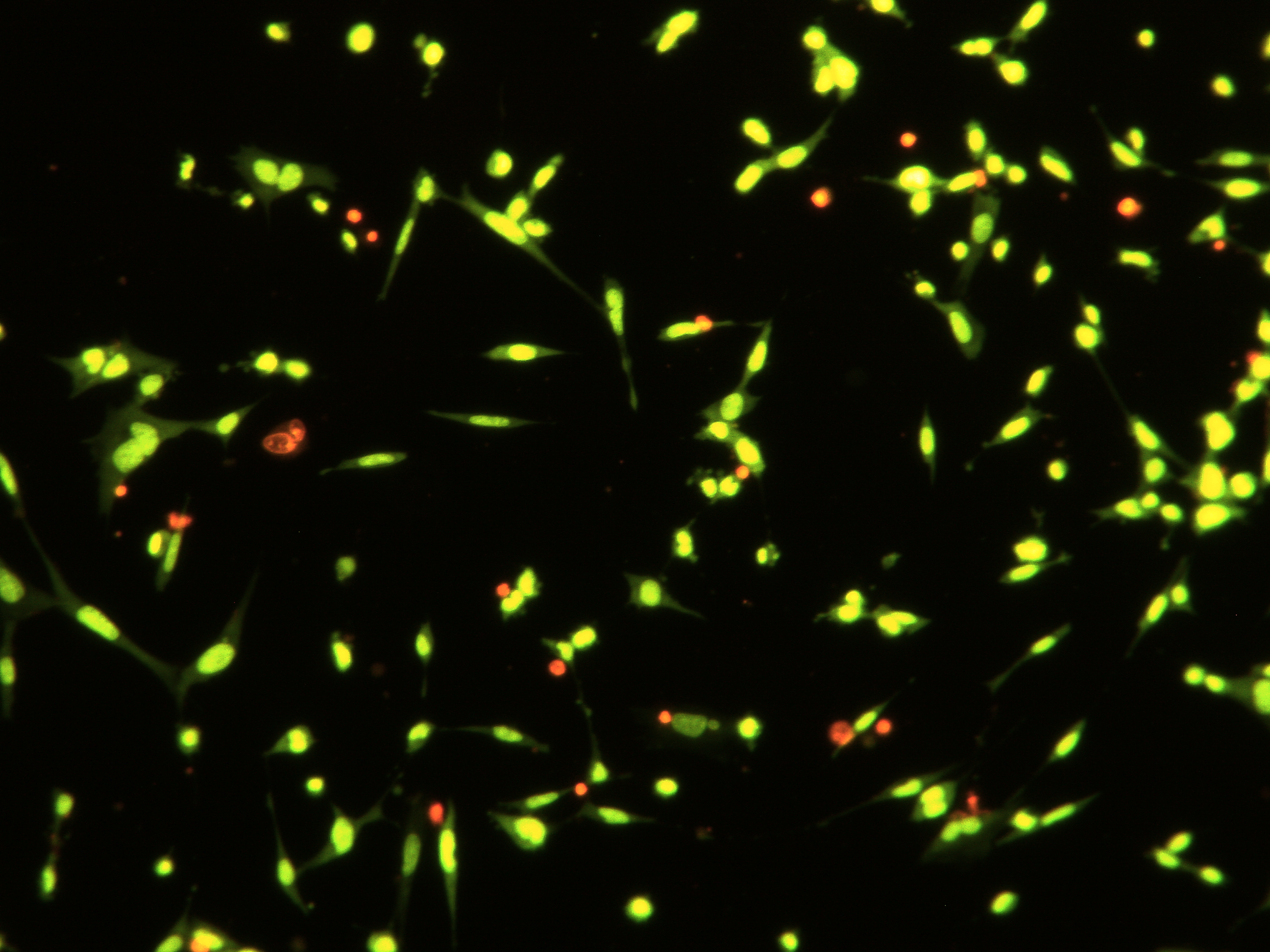

Supplement: S5 File — (ZIP) [file pone.0208866.s005.zip › S5_File/pone.0009826 EOC Replication Data 2018 (3 of 4)/wt ox200 0012.tif]

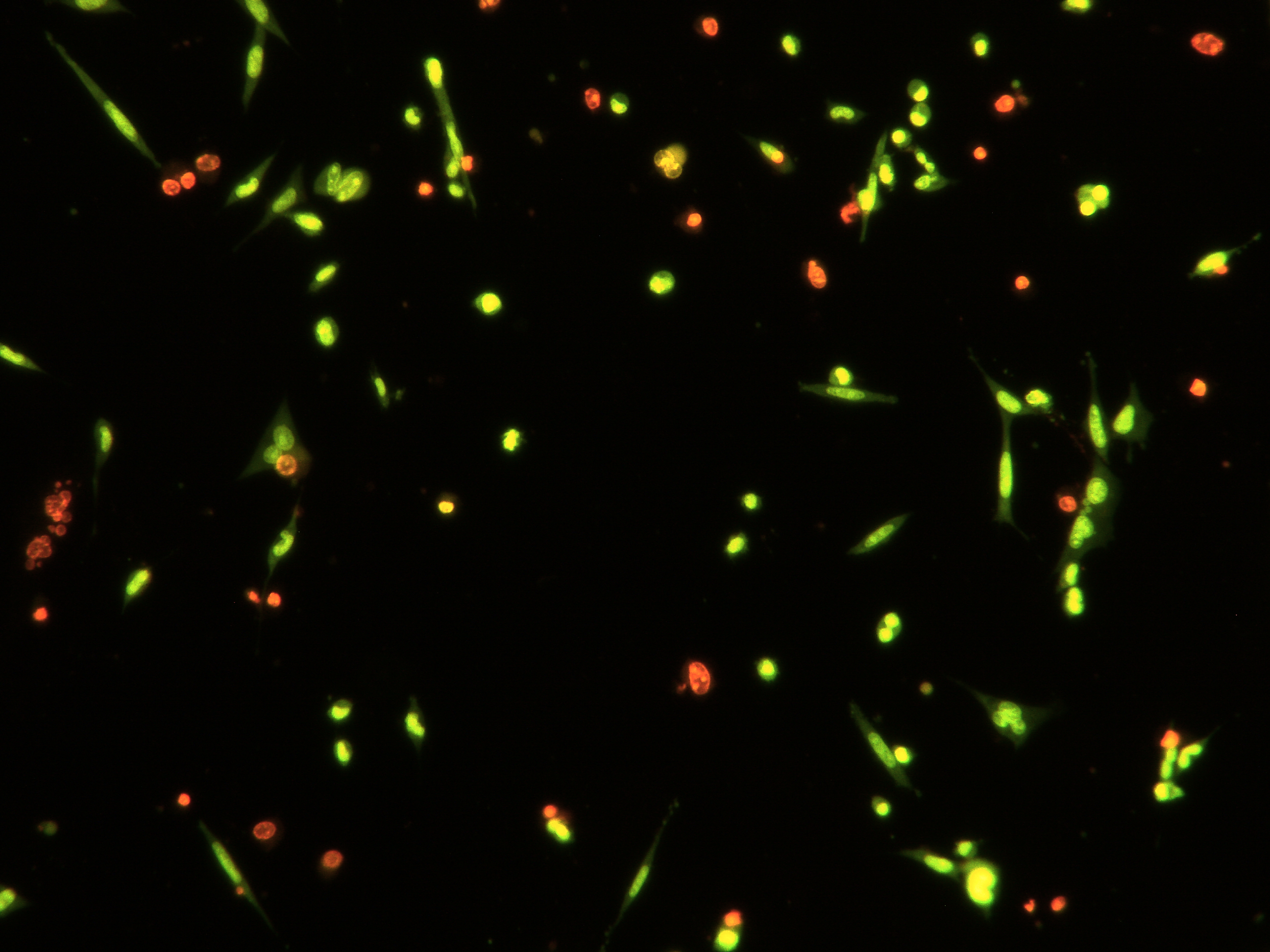

Supplement: S5 File — (ZIP) [file pone.0208866.s005.zip › S5_File/pone.0009826 EOC Replication Data 2018 (3 of 4)/wt ox200 002.tif]

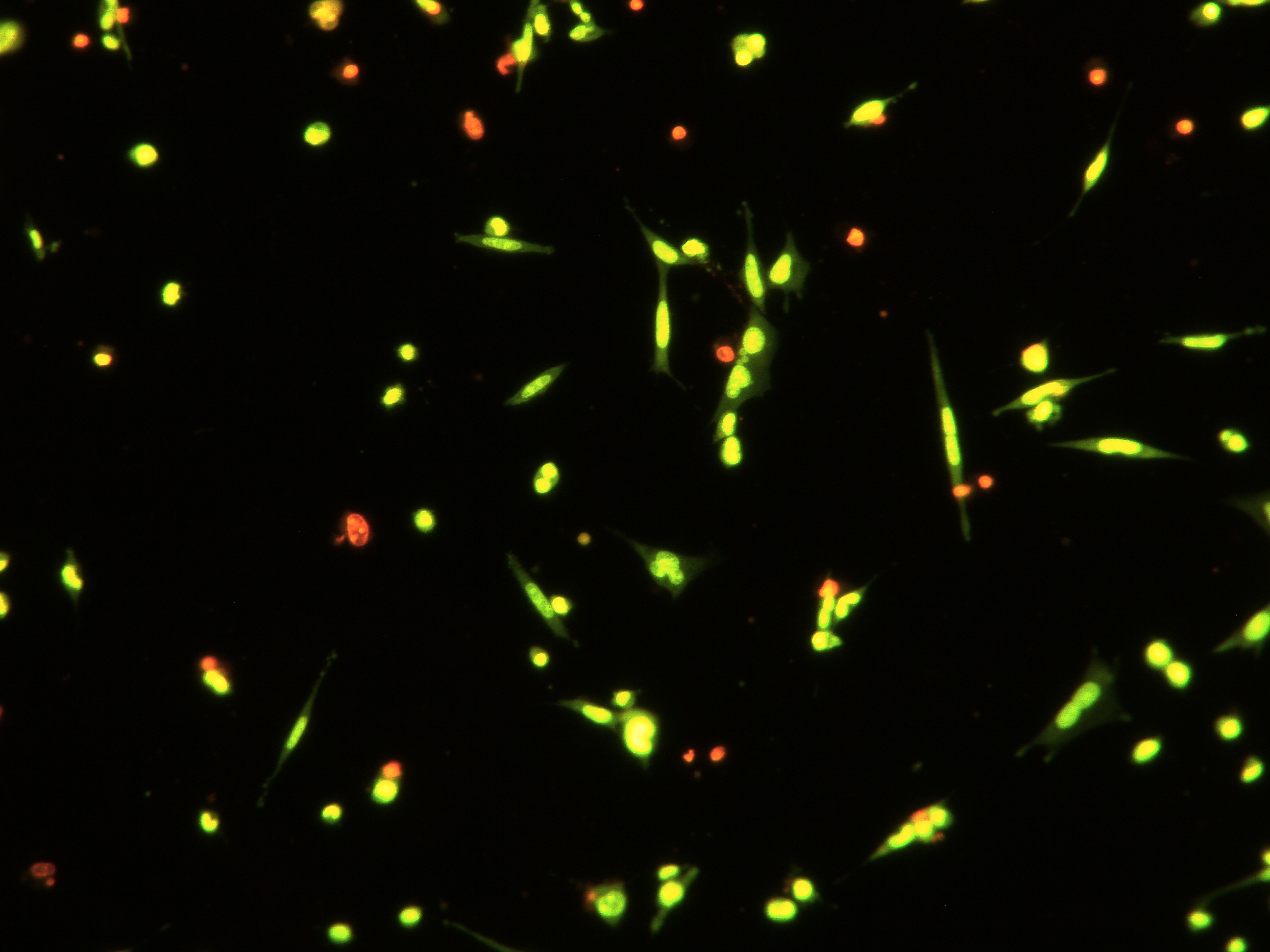

Supplement: S5 File — (ZIP) [file pone.0208866.s005.zip › S5_File/pone.0009826 EOC Replication Data 2018 (3 of 4)/wt ox200 003.tif]

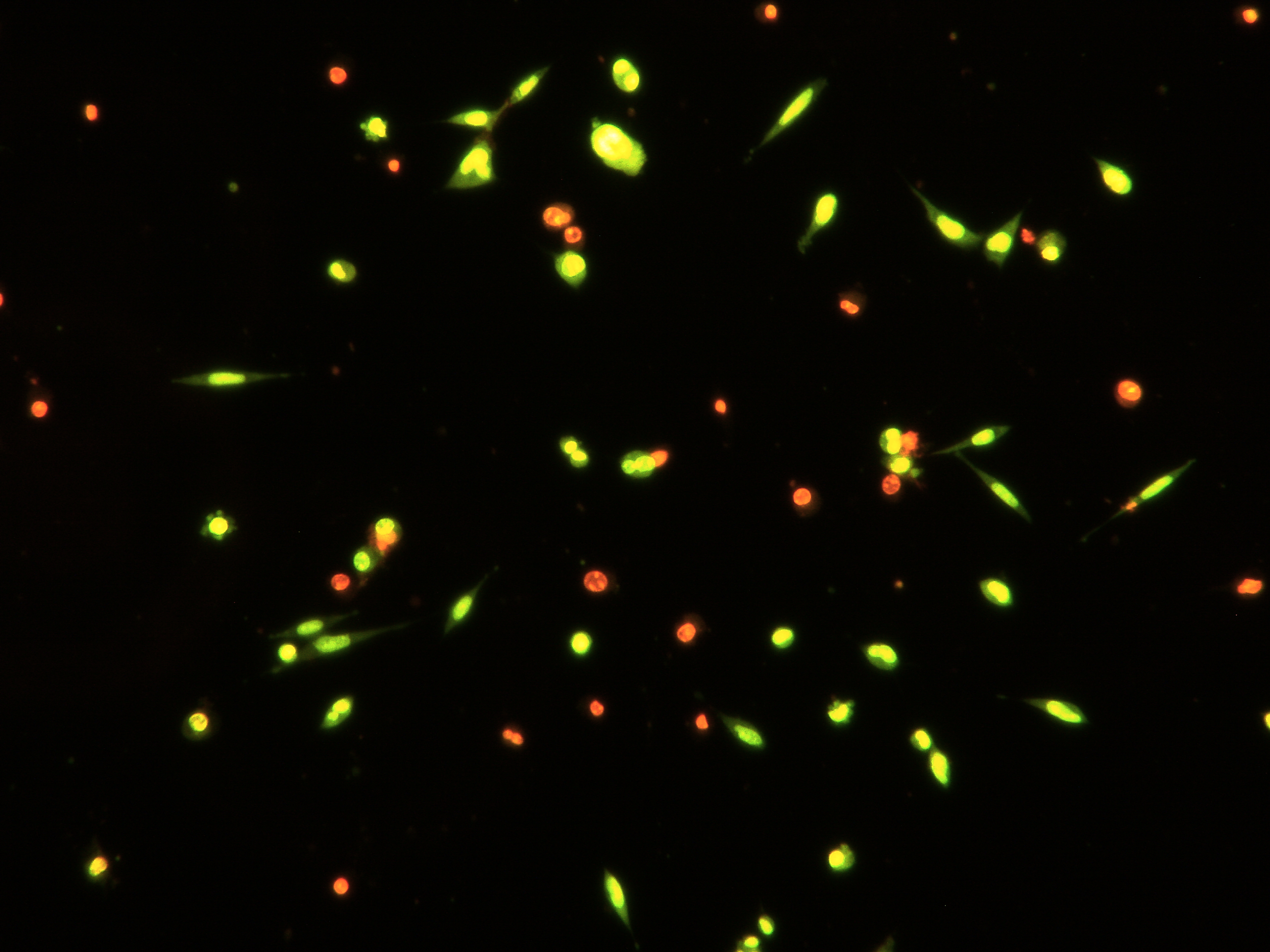

Supplement: S5 File — (ZIP) [file pone.0208866.s005.zip › S5_File/pone.0009826 EOC Replication Data 2018 (3 of 4)/wt ox200 004.tif]

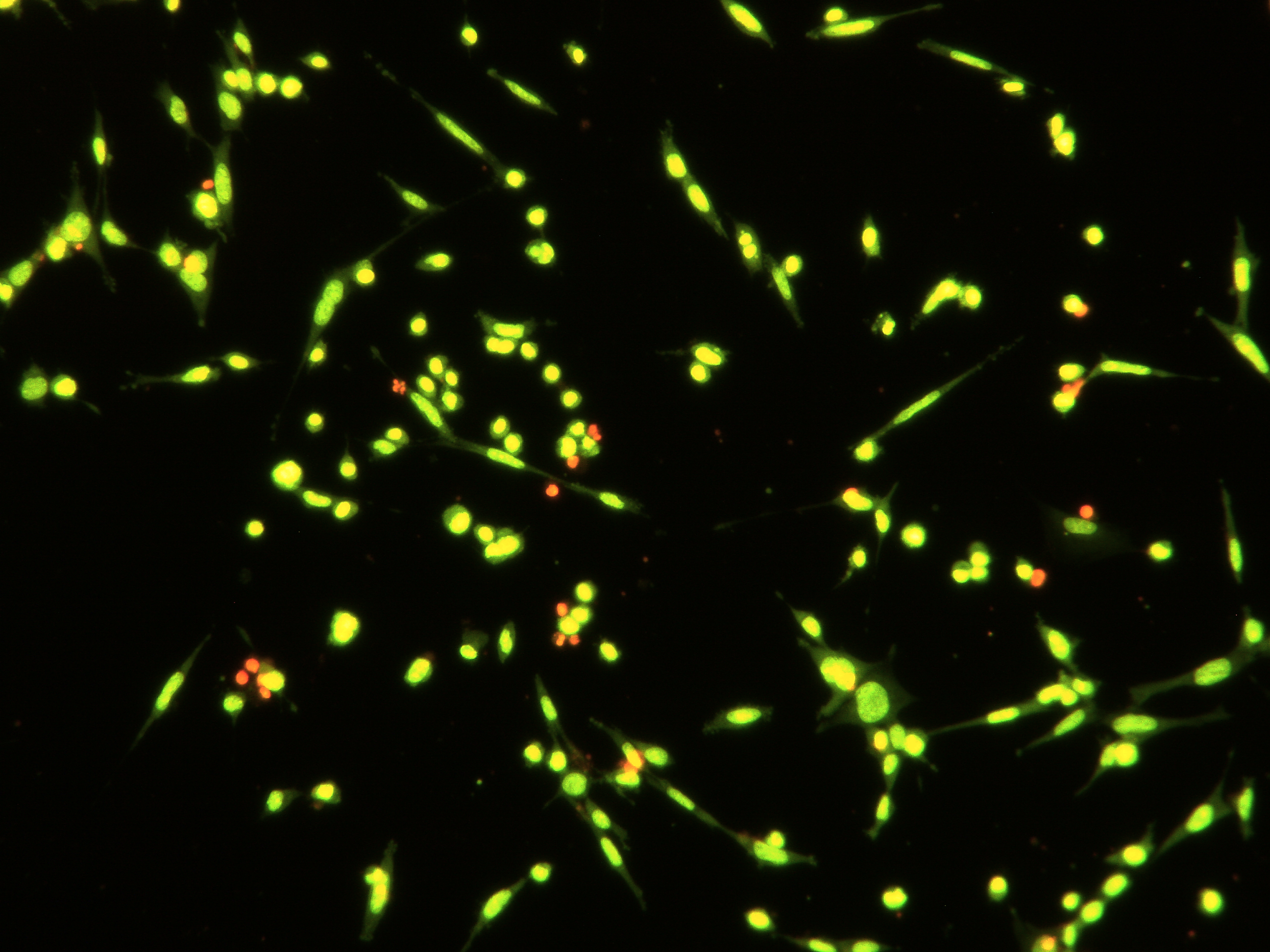

Supplement: S5 File — (ZIP) [file pone.0208866.s005.zip › S5_File/pone.0009826 EOC Replication Data 2018 (3 of 4)/wt ox200 005.tif]

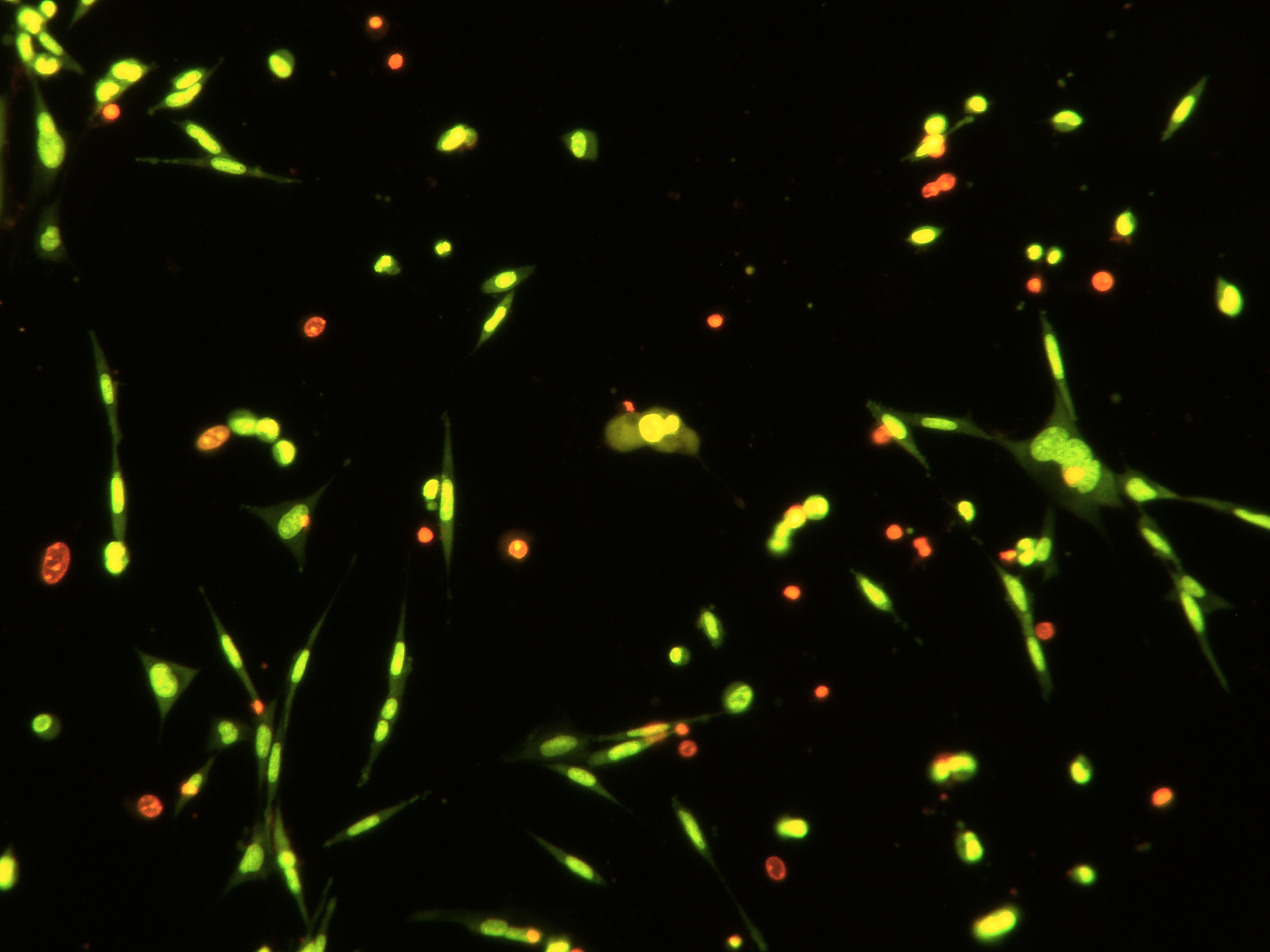

Supplement: S5 File — (ZIP) [file pone.0208866.s005.zip › S5_File/pone.0009826 EOC Replication Data 2018 (3 of 4)/wt ox200 006.tif]

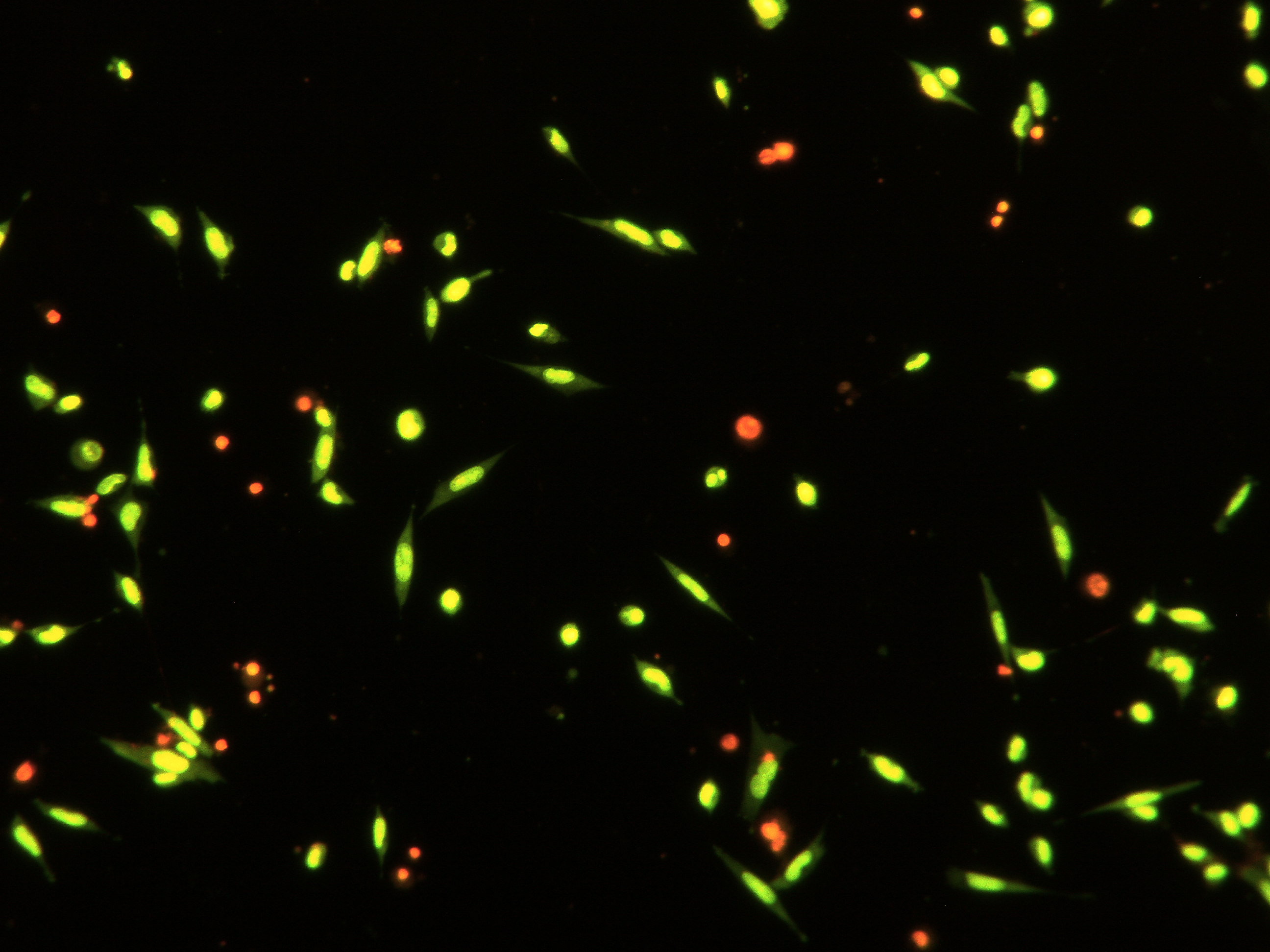

Supplement: S5 File — (ZIP) [file pone.0208866.s005.zip › S5_File/pone.0009826 EOC Replication Data 2018 (3 of 4)/wt ox200 007.tif]

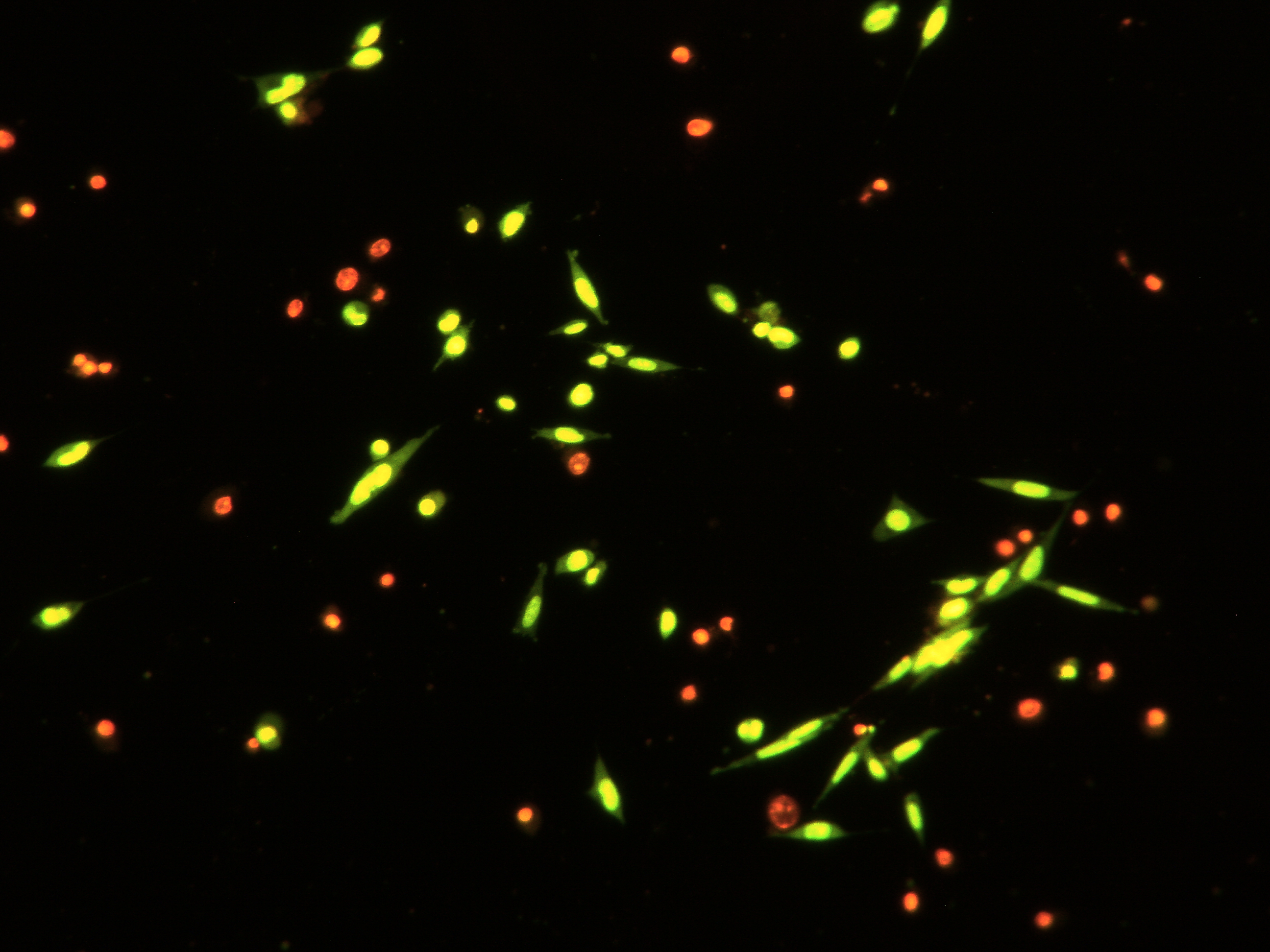

Supplement: S5 File — (ZIP) [file pone.0208866.s005.zip › S5_File/pone.0009826 EOC Replication Data 2018 (3 of 4)/wt ox200 008.tif]

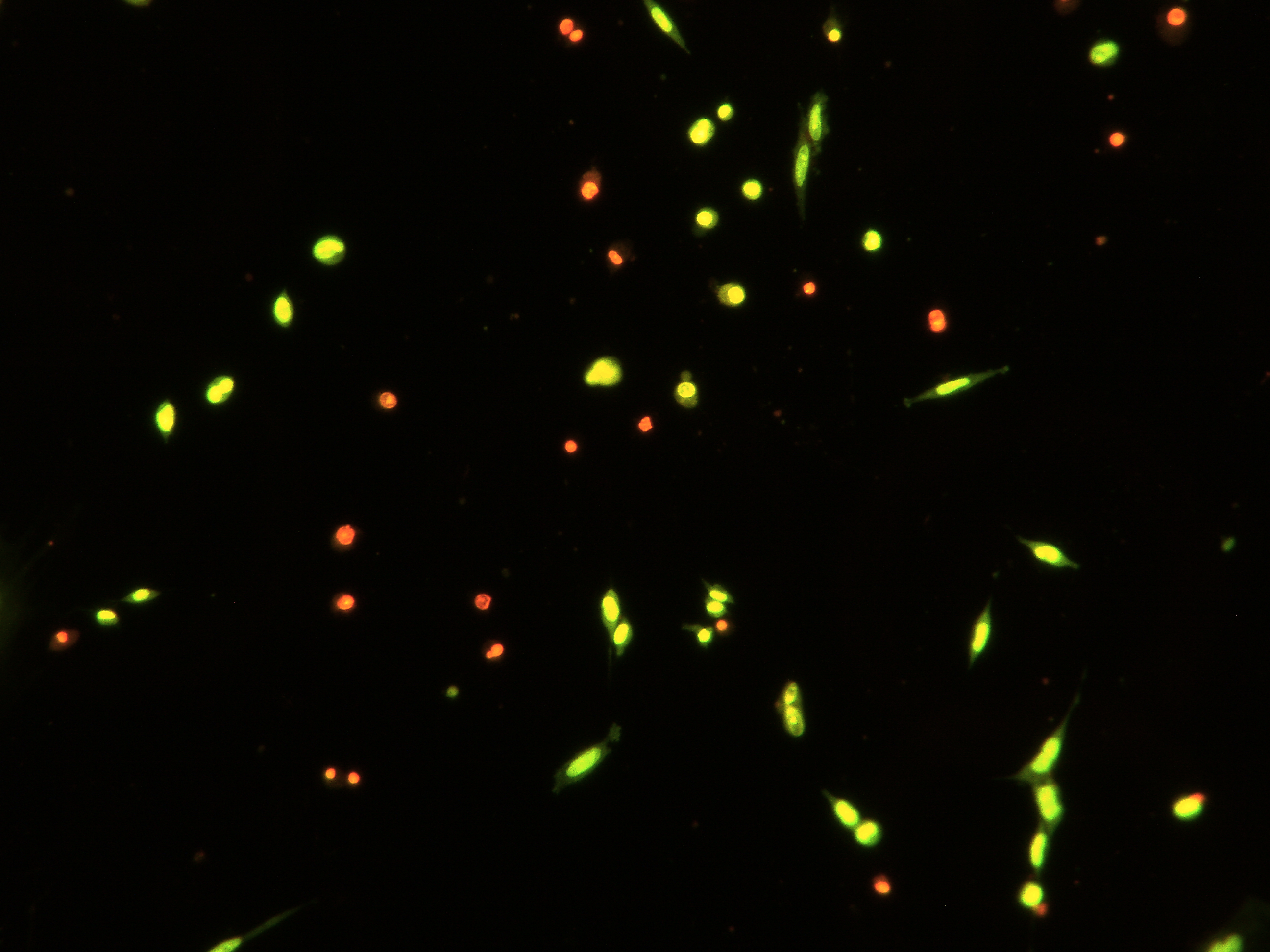

Supplement: S5 File — (ZIP) [file pone.0208866.s005.zip › S5_File/pone.0009826 EOC Replication Data 2018 (3 of 4)/wt ox200 009.tif]

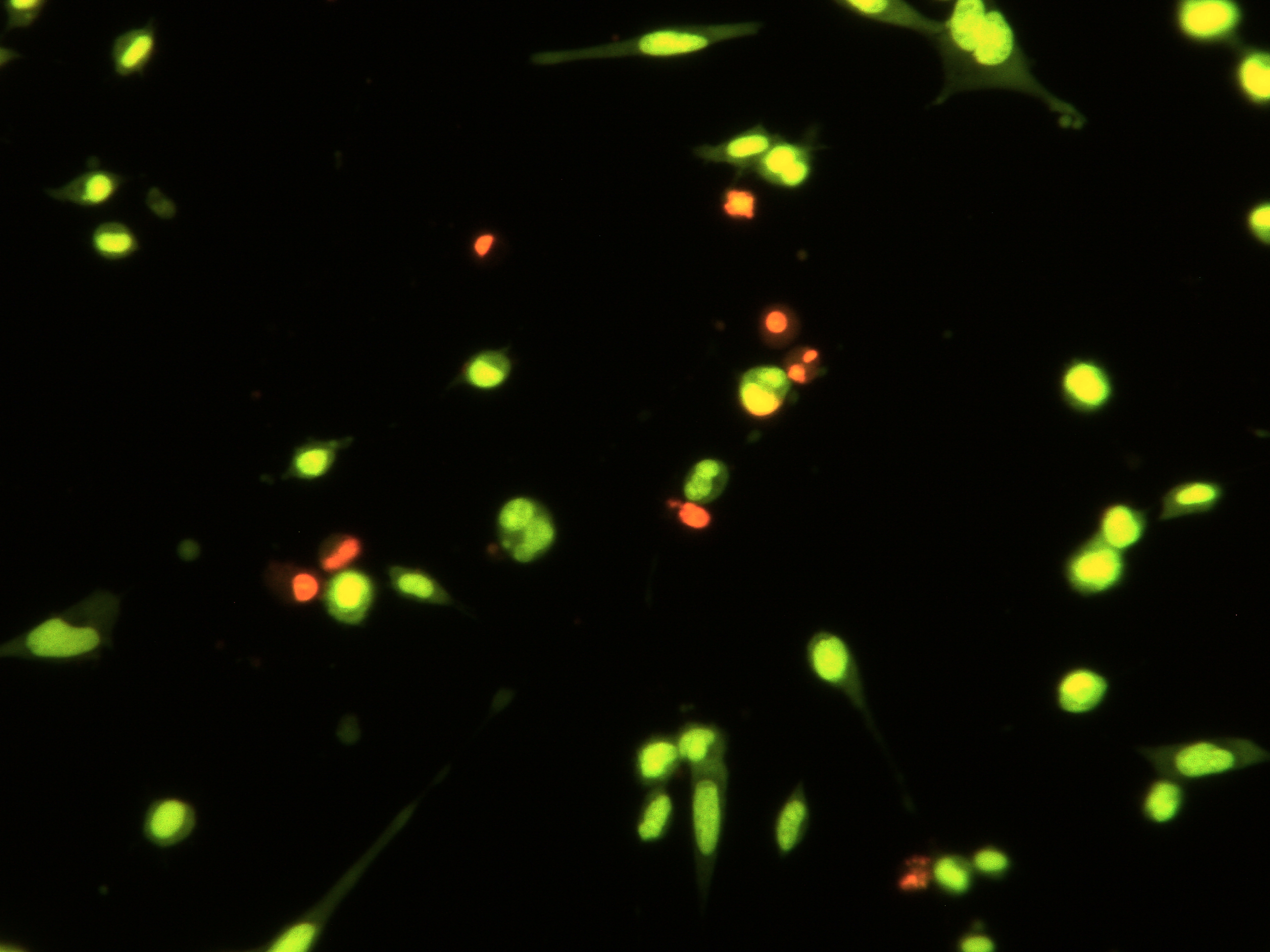

Supplement: S6 File — (ZIP) [file pone.0208866.s006.zip › S6_File/pone.0009826 EOC Replication Data 2018 (4 of 4)/wt ox200 0013.tif]

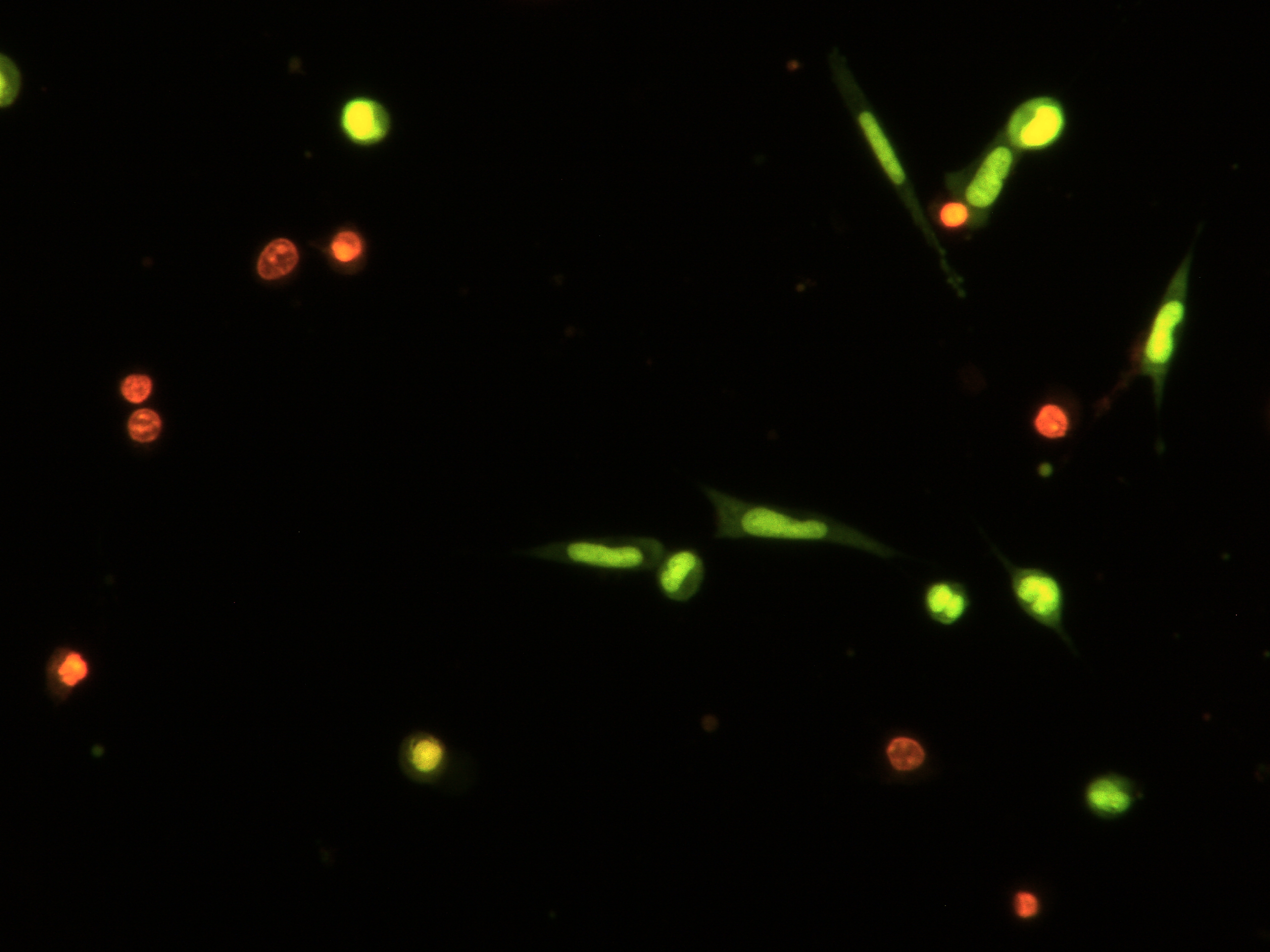

Supplement: S6 File — (ZIP) [file pone.0208866.s006.zip › S6_File/pone.0009826 EOC Replication Data 2018 (4 of 4)/wt ox200 0014.tif]

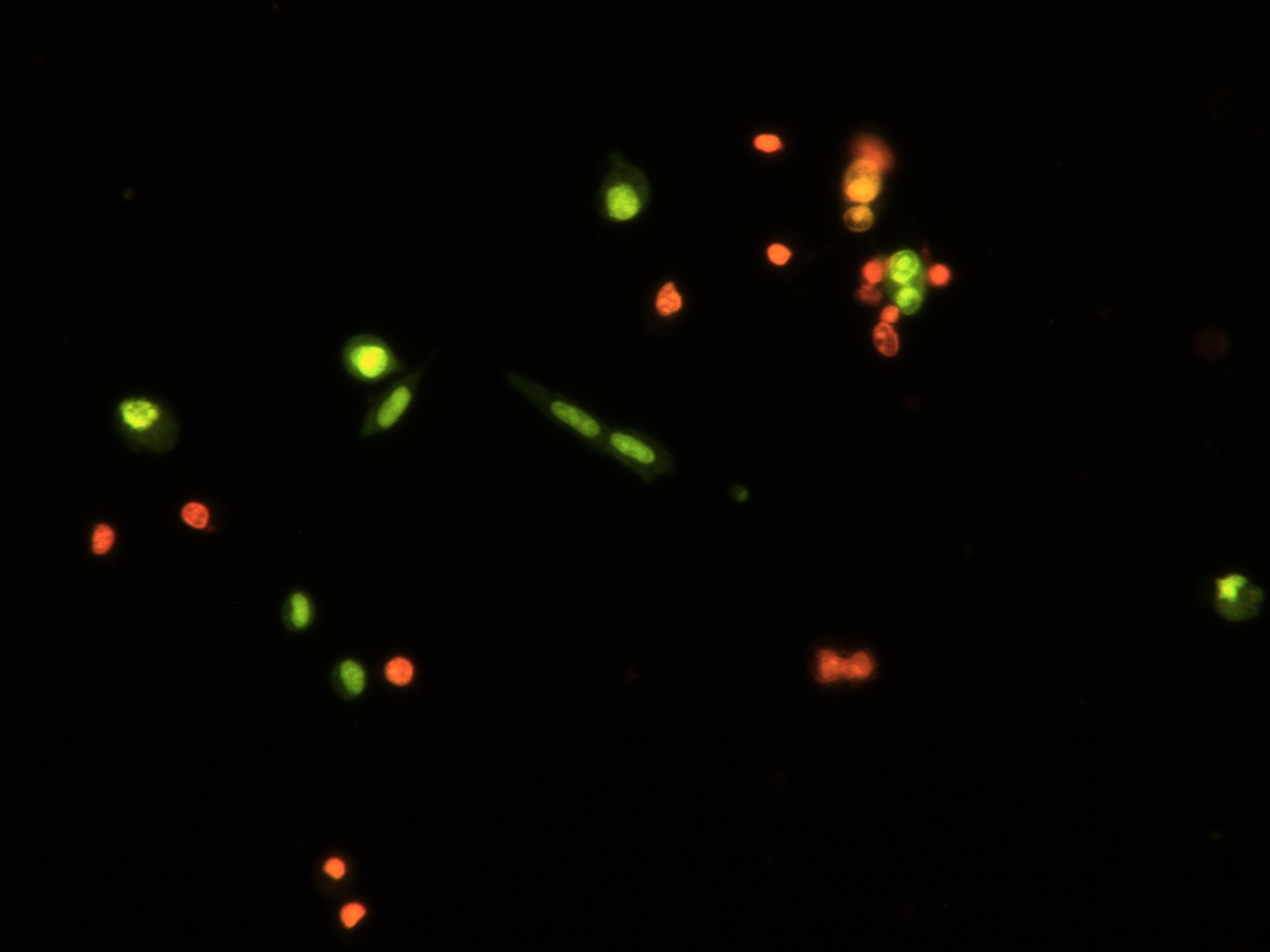

Supplement: S6 File — (ZIP) [file pone.0208866.s006.zip › S6_File/pone.0009826 EOC Replication Data 2018 (4 of 4)/wt ox200 0015.tif]

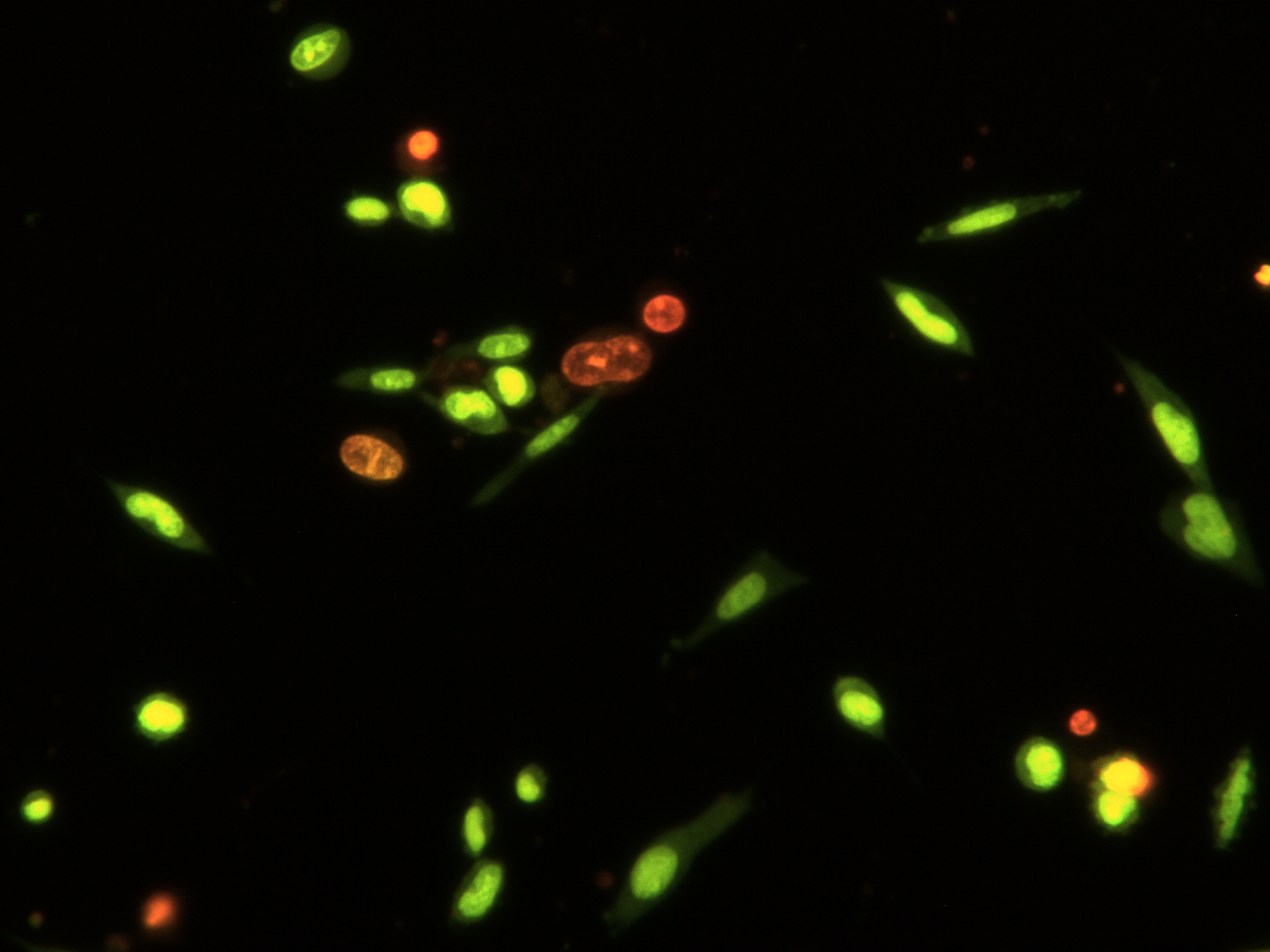

Supplement: S6 File — (ZIP) [file pone.0208866.s006.zip › S6_File/pone.0009826 EOC Replication Data 2018 (4 of 4)/wt ox200 0016.tif]

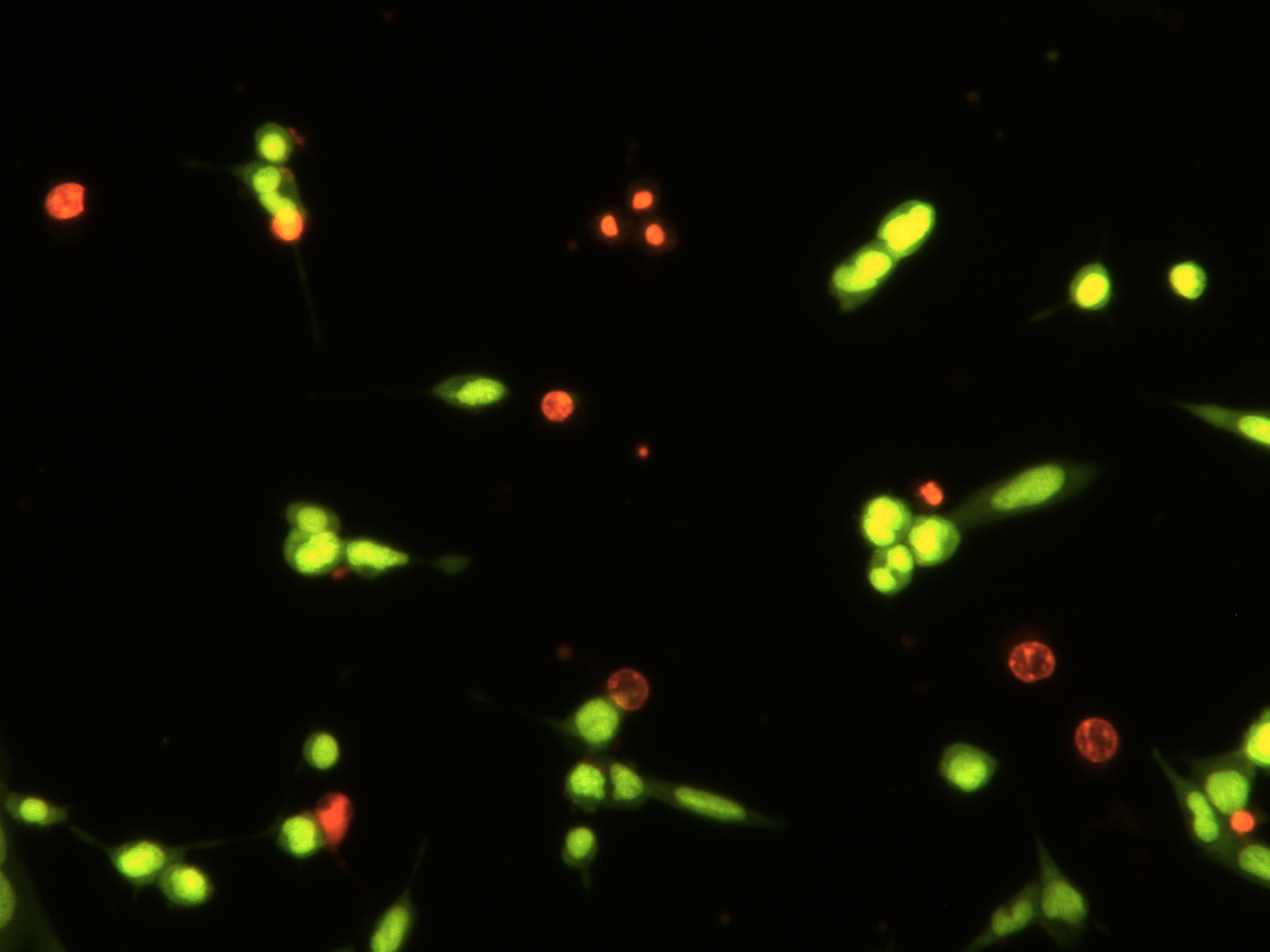

Supplement: S6 File — (ZIP) [file pone.0208866.s006.zip › S6_File/pone.0009826 EOC Replication Data 2018 (4 of 4)/wt ox200 0017.tif]

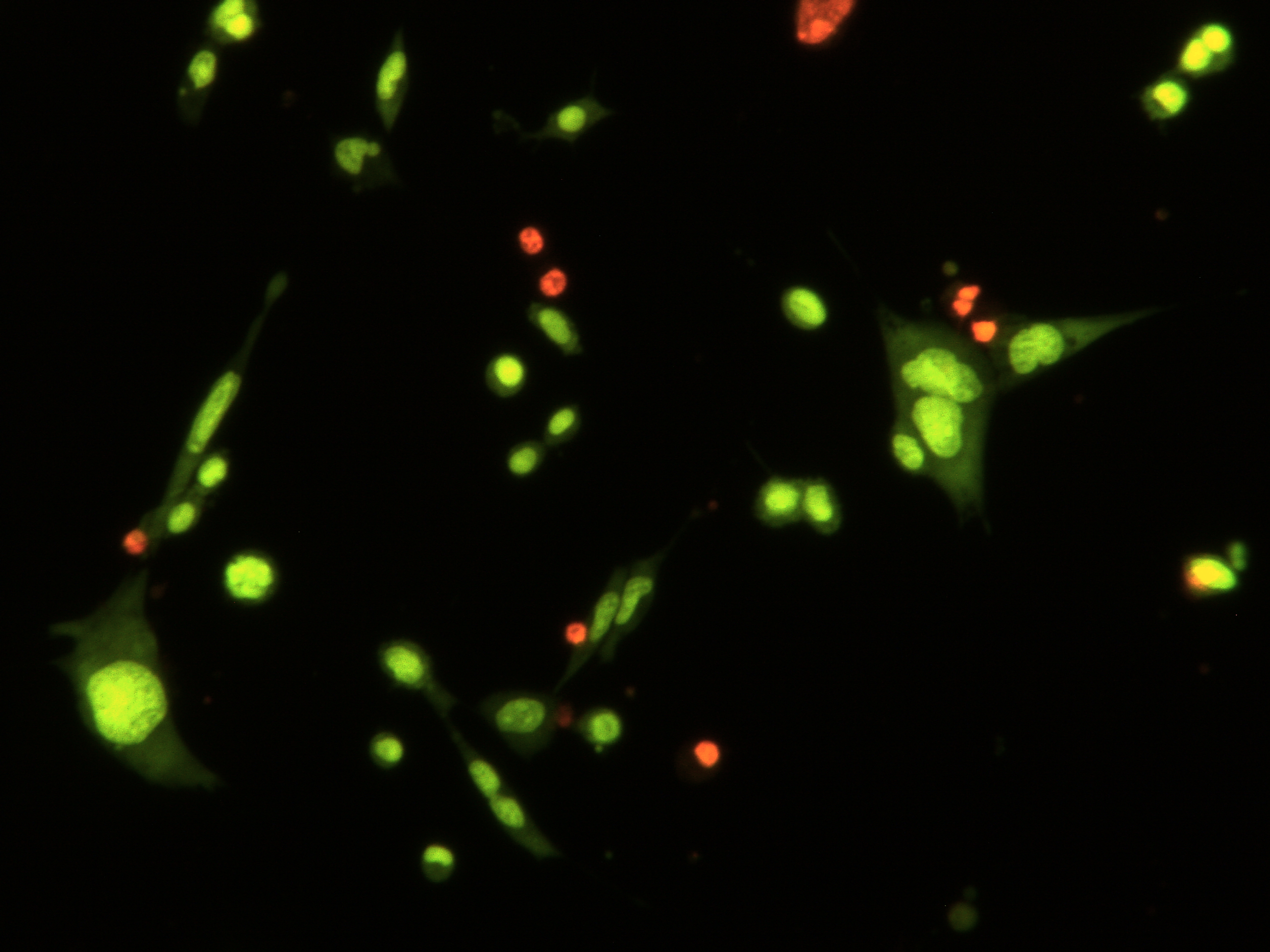

Supplement: S6 File — (ZIP) [file pone.0208866.s006.zip › S6_File/pone.0009826 EOC Replication Data 2018 (4 of 4)/wt ox200 0018.tif]

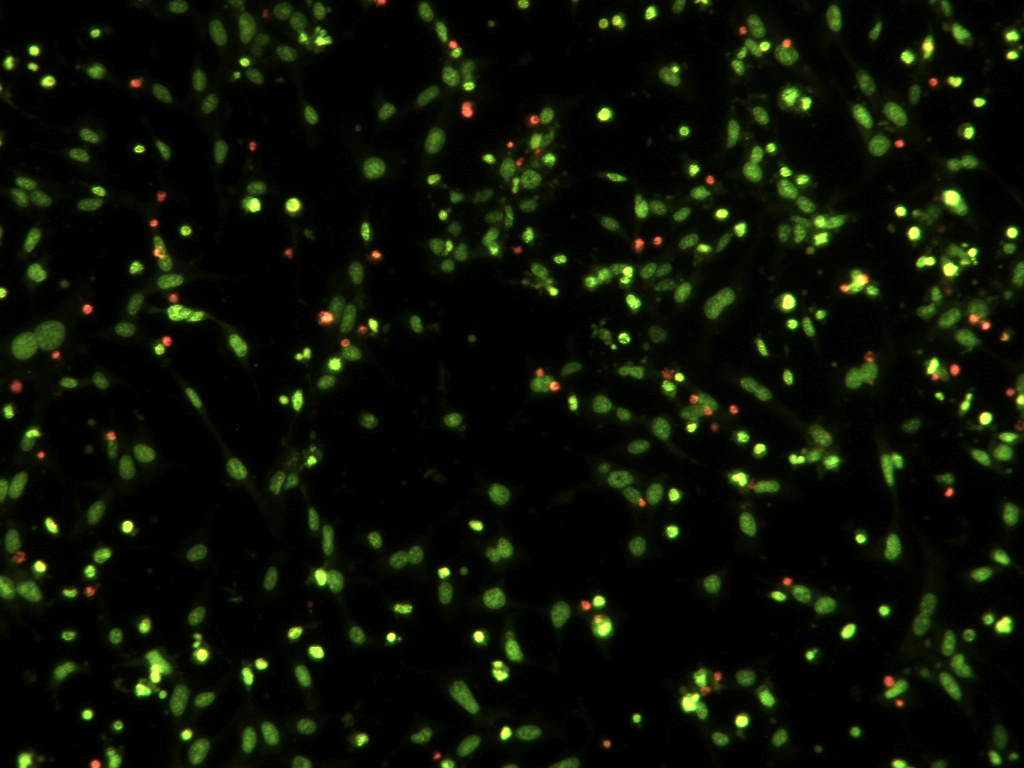

Supplement: S6 File — (ZIP) [file pone.0208866.s006.zip › S6_File/pone.0009826 EOC Replication Data 2018 (4 of 4)/wt stau 0001.tif]

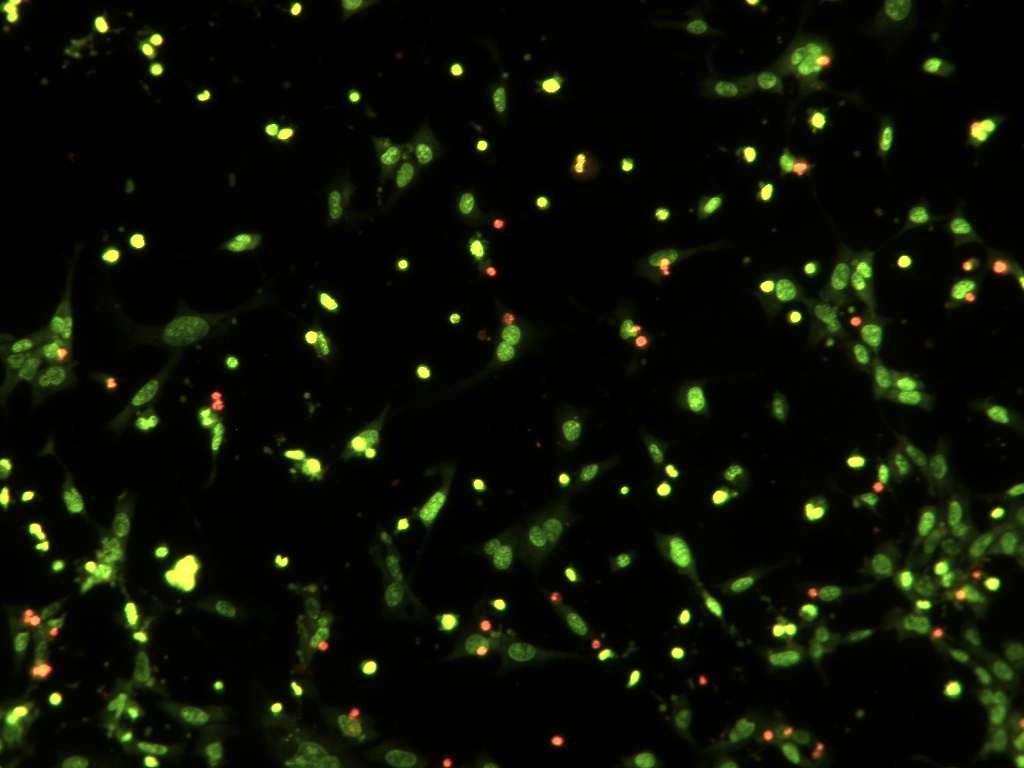

Supplement: S6 File — (ZIP) [file pone.0208866.s006.zip › S6_File/pone.0009826 EOC Replication Data 2018 (4 of 4)/wt stau 0002.tif]

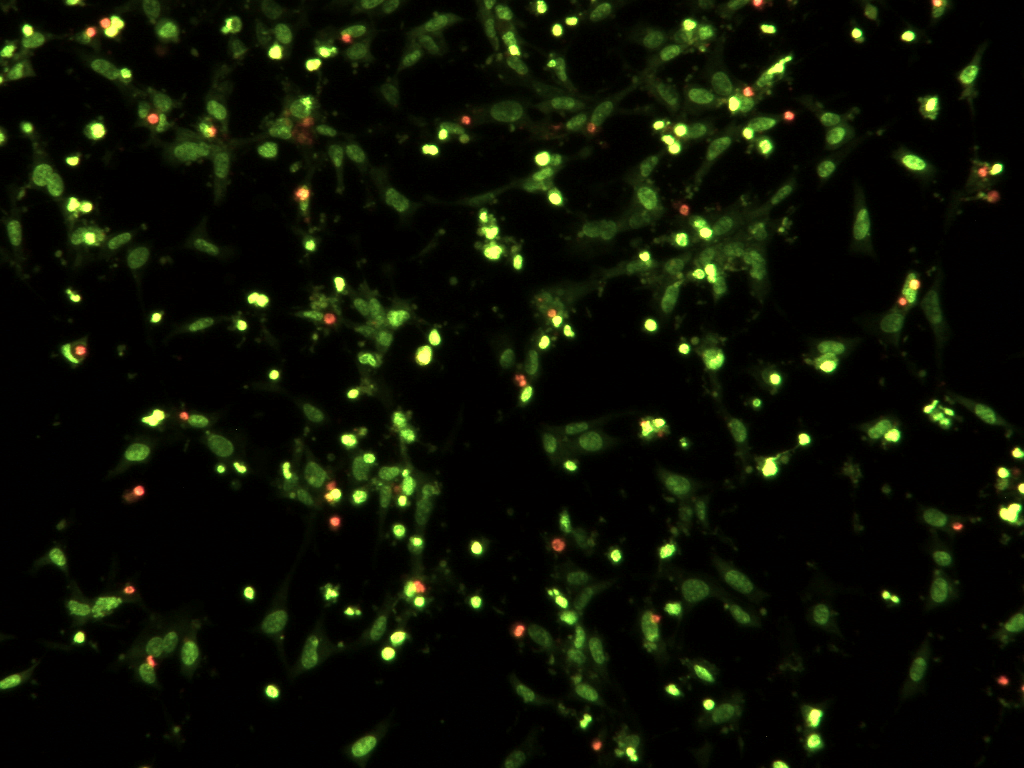

Supplement: S6 File — (ZIP) [file pone.0208866.s006.zip › S6_File/pone.0009826 EOC Replication Data 2018 (4 of 4)/wt stau 0003.tif]

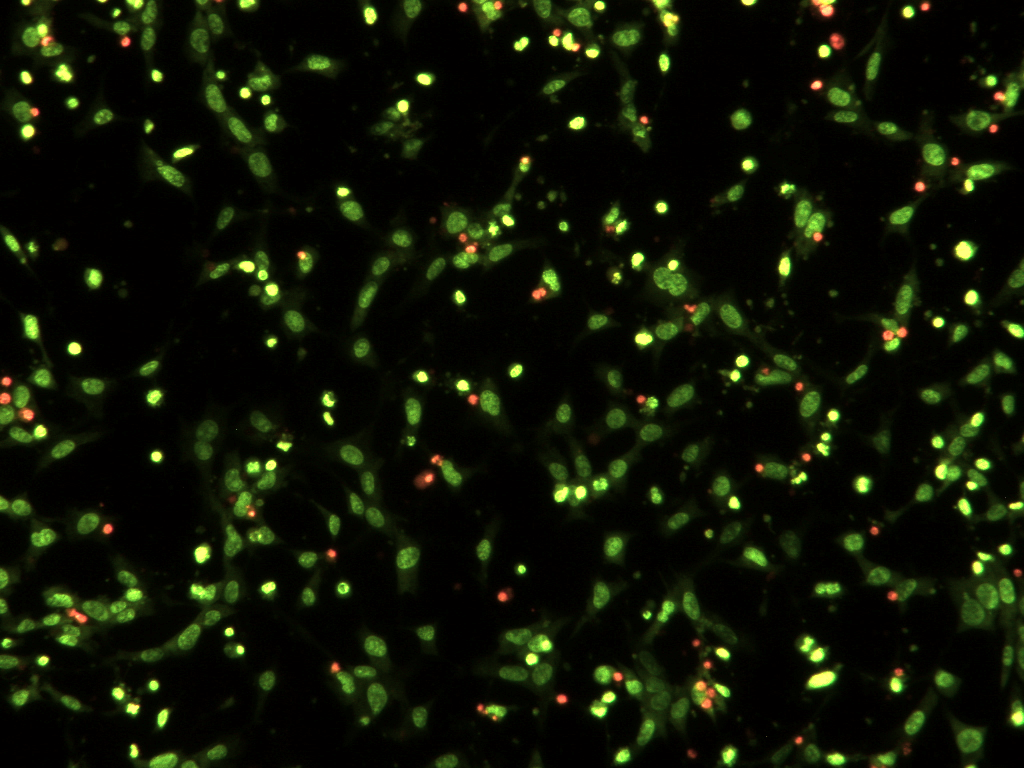

Supplement: S6 File — (ZIP) [file pone.0208866.s006.zip › S6_File/pone.0009826 EOC Replication Data 2018 (4 of 4)/wt stau 0004.tif]

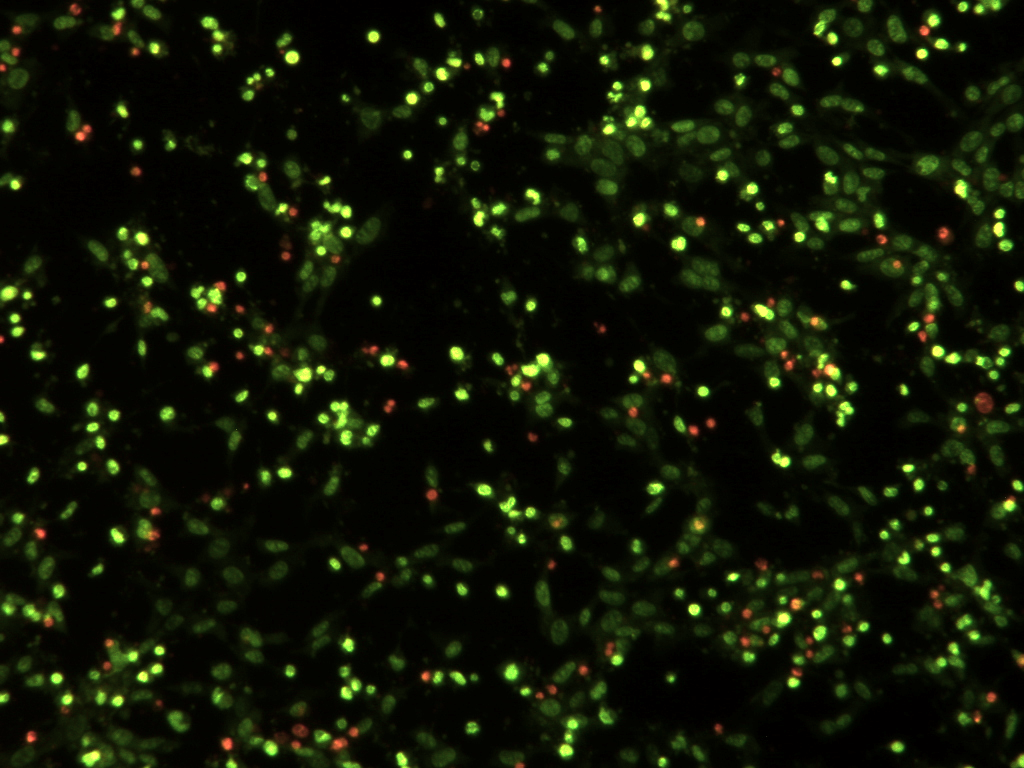

Supplement: S6 File — (ZIP) [file pone.0208866.s006.zip › S6_File/pone.0009826 EOC Replication Data 2018 (4 of 4)/wt stau 0005.tif]

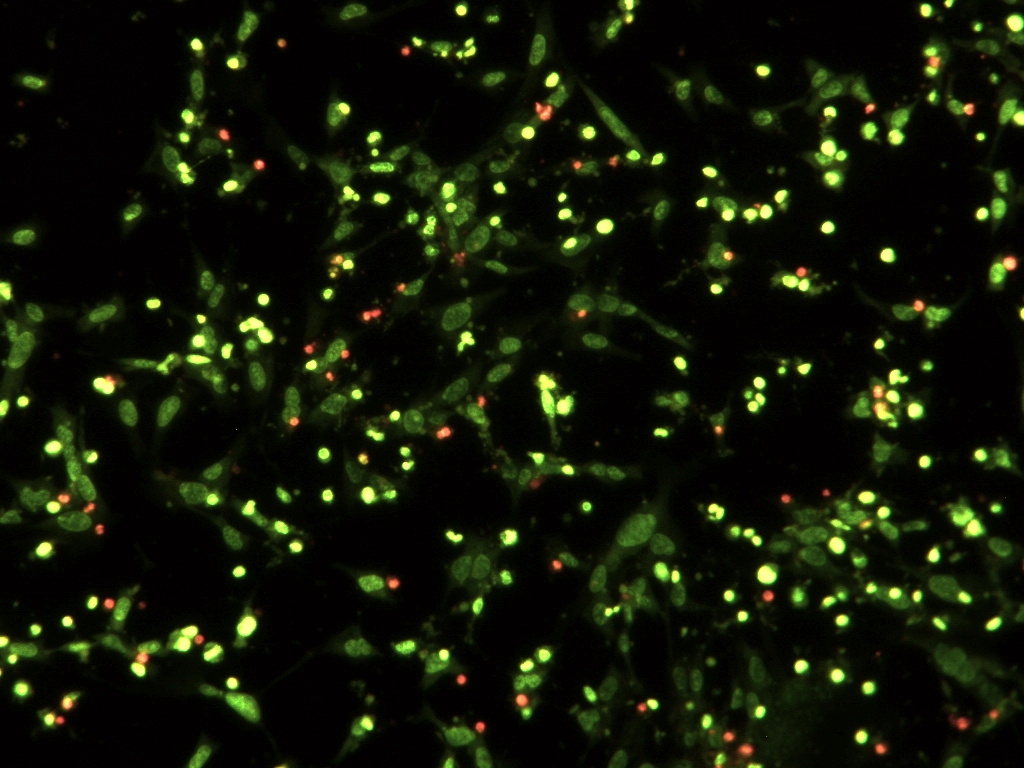

Supplement: S6 File — (ZIP) [file pone.0208866.s006.zip › S6_File/pone.0009826 EOC Replication Data 2018 (4 of 4)/wt stau 0006.tif]

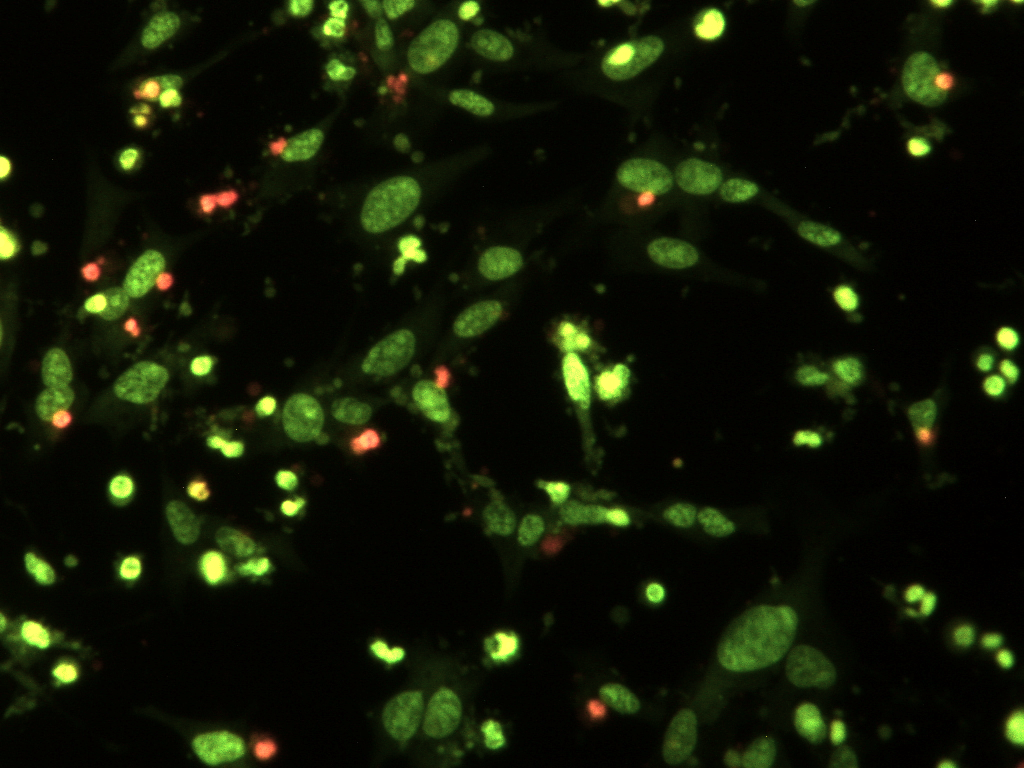

Supplement: S6 File — (ZIP) [file pone.0208866.s006.zip › S6_File/pone.0009826 EOC Replication Data 2018 (4 of 4)/wt stau 0007.tif]

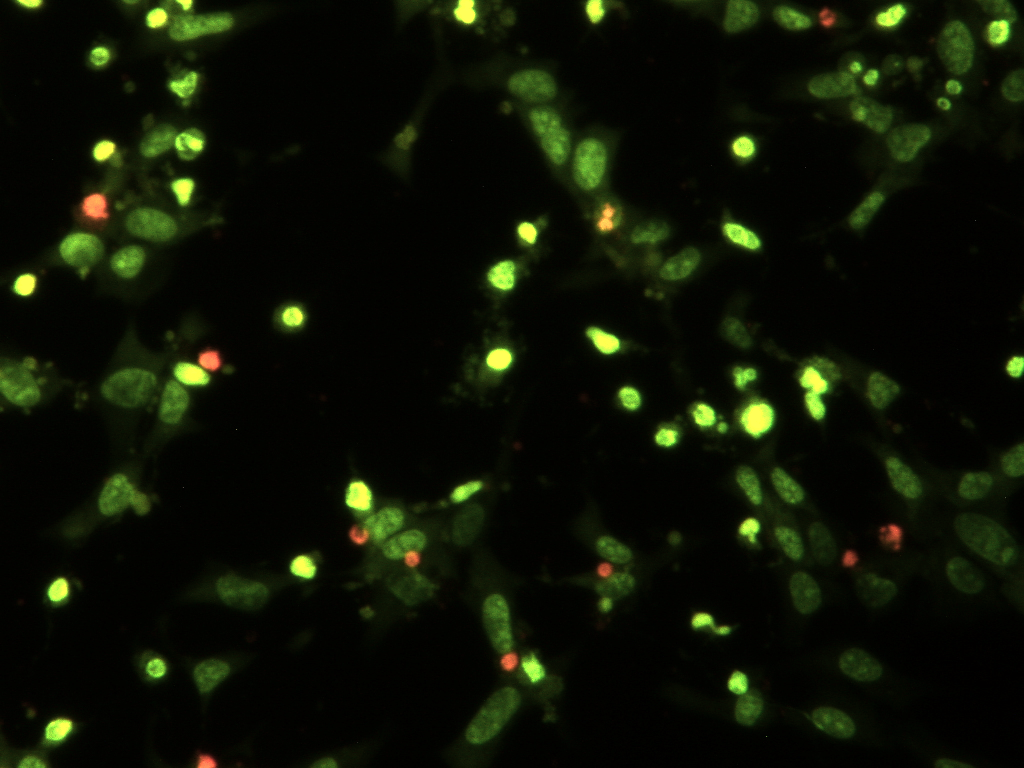

Supplement: S6 File — (ZIP) [file pone.0208866.s006.zip › S6_File/pone.0009826 EOC Replication Data 2018 (4 of 4)/wt stau 0008.tif]

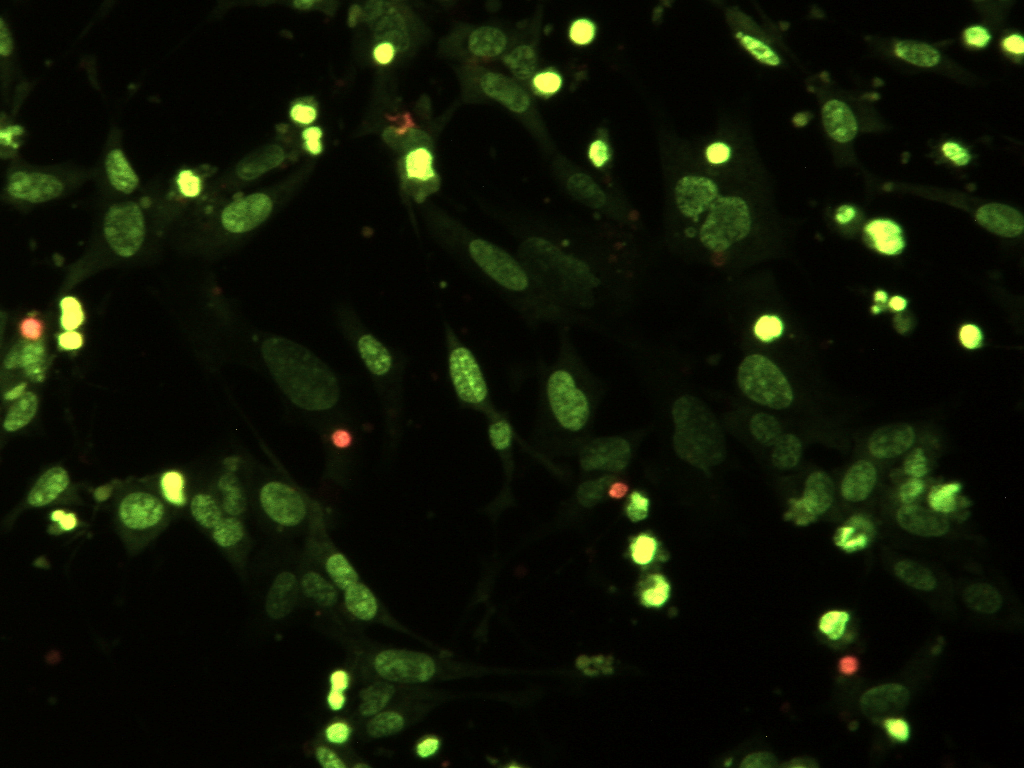

Supplement: S6 File — (ZIP) [file pone.0208866.s006.zip › S6_File/pone.0009826 EOC Replication Data 2018 (4 of 4)/wt stau 0009.tif]

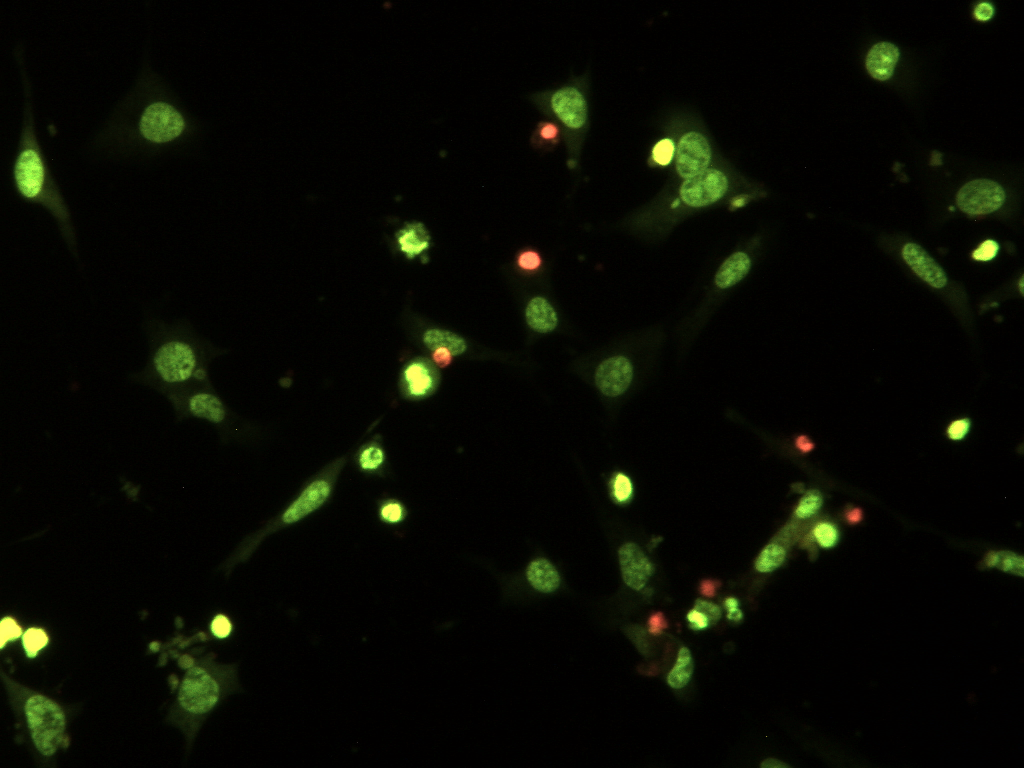

Supplement: S6 File — (ZIP) [file pone.0208866.s006.zip › S6_File/pone.0009826 EOC Replication Data 2018 (4 of 4)/wt stau 0010.tif]

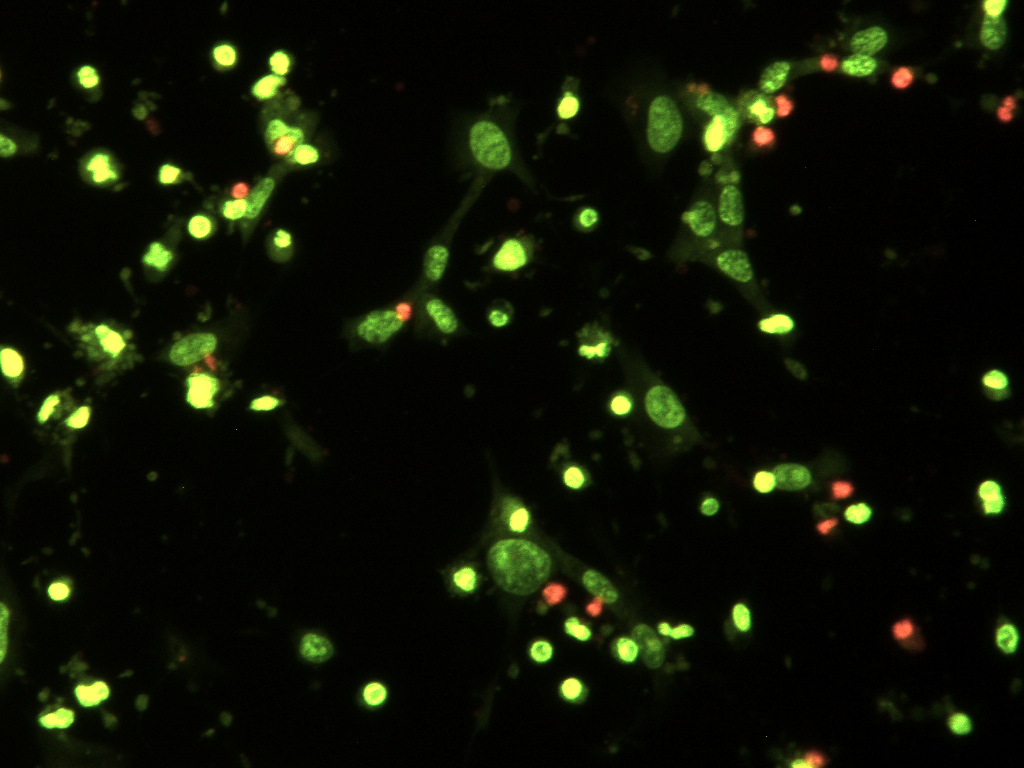

Supplement: S6 File — (ZIP) [file pone.0208866.s006.zip › S6_File/pone.0009826 EOC Replication Data 2018 (4 of 4)/wt stau 0011.tif]

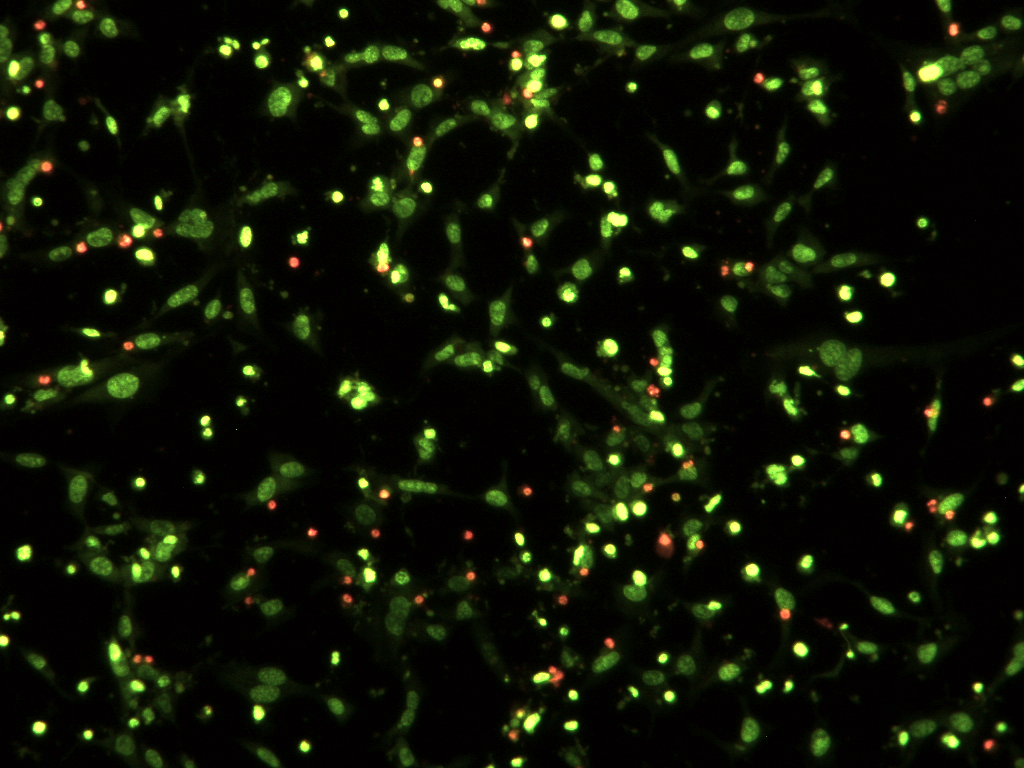

Supplement: S6 File — (ZIP) [file pone.0208866.s006.zip › S6_File/pone.0009826 EOC Replication Data 2018 (4 of 4)/wt stau.tif]
