# Supplementary material for: Expression of Concern: Stress-Induced Sphingolipid Signaling: Role of Type-2 Neutral Sphingomyelinase in Murine Cell Apoptosis and Proliferation
Source: PLoS One. 2018 Dec 10;13(12):e0208866. doi: 10.1371/journal.pone.0208866 (PMC6287835; doi:10.1371/journal.pone.0208866)
Supplement: S3 File — (ZIP) [file pone.0208866.s003.zip › S3_File/pone.0009826 EOC Replication Data 2018 (1 of 4)/Method for 2018 Replication.docx]

Apoptotic/necrotic cells were counted by fluorescence microscopy after staining by fluorescent DNA intercalating agents SYTO-13 and propidium iodide (PI) [exactly](http://journals.plos.org/plosone/article?id=10.1371/journal.pone.0009826#pone.0009826-Vieira1) as described in the original article. Briefly, cells grown in 6-multiwell plates were incubated with the permeant DNA intercalating green fluorescent probe SYTO-13 (0.6 µM) and the non permeant DNA intercalating red fluorescent probe PI (15 µM), using an inverted fluorescence microscope (Fluovert FU, Leitz). Intact, apoptotic and necrotic cells were characterized on the basis of their morphological features: Normal nuclei exhibit a loose green colored chromatin, nuclei of primary necrotic cells exhibit a loose red colored chromatin, nuclei of apoptotic cells exhibited fragmentation associated with condensed yellow/green-colored chromatin, while post-apoptotic necrotic cells exhibited the same morphological features, but were red-colored. 200 cells/well were counted. Pictures were taken using a camera (Leica microsystem) and cells were counted using image J^®^ software.

Please note that pictures named 'cnt' or 'wt cont' represent exactly the same experimental conditions ie wild type fibroblasts cultivated in standard conditions (without stress agent).
